# Optimization of the Synthesis and Conjugation of the Methyl Rhamnan Tip of *Pseudomonas aeruginosa* A-Band Polysaccharide and Immunogenicity Evaluation for the Continued Development of a Potential Glycoconjugate Vaccine

**DOI:** 10.1021/acsinfecdis.4c00049

**Published:** 2024-03-06

**Authors:** Mohammad P. Jamshidi, Chantelle Cairns, Nam Huan Khieu, Kenneth Chan, Frank St. Michael, Andrew Cox, Janelle Sauvageau

**Affiliations:** Vaccine and Emerging Infections Research, Human Health Therapeutics Research Centre, National Research Council, Ottawa, Ontario K1A 0R6, Canada

**Keywords:** Pseudomonas aeruginosa, vaccine, glycoconjugate, methylrhamnose, synthesis

## Abstract

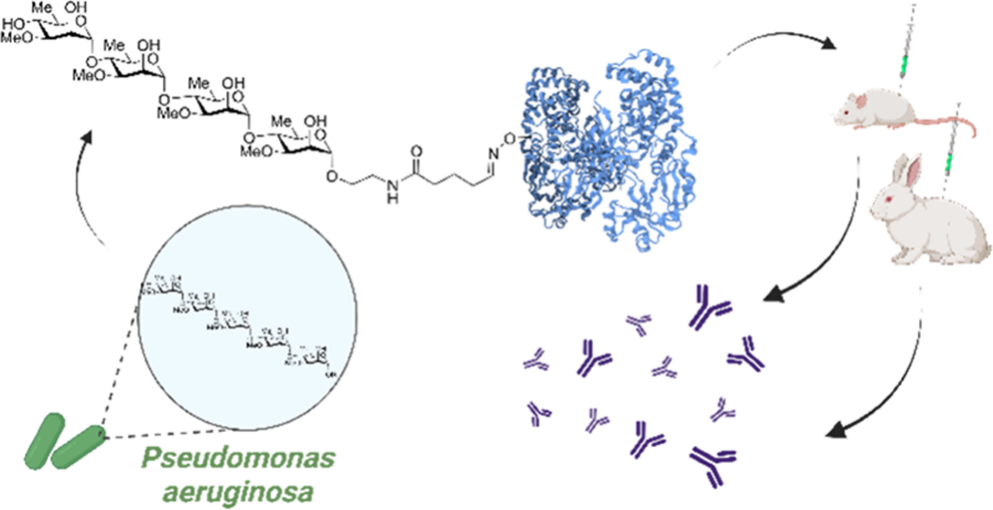

*Pseudomonas aeruginosa* is an antimicrobial-resistant bacterium that has no vaccine approved for human use. Additionally, it has been identified by the World Health Organization as a priority pathogen for novel vaccines and therapeutic development. We previously developed a synthetic mimic of the A-band polysaccharide tip that showed promise in terms of immunogenicity for use as a glycoconjugate vaccine. In this current manuscript, we improve upon the previous work to continue the development of this glycoconjugate vaccine. Herein, we report a higher-yielding synthesis of mimics containing a handle and a spacer that improved conjugation efficiency, resulting in better carbohydrate-to-protein ratios and also good immunogenicity of these conjugates in mice and rabbits. The data suggested that perhaps only a tetrasaccharide was required to induce an immune response capable of recognizing whole cells of *P. aeruginosa*.

*Pseudomonas aeruginosa* (*Pa*) is a pathogen that has become of considerable concern due to its resistance to most commonly used antibiotics, including carbapenems.^1^ This antimicrobial resistance (AMR) has prompted the World Health Organization and others to state that priorities include preventing infections by developing novel vaccines against AMR bacteria such as *Pa* due to the high mortality risk and the economic cost of these infections.^2,3^ Antibiotic-resistant *Pa* is often respirator-acquired and affects especially patients afflicted with cystic fibrosis, causing pneumonia and also a range of acute infections that can lead to sepsis.^4^ There is currently no vaccine on the market against *Pa*; however, a range of vaccines are being developed.^5,6^

Glycoconjugate vaccines are historically robust, safe, and efficient in preventing infections.^4,7−9^ When developing a glycoconjugate vaccine against a bacteria, a range of carbohydrates on the surface of the bacteria can be targeted, such as the capsule and lipopolysaccharides.^8^ In *Pa*, as the B-band polysaccharide is highly variable, while the A-band polysaccharide is conserved in many clinical isolates, the A-band polysaccharide could therefore be of interest to develop as an antigen to target for a vaccine.^10−12^

The A-band polysaccharide of *Pa* exhibits a methylated d-rhamnose pentasaccharide tip at its nonreducing end.^11^ In the study by Cairns et al., we have conjugated the natural antigen extracted from the A-band polysaccharide to a carrier protein and immunized mice and rabbits with this conjugate.^11^ This antigen is recognized by a mAb (1B1) generated from the natural pentasaccharide unit.^11^ As mAb 1B1 exhibits opsonophagocytic killing activity, the pentasaccharide tip is an interesting target antigen to further pursue as a vaccine.^11^ As the extraction of the natural antigen was low-yielding from the bacterial biomass, an alternative and potentially more efficient approach to generate this antigen for a glycoconjugate vaccine was synthesis.^12^ The synthesis of the oligosaccharide mimics was achieved with an overall yield of 3.6, 1.7, and 1% for the trisaccharide, tetrasaccharide, and pentasaccharide, respectively.^12^ The synthetically generated mimics were confirmed as inhibitors via inhibition ELISA. All three mimics inhibited the binding of mAb 1B1 to its target LPS, suggesting that all three could be potentially used as an antigen.^11^ This could be significant for future commercialization of a glycoconjugate vaccine as the yield to synthesize a pentasaccharide is significantly lower than to synthesize a trisaccharide. All three mimics were conjugated to human serum albumin (HSA) via direct reductive amination of the anomeric aldehyde and led to 6 oligosaccharide units per HSA.^12^ The resulting conjugates elicited good IgM and IgG-specific responses in mice.^12^

Despite the encouraging results obtained from this original work, we strived to improve our conjugates to increase their immunogenicity. At first, we aimed to achieve the conjugation of the mimics to cross-reactive material 197 (CRM_197_), as opposed to HSA, as CRM_197_ is widely used in the clinic and is generally regarded as a safe and effective carrier protein.^13^ Direct conjugation of a saccharide to a protein has the advantage of reducing the number of synthetic steps required and not introducing potential additional linker molecules that could elicit an immune response. However, factors such as steric hindrance, distance between the epitope and the carrier, and reactivity of the anomeric aldehyde for direct reductive amination can impact the effectivity of the conjugation negatively.^14,15^ A wide range of handles and spacers have been described in the past; aminoalkyl glycosides, in particular, have been widely used, attached at the anomeric position of a range of carbohydrates, as handles in the context of glycoconjugate vaccines.^16,17^ We thus envisioned that adding a handle (Figure 1) to the reducing end of the mimics might allow us to use different linking strategies to achieve improved conjugation to CRM_197_ and with a higher carbohydrate-to-protein ratio than in our previous work (6 carbohydrates per HSA protein). Such strategies include activating CRM_197_ to its aminooxy form, for example.^18,19^ This potentially higher ratio might also increase the immunogenicity of the conjugate.

**Figure 1:**
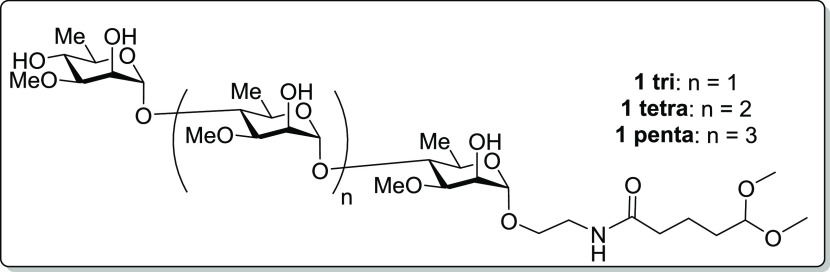
A-band tip polysaccharide mimics.

Herein, we report the synthesis of the A-band polysaccharide tip mimics composed of 3-*O*-methyl-d-rhamnose repeats, including a trisaccharide, tetrasaccharide, and pentasaccharide with a handle at the reducing end. We implemented the usage of a heterobifunctional spacer composed of an aldehyde protected with a hemiacetal and an activated *N*-hydroxysuccinimide ester, as described by Pozsgay.^20^ We also report improvements to synthetic yields and a reduction in the number of steps for early intermediates. Additionally, an improved degree of conjugation of the generated mimics to CRM_197_ after the derivatization of CRM_197_ to its aminooxy form is reported. Finally, we evaluate the immune response to the CRM_197_ mimics conjugates in mice and rabbits, establish immunogenicity, and potentially identify the minimum length of oligosaccharide that effectively mimics the natural antigen. We also evaluated whether rabbit and mouse sera bind to whole killed cells from a range of strains, including clinical isolates.

## Results and Discussion

**Scheme 1:**
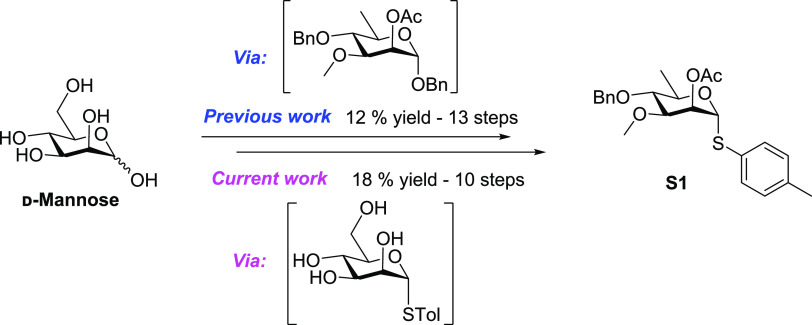
Previous vs Current Work to Access Advanced Glycosyl Donor *p*-Tolyl 2-*O*-Acetyl-4-*O*-benzyl-3-*O*-methyl-1-thio-α-d-rhamnopyranoside (**S1**)

To add a handle at the reducing end of the pentasaccharide tip mimics, we had to restock intermediate **S1**. Previously, the 3-*O*-methyl-d-rhamnose mimics we had generated had been synthesized using 1-*O*-benzyl intermediates (Scheme 1); however, late-stage deprotection of the anomeric benzyl group with HCl in acetonitrile proved challenging with a 65% yield, resulting in a low overall yield for the synthesis of **S1**.

It was envisioned that instead, a *p*-tolyl thioglycoside could be first generated with the *p*-tolyl group as an anomeric protecting group throughout the synthesis as well as a glycosyl donor during late-stage glycosylation as previously implemented by others.^21,22^

To start, acetylated d-mannose obtained as described previously^12^ was converted to thioglycoside **S2** in two steps with excellent yields by treatment with thiocresol under acidic conditions followed by acetate deprotection using Zemplén deacetylation. C6 deoxygenation was achieved, as previously reported, via the Appel reaction (**S3**), followed by reduction with palladium hydroxide to obtain d-rhamnose thioglycoside **S4** in moderate yields (Scheme S1). Next, 2,3-*O*-acetonide protection (**S5**) and *O*-4 benzylation lead to acetonide **S6** in 90% yield over two steps.

After acetonide deprotection (**S7**), regioselective *O*-3 methylation was achieved in high yields via the formation of a tin acetal and selective methylation using methyl iodide and cesium fluoride, giving 3-*O*-methyl thioglycoside **S8**. The presence of methyl at 3 was confirmed via heteronuclear multiple bonds coupling of the *O*-Me ^1^H singlet at 3.51 ppm and C3 at 82.0 ppm. Lastly, the thioglycosyl donor **S1** was isolated in 89% yield after *O*-2 acetylation. The *p*-tolyl 1-thio group remained intact throughout the synthesis, allowing access to key intermediate **S1** with 50% greater efficiency and three steps shorter compared to our previous synthesis.

**Scheme 2:**
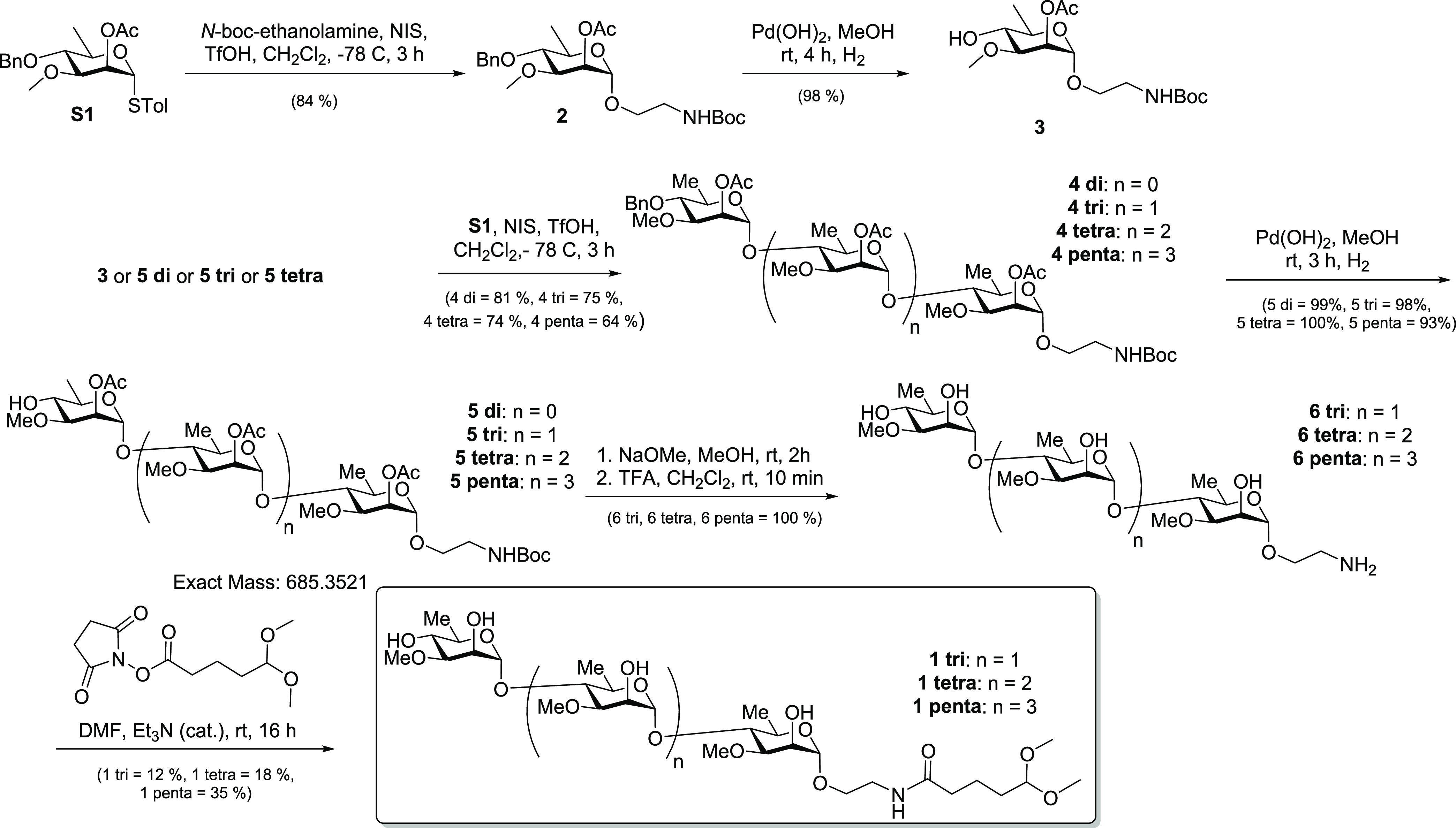
Synthesis of 3-*O*-Methyl-d-rhamnose Mimics with Handle (**1**)

With **S1** in hand, *N*-boc-protected monomer, **2**, was formed via glycosidic linkage with the glycosyl donor, *N*-boc-ethanolamine (Scheme 2). This was achieved in 84% yield by activating donor **S1** with NIS and TfOH (HRMS *m*/*z* calcd for C_23_H_35_NO_8_H [M + H]^+^, 454.2435; found, 454.2435). Glycosyl acceptor **3** was formed in excellent yields by addition to a stirred solution of activated Pearlman’s catalyst in methanol under hydrogen gas. Iterative 1,4 glycosylations between 4-*O* deprotected handles (**5** di, **5** tri, **5** tetra) and building block **S1** generated oligosaccharide handles (**4** di, **4** tri, **4** tetra, and **4** penta). 4-*O*-Benzyl deprotections lead to near quantitative yields. Glycosylation yields were higher than what we previously observed with the benzyl glycoside as an acceptor, with all yields being above 64%, while we previously observed yields below 69%. The α configuration was exclusively observed consequent with prior work and as verified by the *J*_H1–C1_ coupling constants.^12^

Oligosaccharides **5** tri, **5** tetra, and **5** penta were globally deprotected using sodium methoxide followed by TFA for Boc deprotection, yielding the free amino handles **6** tri, **6** tetra, and **6** penta (ESI-MS: *m*/*z* calcd for C_37_H_67_O_21_NH [M + H]^+^, 862.4278; found, 862.4265) in quantitative yields. Without further purification, these free handles were then conjugated to an activated *N*-hydroxysuccinimide ester spacer containing a protected aldehyde. HPLC purification using C-18 produced pure antigens **1** tri, **1** tetra, and **1** penta in respectively 1.2, 1.4, and 1.6% yield for evaluation in colorimetric inhibition ELISA assays and protein conjugation.

### Inhibition ELISA Assay

To ensure that the necessary conformation to mimic the natural antigen had been retained in the synthetic oligosaccharides with the handle and spacers, **1** tri, **1** tetra, and **1** penta were evaluated in an inhibition ELISA against mAb 1B1 that was raised to the natural antigen.^12^

In this colorimetric inhibition ELISA, we observed a reduction in absorbance for all of the saccharides, indicating that all bind to mAb 1B1 (Figure S1), with **1** penta blocking mAb 1B1 at a greater extent than **1** tetra than in turn **1** tri.

These results therefore corroborate our prior data on prepared oligosaccharides (without handle and spacer).^11,12^ The addition of the handle and spacer in these oligosaccharides does not alter the conformation of the oligosaccharide nor affect the oligosaccharide from binding and blocking mAb 1B1. Thus, the synthesized oligosaccharides with a handle and spacer effectively mimic the epitope recognized by mAb 1B1 on *Pa*.

### Conjugation of Oligosaccharide to Activated CRM_197_ and Activated BSA to Prepare Glycoconjugates

**Scheme 3:**
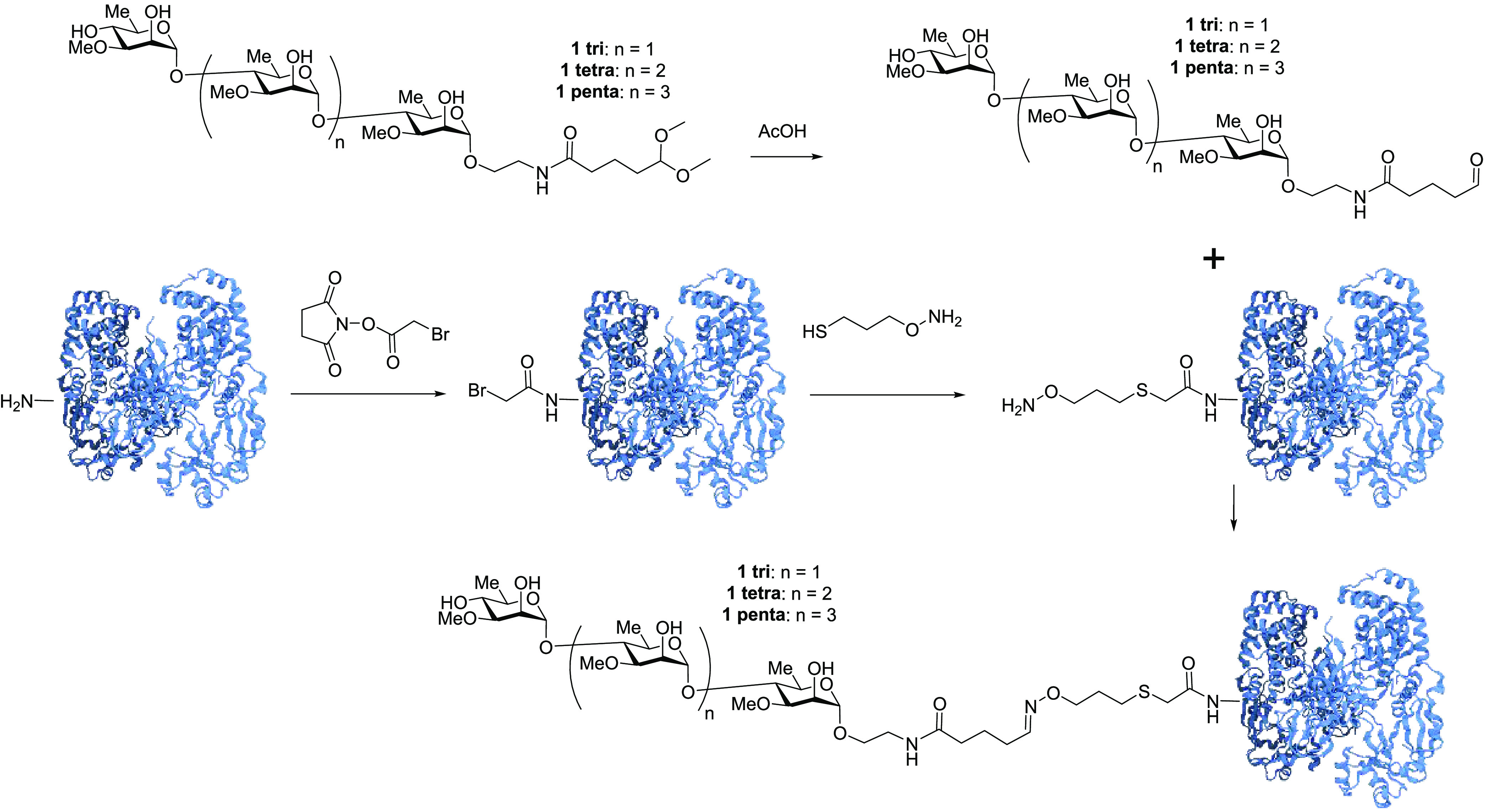
Deprotection of **1** tri, tetra, and penta and Conjugation to Aminooxy-Activated CRM_197_

Lysines on CRM_197_ and BSA were first modified to their bromoacetic amide derivatives. Then, a reaction with (aminooxy)-1-propanethiol led to aminooxy-activated CRM_197_/BSA. The conjugates were prepared from **1** tri, **1** tetra, and **1** penta after deprotection of the hemiacetal to the aldehyde using acetic acid and formation of the stable oxime after conjugation to the aminooxy-activated CRM_197_/BSA (Scheme 3). The carbohydrate/protein ratio, as determined by MALDI-MS analyses, revealed a satisfactory ratio between 5 and 6 for BSA and a good ratio between 8 and 10 for CRM_197_ (Table S1). Even though the results obtained with BSA were similar to our prior work (albeit with HSA), conjugation to CRM_197_ was improved with 8–10 saccharides as opposed to 6 (for HSA).

### Screening of Derived Mice Sera vs BSA Conjugates and LPS

To determine whether the new conjugates were immunogenic, the mice were immunized under a prime and two boosts schedule. We evaluated whether the BSA–oligosaccharide conjugates and *Pa* wt LPS were recognized by individual mice sera and compared the responses to the preimmune sera via ELISA.

All mice produced a good IgM response, albeit a moderate IgG response both specific to the carbohydrates and *Pa* wt LPS (Figures S2–S11). Since mice that received immunizations with the CRM-**1** tetra and **1** penta conjugates showed an improved IgG response to *Pa* wt LPS in ELISA relative to mice that received the CRM-**1** tri, it may be suggested from the mice data that the minimum length of oligosaccharide required to effectively mimic the natural antigen is a tetrasaccharide (Figures S2–S11).

### Screening of Derived Rabbit Sera vs BSA Conjugates and LPS

**Figure 2:**
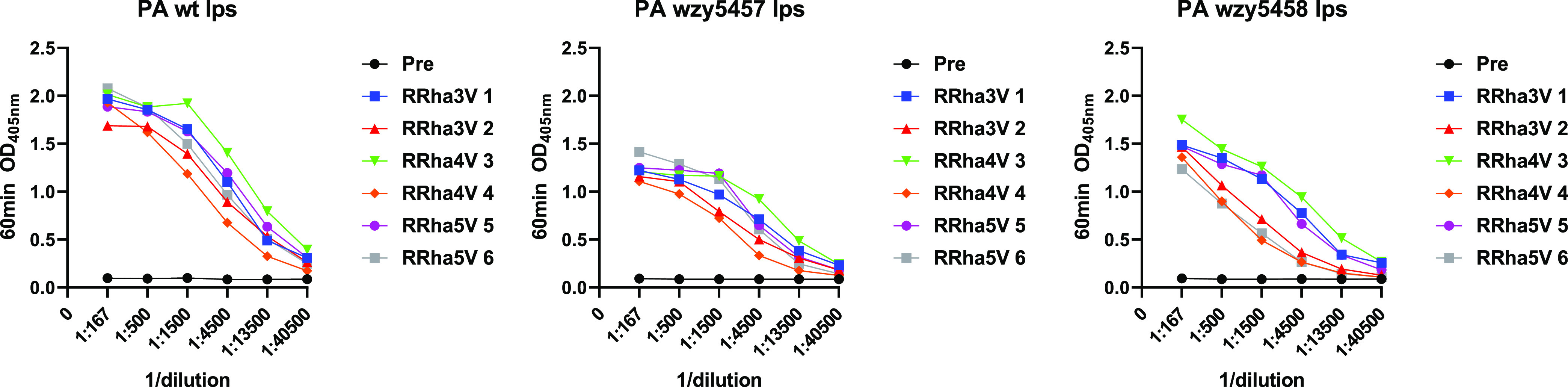
ELISA determined recognition with pre- and postimmune (D70) rabbit sera titration prior to and following CRM-oligosaccharide conjugate immunization vs *Pa* wt, Pa wzy5457, and Pa wzy5458 LPS. Rabbits RRha3V 1–2 received the trisaccharide CRM conjugate, rabbits RRha4V 3–4 received the tetrasaccharide CRM conjugate, and rabbits RRha5V 5–6 received the pentasaccharide CRM conjugate.

With the encouraging mice results described above, we elected to evaluate the capacity of the conjugates to elicit a specific immune response in rabbits. As for mice, we evaluated whether the BSA–oligosaccharide conjugates and *Pa* wt LPS were recognized by individual rabbit sera. All rabbits produced a good immune response to the conjugates, as illustrated by their recognition of the BSA–oligosaccharide conjugates and *Pa* wt LPS. Differently from mice that exhibited a modest response, all rabbits produced a strong response with end-point titers in the 1:10,000 range that were capable of recognizing three LPS structures (*Pa* wt, Pa wzy5457, and Pa wzy5458 LPS) (Figures 2 and S12).

### Screening of Derived Mice and Rabbit Sera vs Killed Whole Cells

To verify whether postimmune mice and rabbit sera would recognize a range of strains, which is important to evaluate the strain coverage ability of the conjugates, whole-cell ELISA was performed on a range of *Pa*-killed cells (Table S2), including a wt strain, strains with mutations in their genes thought to be related to the A-band methyl rhamnan, serotype strains most commonly encountered in a clinical setting, and a small group of clinical isolates.

Postimmune mice sera pooled by the conjugate that they received showed recognition of approximately half the cells they were tested against (Figure S13). The CRM-**1** tri conjugate showed a weaker response when compared to the clear-cut response provoked by the CRM-**1** tetra and CRM-**1** penta conjugate, illustrating that the methyl rhamnan tip epitope is visible in the context of the whole cells and that the synthetic conjugate was able to generate an immune response capable of recognizing this epitope elaborated on whole cells.

**Figure 3:**
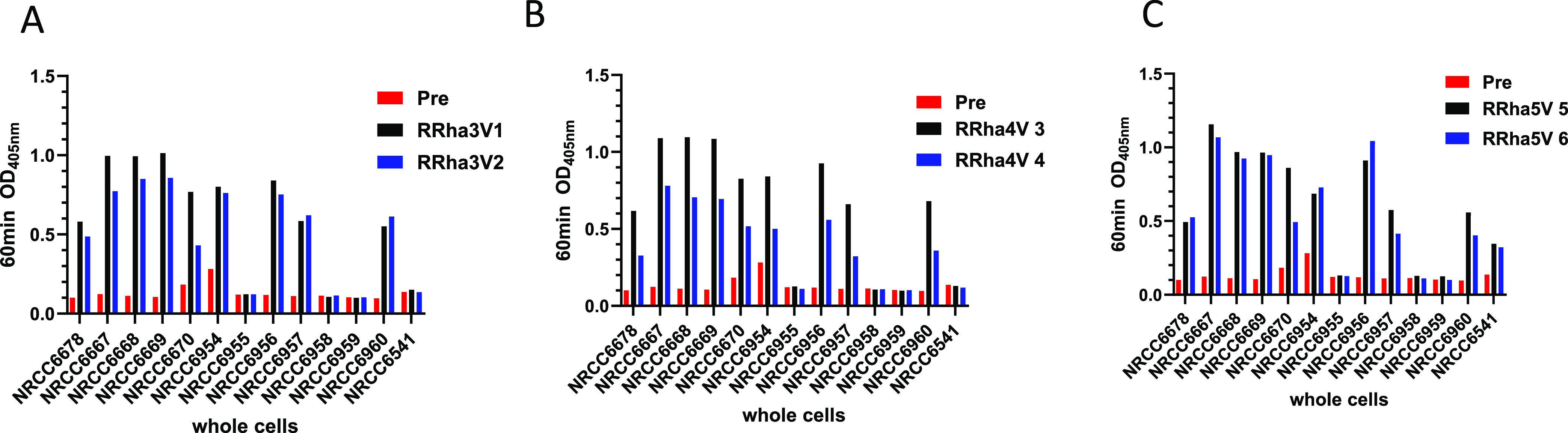
ELISA analysis of binding of individual rabbit sera. (A) RRha3V 1–2 received the trisaccharide CRM conjugate. (B) RRha3V 1–2 received the tetrasaccharide CRM conjugate. (C) RRha3 V1–2 received the pentasaccharide CRM conjugate; pre- and postimmune (all at 1:1500 dilution) to killed whole cells of *P. aeruginosa* strains NRCC #’s 6678, 6667–70, 6954–60. and negative control strain M. catarrhalis 6541 (see Table S2 for full details of strains).

When postimmune rabbit sera were evaluated (Table S2 and Figures 3 and S14), a majority of the range of cells, including against a range of clinical isolates in our collection, were recognized, illustrating that the methyl rhamnan tip epitope is visible in the context of whole cells and that the conjugates (**1** tri, **1** tetra, and **1** penta) were able to generate an immune response capable of recognizing this epitope, also illustrating the conservation of this epitope and the ability of oligosaccharide-based conjugates to be capable of generating the required immune response to afford recognition.

Consistent with the earlier data with LPS and BSA conjugates, whole-cell ELISA corroborated the requirement in mice for a tetrasaccharide as the minimum length of oligosaccharide necessary to facilitate an appropriate response. However, rabbit-derived sera did not exhibit this same minimum length requirement, as all conjugates were able to elicit a similar cross-reactive response.

## Conclusions

Herein, we reported the synthesis of three targets: 3-*O*-methyl rhamnan trisaccharide, tetrasaccharide, and pentasaccharide, with an aminoethyl handle at the reducing end to create a glycoconjugate vaccine containing a mimic of the A-band polysaccharide tip of *Pa*. This improved synthesis demonstrated an increase in overall yield compared to our previous route (1.6% vs 1.0% overall yields for the pentasaccharide, for example) and with an added handle and spacer. Additionally, the addition of an amino handle at the reducing end of the sugar allowed for improved conjugation to CRM_197_, as exemplified by conjugates with carbohydrate loading of approximately 10 residues obtained for each oligosaccharide.

The results obtained with the immune sera from mice and rabbits indicate that CRM_197_ conjugates of at least the synthetic **1** tetra and **1** penta representative of the methyl rhamnan A-band tip epitope are capable of provoking a specific immune response that recognizes the natural antigen elaborated on *Pa* wt LPS and whole cells representing the most commonly encountered serotypes in a clinical setting, and from a small collection of clinical isolates, illustrating their potential as viable alternatives to the isolated antigens as vaccine immunogens. Continued development will include performing opsonophagocytic assays with the polyclonal mice and rabbit sera and subsequently animal models.

There remains a shortfall of vaccines to combat the significant AMR threat of *Pa*, and this work has highlighted that these relatively simple oligosaccharides provide a potential synthetic route to an antigen for consideration as an alternative to antibiotics to combat this challenge.

## Methods

Reactants and reagents were purchased from MilliporeSigma, Oakwood Chemicals, or Fisher Scientific and used without further purification. NMR spectra were measured on either a Varian (^1^H 500 MHz, ^13^C 125 MHz), Bruker (^1^H 600 MHz, ^13^C 150 MHz), or Jeol (^1^H 400 MHz, ^13^C 100 MHz) spectrometer. Spectra were calibrated with the solvent’s residual signals (CDCl_3_, 7.26 ppm for ^1^H and 77.1 ppm for ^13^C; CD_3_OD, 3.31 ppm for ^1^H and 49.0 ppm for ^13^C). NMR signals were assigned using standard two-dimensional NMR experiments (HSQC, COSY, HMBC). Purification occurred on CombiFlash RF and Biotage system with Silicycle silica columns. Mass-spectrometry data was recorded and analyzed using a single quadrupole detector 2 from Waters, and high-resolution mass-spectrometry data were recorded and analyzed on a Waters Ultima using the LC/MS Calibrant Mix from Agilent as the internal standard. TLC was eluted using hexane/ethyl acetate or MeOH/CH_2_Cl_2_ as solvents and visualized with charring after dipping in a 5% H_2_SO_4_ solution in absolute ethanol.

### 2-*O*-Acetyl-4-*O*-benzyl-1-*O*-[(*N*-ethyl)-*tert*-butoxycarbonyl]-3-*O*-methyl-α-d-rhamnopyranoside (**2**)

**S1** (46.4 mg, 0.11 mmol) and *N*-boc-ethanolamine were coevaporated together with toluene (5 × 10 mL) and left to dry *in vacuo* overnight. Anhydrous CH_2_Cl_2_ (5.00 mL) was added, followed by 0.100 g of activated powdered 3 Å molecular sieves, and the reaction was cooled to −78 °C under an atmosphere of N_2_. Next, *N*-iodosuccinimide (33.7 mg, 0.12 mmol) was added, followed by triflic acid (10.0 μL, 0.12 mmol), and the reaction was stirred for 3 h. The mixture was filtered, diluted with CH_2_Cl_2_ (10 mL), and washed with 10% Na_2_S_2_O_3_ (2 × 10 mL) and saturated NaHCO_3_ (10 mL). The organic layer was then dried with Na_2_SO_4_, filtered, and purified by flash chromatography (eluent: EtOAc/hexane) to yield **2** as a clear oil (42.0 mg, 0.093 mmol, 84%). *R*_f_ = 0.55 (EtOAc/hexane, 1/1) [α]D^25^ 108.8 (*c* 2.3, CHCl_3_) ^1^H NMR (500 MHz, CDCl_3_): δ 7.35–7.25 (m, 5H, Bn), 5.28 (s, 1H, H2), 4.87 (d, 1H, *J*_A,B_ = 11.1 Hz, CHA, OBn), 4.84 (br. s, 1H, NH), 4.70 (s, 1H, H1), 4.61 (d, 1H, *J*_B,A_ = 10.9 Hz, CHB, OBn), 3.72–3.65 (m, 2H, H5, CHA, OCH_2_CH_2_NH), 3.62 (dd, 1H, *J*_3,2_ = 3.4 Hz, *J*_3,4_ = 9.3 Hz, H3), 3.49–3.44 (m, 1H, CHA, OCH_2_CH_2_NH), 3.42 (s, 3H, OCH_3_), 3.36–3.30 (m, 1H, CHB, OCH_2_CH_2_NH), 3.35 (dd, 1H, *J*_4,5_ = *J*_4,3_ = 9.4 Hz, H4), 3.30–3.21 (m, 1H, CHB, OCH_2_CH_2_NH), 2.14 (s, 3H, Ac), 1.44 (s, 9H, CH_3_, Boc), 1.30 (d, 3H, *J*_6,5_ = 6.3 Hz, H6) ^13^C NMR (125 MHz, CDCl_3_): δ 170.5 (C=O), 155.9 (C=O, Boc), 138.6, 128.5 (2C), 128.0 (2C), 127.8 (Bn), 97.8 (C1), 80.1 (C4), 79.9 (C3), 79.5 (C(CH_3_)_3_, Boc) 75.4 (CH_2_, Bn), 68.6 (C2), 67.8 (C5), 67.3 (OCH_2_CH_2_NH), 57.6 (OCH_3_), 40.3 (OCH_2_CH_2_NH), 28.5 (CH_3_, Boc), 21.1 (Ac), 18.0 (C6). *J*_C1_,_H1_ = 171 Hz, ESI-MS: *m*/*z* calcd for C_23_H_35_NO_8_H [M + H]^+^, 454.2435; found, 454.2435.

### 2-*O*-Acetyl-1-*O*-[(*N*-ethyl)-*tert*-butoxycarbonyl]-3-*O*-methyl-α-d-rhamnopyranoside (**3**)

Pd(OH)_2_ (1.00 g, 7.00 mmol) was added to MeOH (20 mL), and the solution was preactivated with H_2_ for 30 min. Next, **2** (1.37 g, 3.02 mmol) was added and the solution was stirred under a continuous flow of H_2_ for 3 h at RT. The reaction was then filtered through Celite and evaporated to afford **3** (1.07 g, 2.94 mmol, 98%) as a clear oil. *R*_f_ = 0.35 (EtOAc/hexane, 1/1) [α]D^25^ 9.6 (*c* 0.28, CHCl_3_) ^1^H NMR (500 MHz, CDCl_3_/CD_3_OD): δ 5.29 (dd, 1H, *J*_2,1_ = 1.6 Hz, *J*_2,3_ = 2.7 Hz, H2), 4.78 (br. s, 1H, NH), 4.74 (d, 1H, *J*_1,2_ = 1.2 Hz, H1), 3.76–3.65 (m, 2H, H5, CHA, OCH_2_CH_2_NH), 3.54–3.48 (m, 2H, H4, CHB, OCH_2_CH_2_NH), 3.47 (dd, 1H, *J*_3,2_ = 3.1 Hz, *J*_3,4_ = 9.5 Hz, H3), 3.41 (s, 3H, OCH_3_), 3.38–3.36 (m, 2H, CHA, CHB, OCH_2_CH_2_NH), 2.34 (br. s, 1H, OH), 2.11 (s, 3H, Ac), 1.46 (s, 9H, CH_3_, Boc), 1.33 (d, 3H, *J*_6,5_ = 6.2 Hz, H6) ^1^H NMR (125 MHz, CDCl_3_/CD_3_OD): δ 170.4 (C=O), 155.9 (C=O, Boc), 98.0 (C1), 79.5 (C(CH_3_)_3_, Boc), 79.4 (C3), 71.6 (C4), 68.3 (C5), 67.5 (C2), 67.2 (OCH_2_CH_2_NH), 57.3 (OCH_3_), 40.3 (OCH_2_CH_2_NH), 28.4 (CH_3_, Boc), 20.9 (Ac), 17.7 (C6). *J*_C1_,_H1_ = 171 Hz, ESI-MS: *m*/*z* calcd for C_16_H_29_NO_8_H [M + H]^+^, 364.1966; found, 364.1963.

### 2-*O*-Acetyl-4-*O*-benzyl-3-*O*-methyl-α-d-rhamnopyranoside-(1 → 4)-2-*O*-acetyl-1-*O*-[(*N*-ethyl)-*tert*-butoxycarbonyl]-3-*O*-methyl-α-d-rhamnopyranoside (**4** di), 2-*O*-Acetyl-4-*O*-benzyl-3-*O*-methyl-α-d-rhamnopyranoside-(1 → 4)-2-*O*-acetyl-3-*O*-methyl-α-d-rhamnopyranoside-(1 → 4)-2-*O*-acetyl-1-*O*-[(*N*-ethyl)-*tert*-butoxycarbonyl]-3-*O*-methyl-α-d-rhamnopyranoside (**4** tri), 2-*O*-Acetyl-4-*O*-benzyl-3-*O*-methyl-α-d-rhamnopyranoside-(1 → 4)-2-*O*-acetyl-3-*O*-methyl-α-d-rhamnopyranoside-(1 → 4)-2-*O*-acetyl-3-*O*-methyl-α-d-rhamnopyranoside-(1 → 4)-2-*O*-acetyl-1-*O*-[(*N*-ethyl)-*tert*-butoxycarbonyl]-3-*O*-methyl-α-d-rhamnopyranoside (**4** tetra), and 2-*O*-Acetyl-4-*O*-benzyl-3-*O*-methyl-α-d-rhamnopyranoside-(1 → 4)-2-*O*-acetyl-3-*O*-methyl-α-d-rhamnopyranoside-(1 → 4)-2-*O*-acetyl-3-*O*-methyl-α-d-rhamnopyranoside-(1 → 4)-2-*O*-acetyl-3-*O*-methyl-α-d-rhamnopyranoside-(1 → 4)-2-*O*-acetyl-1-*O*-[(*N*-ethyl)-*tert*-butoxycarbonyl]-3-*O*-methyl-α-d-rhamnopyranoside (**4** penta)

Same procedures as **2**. See the Supporting Information for experimental procedures and characterization data for these compounds.

### 2-*O*-Acetyl-3-*O*-methyl-α-d-rhamnopyranoside-(1 → 4)-2-*O*-acetyl-1-*O*-[(*N*-ethyl)-*tert*-butoxycarbonyl]-3-*O*-methyl-α-d-rhamnopyranoside (**5** di), 2-*O*-Acetyl-3-*O*-methyl-α-d-rhamnopyranoside-(1 → 4)-2-*O*-acetyl-3-*O*-methyl-α-d-rhamnopyranoside-(1 → 4)-2-*O*-acetyl-1-*O*-[(*N*-ethyl)-*tert*-butoxycarbonyl]-3-*O*-methyl-α-d-rhamnopyranoside (**5** tri), 2-*O*-Acetyl-3-*O*-methyl-α-d-rhamnopyranoside-(1 → 4)-2-*O*-acetyl-3-*O*-methyl-α-d-rhamnopyranoside-(1 → 4)-2-*O*-Acetyl-3-*O*-methyl-α-d-rhamnopyranoside-(1 → 4)-2-*O*-acetyl-1-*O*-[(*N*-ethyl)-*tert*-butoxycarbonyl]-3-*O*-methyl-α-d-rhamnopyranoside (**5** tetra), and 2-*O*-Acetyl-3-*O*-methyl-α-d-rhamnopyranoside-(1 → 4)-2-*O*-acetyl-3-*O*-methyl-α-d-rhamnopyranoside-(1 → 4)-2-*O*-acetyl-3-*O*-methyl-α-d-rhamnopyranoside-(1 → 4)-2-*O*-acetyl-3-*O*-methyl-α-d-rhamnopyranoside-(1 → 4)-2-*O*-acetyl-1-*O*-[(*N*-ethyl)-*tert*-butoxycarbonyl]-3-*O*-methyl-α-d-rhamnopyranoside (**5** penta)

Same procedures as **3**. See the Supporting Information for experimental procedures and characterization data for these compounds.

### 3-*O*-Methyl-α-d-rhamnopyranoside-(1 → 4)-3-*O*-methyl-α-d-rhamnopyranoside-(1 → 4)-1-*O*-(2-aminoethyl)-3-*O*-methyl-α-d-rhamnopyranoside (**6** tri)

**5 tri** (755.0 mg, 0.98 mmol) was dissolved in MeOH (30.0 mL), and Na(s) (15.0 mg, 0.65 mmol) was added. The reaction mixture was stirred at RT for 16 h, neutralized with Dowex H^+^, filtered, and evaporated. The crude product was then dissolved in CH_2_Cl_2_ (100 mL), trifluoroacetic acid was added (5.8 mL), and the solution was stirred at RT for 10 min and evaporated under reduced pressure to obtain **6 tri** (532.4 mg, 0.98 mmol, 100%) as a beige powder. [α]D^25^ 1.5 (*c* 0.52, CH_3_OH) ^1^H NMR (600 MHz, CD_3_OD): δ 5.13 (s, 2H, H1′, H1″), 4.80 (s, 1H, H1), 4.12 (s, 1H, H2), 4.09 (s, 1H, H2′), 4.07 (s, 1H, H2″), 3.96–3.90 (m, 1H, CHA, OCH_2_CH_2_NH), 3.80–3.74 (m, 1H, H5′), 3.74–3.67 (m, 2H, H5, H5″), 3.67–3.57 (m, 3H, H4, H4′, CHB, OCH_2_CH_2_NH), 3.54 (dd, 1H, *J*_3,2_ = 2.0 Hz, *J*_3,4_ = 9.1 Hz, H3), 3.52–3.45 (m, 1H, H4″), 3.46 (s, 3H, OCH_3_), 3.44 (s, 3H, OCH_3_), 3.42 (s, 3H, OCH_3_), 3.41–3.35 (m, 1H, H3′), 3.27 (dd, 1H, *J*_3″,2″_ = 2.3 Hz, *J*_3″,4″_ = 9.5 Hz, H3″), 3.25–3.15 (m, 2H, CHA, CHB, OCH_2_CH_2_NH), 1.32 (d, 3H, *J*_6,5_ = 6.0 Hz, H6), 1.30 (d, 3H *J*_6,5_ = 6.1 Hz, H6′), 1.26 (d, 3H, *J*_6″,5″_ = 6.2 Hz, H6″) ^13^C NMR (150 MHz, CD_3_OD): δ 103.2 (C1′), 103.1 (C1″), 101.7 (C1), 83.1 (C3′), 82.8 (C3), 82.0 (C3″), 79.5 (C4), 79.3 (C4′), 72.6 (C4″), 70.5 (C5″), 69.1 (C5′), 68.7 (C5), 68.3 (C2′), 68.0 (C2″), 67.5 (C2), 64.6 (OCH_2_CH_2_NH), 57.3 (OCH_3_), 56.7 (OCH_3_), 56.6 (OCH_3_), 40.5 (OCH_2_CH_2_NH), 18.7 (C6), 18.6 (C6′), 17.9 (C6″). *J*_C1_,_H1_ = 171 Hz, ESI-MS: *m*/*z* calcd for C_23_H_43_O_13_NH [M + H]^+^, 542.2807; found, 542.2804.

### 3-*O*-Methyl-α-d-rhamnopyranoside-(1 → 4)-3-*O*-methyl-α-d-rhamnopyranoside-(1 → 4)-3-*O*-methyl-α-d-rhamnopyranoside-(1 → 4)-1-*O*-(2-aminoethyl)-3-*O*-methyl-α-d-rhamnopyranoside (**6** tetra)

The same procedure as **6** tri. **5** tetra (390.0 mg, 0.40 mmol), MeOH (30.0 mL), and Na(s) (10.0 mg, 0.44 mmol) were mixed, followed by CH_2_Cl_2_ (50 mL) and trifluoroacetic acid (2.7 mL). **6** tetra (282.1 mg, 0.40 mmol, 100%) was isolated as a beige powder. [α]D^25^ 1.1 (*c* 0.13, CH_3_OH) ^1^H NMR (600 MHz, CD_3_OD): δ 5.14 (m, 3H, H1′, H1″, H1‴), 4.81 (s, 1H, H1), 4.13 (s, 1H, H2), 4.09 (s, 3H, H2′, H2″, H2‴), 3.96–3.91 (m, 1H, CHA, OCH_2_CH_2_NH), 3.82–3.75 (m, 2H, H5′, H5″), 3.75–3.67 (m, 2H, H5, H5‴), 3.67–3.57 (m, 4H, H4, H4′, H4″, CHB, OCH_2_CH_2_NH), 3.57–3.52 (m, 1H, H3), 3.51–3.42 (m, 13H, 4x OCH_3_, H4‴), 3.42–3.36 (m, 2H, H3′, H3″), 3.31–3.26 (m, 1H, H3‴), 3.25–3.15 (m, 2H, CHA, CHB, OCH_2_CH_2_NH), 1.34 (d, 3H, *J*_6,5_ = 6.0 Hz, H6), 1.32–1.28 (m, 6H, H6′, H6″), 1.27 (d, 3H, *J*_6‴,5‴_ = 6.1 Hz, H6‴) ^13^C NMR (150 MHz, CD_3_OD): δ 103.2 (C1‴), 103.1 (C1″), 103.0 (C1′), 101.7 (C1), 83.1 (C3″), 83.0 (C3′), 82.8 (C3), 82.0 (C3‴), 79.4 (C4″), 79.3 (C4), 79.2 (C4′), 72.6 (C4‴), 70.5 (C5‴), 69.1 (2C, C5′, C5″), 68.7 (C5), 68.4 (C2′), 68.0 (C2″), 67.9 (C2‴), 67.5 (C2), 64.6 (OCH_2_CH_2_NH), 57.3 (OCH_3_), 56.7 (OCH_3_), 56.6 (2C, OCH_3_), 40.4 (OCH_2_CH_2_NH), 18.7 (C6), 18.5 (2C, C6′, C6″), 17.9 (C6‴). *J*_C1_,_H1_ = 170 Hz, ESI-MS: *m*/*z* calcd for C_30_H_55_O_17_NH [M + H]^+^, 702.3543; found, 702.3532.

### 3-*O*-Methyl-α-d-rhamnopyranoside-(1 → 4)-3-*O*-methyl-α-d-rhamnopyranoside-(1 → 4)-3-*O*-methyl-α-d-rhamnopyranoside-(1 → 4)-3-*O*-methyl-α-d-rhamnopyranoside-(1 → 4)-1-*O*-(2-aminoethyl)-3-*O*-methyl-α-d-rhamnopyranoside (**6** penta)

The same procedure as **6** tri. **5** penta (500.0 mg, 0.47 mmol), MeOH (30.0 mL), and Na(s) (10.0 mg, 0.44 mmol) were mixed, followed by CH_2_Cl_2_ (50 mL) and trifluoroacetic acid (2.7 mL). **6** penta (402.0 mg, 0.47 mmol, 100%) was isolated as a beige powder. [α]D^25^ 1.0 (*c* 0.26, CH_3_OH) ^1^H NMR (600 MHz, CD_3_OD): δ 5.15 (s, 4H, H1′, H1″, H1‴, H1‴′), 4.81 (d, 1H, *J*_1,2_ = 1.4 Hz, H1), 4.14–4.12 (m, 1H, H2), 4.12–4.08 (m, 4H, H2′, H2″, H2‴, H2‴′), 3.96–3.91 (m, 1H, CHA, OCH_2_CH_2_NH), 3.81–3.74 (m, 3H, H5′, H5″, H5‴), 3.75–3.67 (m, 2H, H5, H5‴′), 3.67–3.58 (5H, H4, H4′, H4″, H4‴, CHB, OCH_2_CH_2_NH), 3.55 (dd, 1H, *J*_3,2_ = 3.1 Hz, *J*_3,4_ = 9.1 Hz, H3), 3.50–3.46 (m, 4H, H4‴′, OCH_3_), 3.46 (s, 3H, OCH_3_), 3.45 (s, 3H, OCH_3_), 3.44 (s, 3H, OCH_3_), 3.42 (s, 3H, OCH_3_), 3.42–3.38 (m, 3H, H3′, H3″, H3‴), 3.30 (dd, 1H, *J*_3‴′_,_2‴′_ = 3.1 Hz, *J*_3‴′,4‴′_ = 9.4 Hz, H3‴′), 3.26–3.15 (m, 2H, CHA, CHB, OCH_2_CH_2_NH), 1.34 (d, 3H, *J*_6,5_ = 6.2 Hz, H6), 1.32–1.28 (m, 9H, H6′, H6″, H6‴), 1.27 (d, 3H, *J*_6‴′,5‴′_ = 6.2 Hz, H6‴′) ^13^C NMR (150 MHz, CD_3_OD): δ 103.2 (C1‴′), 103.1 (C1‴), 103.0 (2C, C1″, C1′), 101.7 (C1), 83.1 (3C, C3′, C3″, C3‴), 82.8 (C3), 82.0 (C3‴′), 79.4 (C4‴), 79.3 (C4), 79.2 (2C, C4′, C4″), 72.6 (C4‴′), 70.5 (C5‴′), 69.1 (3C, C5′, C5″, C5‴), 68.7 (C5), 68.4 (C2′), 67.9 (3C, C2″, C2‴, C2‴′), 67.5 (C2), 64.6 (OCH_2_CH_2_NH), 57.3 (OCH_3_), 56.7 (OCH_3_), 56.6 (3C, OCH_3_), 40.4 (OCH_2_CH_2_NH), 18.7 (C6), 18.6 (3C, C6′, C6″, C6‴), 17.9 (C6‴′). *J*_C1_,_H1_ = 170 Hz, ESI-MS: *m*/*z* calcd for C_37_H_67_O_21_NH [M + H]^+^, 862.4278; found, 862.4265.

### 3-*O*-Methyl-α-d-rhamnopyranoside-(1 → 4)-3-*O*-methyl-α-d-rhamnopyranoside-(1 → 4)-1-*O*-[(*N*-ethyl)-5,5-dimethoxypentanamide]-3-*O*-methyl-α-d-rhamnopyranoside (**1** tri)

2,5-Dioxopyrrolidin-1-yl 5,5-dimethoxypentanoate (239.0 mg, 0.92 mmol) was dissolved in anhydrous DMF (9.2 mL) and **6** tri (50.0 mg, 92.3 μmol) was added to this solution. The reaction mixture was stirred for 16 h at RT and pushed to completion by the addition of 1 drop of Et_3_N. The sample was evaporated under reduced pressure and coevaporated with toluene (5 × 10 mL). The crude sample was then suspended in H_2_O (2 mL) and extracted with CHCl_3_ (5 × 1 mL). The aqueous layer was collected, evaporated, and purified by HPLC (C-18, H_2_O/MeOH) to generate **1** tri (7.4 mg, 10.8 μmol, 12%) as a white powder. ^1^H NMR (600 MHz, CD_3_OD): δ 5.16–5.13 (m, 2H, H1′, H1″), 4.75 (d, 1H, *J*_1,2_ = 1.4 Hz, H1), 4.41 (t, 0.73H, CH(OCH_3_)_2_), 4.13–4.08 (m, 2H, H2′, H2″), 4.07 (dd, 1H, *J*_2,1_ = 1.9 Hz, *J*_2,3_ = 3.0 Hz, H2), 3.84–3.71 (m, 3.5H, H5′, H5″, CHA, OCH_2_CH_2_NH), 3.71–3.65 (m, 1H, H5), 3.64–3.55 (m, 2.8H, H4, H4′), 3.55–3.37 (m, 17H, H3, H3′, H4″, CHB, OCH_2_CH_2_NH, CHA, CHB, OCH_2_CH_2_NH, 3x OCH_3_), 3.34 (m, 4H, 2x OCH_3_), 3.29 (dd, 1H, *J*_3″,2″_ = 3.2 Hz, *J*_3″,4″_ = 9.4 Hz, H3″), 2.26 (t, 1.6H, COCH_2_), 1.73–1.66 (m, 1.6H, COCH_2_CH_2_), 1.66–1.60 (m, 1.66H, COCH_2_,CH_2_CH_2_), 1.31 (d, 3H, *J*_6,5_ = 6.1 Hz, H6), 1.30 (d, 3H, *J*_6′,5′_ = 6.2 Hz, H6′), 1.27 (d, 3H, *J*_6″,5″_ = 6.2 Hz, H6″) ^13^C NMR (150 MHz, CD_3_OD): δ 176.1 (CO), 105.8 (CH(OCH_3_)_2_), 103.2 (C1′), 103.1 (C1″), 101.3 (C1), 83.1 (C3′), 83.0 (C3), 82.0 (C3″), 79.7 (C4), 79.2 (C4′), 72.6 (C4″), 70.5 (C5″), 69.0 (C5′), 68.4 (C2″), 68.3 (C5), 68.0 (C2′), 67.6 (C2), 67.0 (OCH_2_CH_2_NH), 57.3 (OCH_3_), 56.7 (2x OCH_3_), 53.5 (OCH_3_), 53.4 (OCH_3_), 40.2 (OCH_2_CH_2_NH), 36.6 (COCH_2_), 33.1 (COCH_2_CH_2_CH_2_), 22.0 (COCH_2_CH_2_), 18.7 (C6), 18.6 (C6′), 17.8 (C6″). *J*_C1_,_H1_ = 172 Hz. ESI-MS: *m*/*z* calcd for C_30_H_55_O_16_N [M + Na]^+^, 708.3413; found, 708.3412.

### 3-*O*-Methyl-α-d-rhamnopyranoside-(1 → 4)-3-*O*-methyl-α-d-rhamnopyranoside-(1 → 4)-3-*O*-methyl-α-d-rhamnopyranoside-(1 → 4)-1-*O*-[(*N*-ethyl)-5,5-dimethoxypentanamide]-3-*O*-methyl-α-d-rhamnopyranoside (**1** tetra)

The same procedure as for **1** tri. 2,5-Dioxopyrrolidin-1-yl 5,5-dimethoxypentanoate (184.7 mg, 0.71 mmol), anhydrous DMF (7.1 mL), and **6** tetra (50.0 mg, 71.2 μmol) were mixed. **1** tetra (11.0 mg, 13.0 μmol, 18%) was isolated as a white powder. ^1^H NMR (600 MHz, CD_3_OD): δ 5.16–5.13 (m, 3H, H1′, H1″, H1‴), 4.75 (d, 1H, *J*_1,2_ = 1.6 Hz, H1), 4.41 (t, 1H, CH(OCH_3_)_2_), 4.13–4.09 (m, 3H, H2′, H2″, H2‴), 4.07 (dd, 1H, *J*_2,1_ = 1.9 Hz Hz, *J*_2,3_ = 3.0 Hz, H2), 3.82–3.71 (m, 4H, H5′, H5″, H5‴, CHA, OCH_2_CH_2_NH), 3.71–3.65 (m, 1H, H5), 3.64–3.56 (m, 3H, H4, H4′, H4″), 3.54–3.47 (m, 6H, H3, H4‴, OCH_3_, CHB, OCH_2_CH_2_NH), 3.47–3.36 (m, 13H, H3′, H3″, 3x OCH_3_, CHA, CHB, OCH_2_CH_2_NH), 3.35–3.32 (m, 6H, 2x OCH_3_), 3.31 (dd, 1H, *J*_3‴,2‴_ = 3.1 Hz, *J*_3‴,4‴_ = 9.4 Hz, H3‴), 2.26 (t, 2H, COCH_2_), 1.72–1.66 (m, 2H, COCH_2_CH_2_), 1.66–1.61 (m, 2H, COCH_2_CH_2_CH_2_), 1.32 (d, 1H, *J*_6,5_ = 6.2 Hz, H6), 1.30 (d, 1H, *J*_6′,5′_ = 6.5 Hz, H6′), 1.29 (d, 1H, *J*_6″,5″_ = 6.4 Hz, H6″), 1.27 (d, 1H, *J*_6‴,5‴_ = 6.2 Hz, H6‴) ^13^C NMR (150 MHz, CD_3_OD): δ 176.0 (CO), 105.8 (CH(OCH_3_)_2_), 103.2 (C1′), 103.1 (C1″), 103.0 (C1‴), 101.3 (C1), 83.1 (C3′), 83.0 (C3″), 82.9 (C3), 82.0 (C3‴), 79.8 (C4), 79.4 (C4″), 79.2 (C4′), 72.6 (C4‴), 70.5 (C5‴), 69.1 (C5′), 69.0 (C5″), 68.4 (C2‴), 68.3 (C5), 68.0 (2C, C2′, C2″), 67.7 (C2), 67.0 (OCH_2_CH_2_NH), 57.3 (OCH_3_), 56.7 (3x OCH_3_), 53.5 (OCH_3_), 53.4 (OCH_3_), 40.2 (OCH_2_CH_2_NH), 36.6 (COCH_2_), 33.1 (COCH_2_CH_2_CH_2_), 22.0 (COCH_2_CH_2_), 18.7 (C6), 18.6 (2C, C6′, C6″), 17.9 (C6‴). *J*_C1_,_H1_ = 171 Hz.. ESI-MS: *m*/*z* calcd for C_37_H_67_O_20_N [M + Na]^+^, 868.4149; found, 868.4144.

### 3-*O*-Methyl-α-d-rhamnopyranoside-(1 → 4)-3-*O*-methyl-α-d-rhamnopyranoside-(1 → 4)-3-*O*-methyl-α-d-rhamnopyranoside-(1 → 4)-3-*O*-methyl-α-d-rhamnopyranoside-(1 → 4)-1-*O*-[(*N*-ethyl)-5,5-dimethoxypentanamide]-3-*O*-methyl-α-d-rhamnopyranoside (**1** penta)

The same procedure as for **1** tri. 2,5-Dioxopyrrolidin-1-yl 5,5-dimethoxypentanoate (150.0 mg, 0.58 mmol), anhydrous DMF (5.8 mL), and **6** penta (50.0 mg, 58.0 μmol) were mixed. **1** penta (20.3 mg, 20.2 μmol, 35%) was isolated as a white powder. ^1^H NMR (600 MHz, CD_3_OD): δ 5.17–5.15 (m, 3H, H1″, H1‴, H1‴′), 5.14 (d, 1H, *J*_1′,2′_ = Hz, H1′), 4.75 (d, 1H, *J*_1,2_ = 1.7 Hz, H1), 4.41 (t, 1H, CH(OCH_3_)_2_), 4.13–4.10 (m, 4H, H2′, H2″, H2‴, H2‴′), 4.07 (dd, 1H, *J*_2,1_ = 2.0 Hz, *J*_2,3_ = 3.0 Hz, H2), 3.82–3.71 (m, 5H, H5′, H5″, H5‴, H5‴′, CHA, OCH_2_CH_2_NH), 3.71–3.65 (m, 1H, H5), 3.65–3.56 (m, 4H, H4, H4′, H4″, H4‴), 3.52–3.38 (m, 23H, H3, H3′, H3″, H3‴, H4‴′, CHB, OCH_2_CH_2_NH, CHA, CHB, OCH_2_CH_2_NH, 5× OCH_3_), 3.36–3.33 (m, 6H, 2× OCH_3_), 3.31 (dd, 1H, *J*_3‴′,2‴′_ = 3.1 Hz, *J*_3‴′,4‴′_ = 9.4 Hz, H3‴′), 2.26 (t, 2H, COCH_2_), 1.73–1.66 (m, 2H, COCH_2_CH_2_), 1.66–1.61 (m, 2H, COCH_2_CH_2_CH_2_), 1.34–1.28 (m, 12H, H6, H6′, H6″, H6‴), 1.27 (d, *J*_6‴′,5‴′_ = 6.2 Hz, H6‴′) ^13^C NMR (150 MHz, CD_3_OD): δ 176.0 (CO), 105.8 (CH(OCH_3_)_2_), 103.2 (2C, C1′, C1″), 103.1 (C1‴, C1‴′), 101.3 (C1), 83.1 (2C, C3′, C3‴), 83.0 (C3), 82.9 (C3″), 82.0 (C3‴′), 79.8 (C4), 79.5 (C4‴), 79.4 (C4″), 79.2 (C4′), 72.7 (C4‴′), 70.5 (C5‴′), 69.1 (C5′), 69.0 (2C, C5″, C5‴), 68.3 (2C, C5, C2‴′), 68.0 (3C, C2′, C2″, C2‴), 67.6 (C2), 67.1 (OCH_2_CH_2_NH), 57.3 (OCH_3_), 56.7 (4x OCH_3_), 53.5 (OCH_3_), 53.4 (OCH_3_), 40.2 (OCH_2_CH_2_NH), 36.6 (COCH_2_), 33.1 (COCH_2_CH_2_CH_2_), 22.0 (COCH_2_CH_2_), 18.7 (C6), 18.6 (3C, C6′, C6″, C6‴), 17.9 (C6‴′). *J*_C1_,_H1_ = 171 Hz.. ESI-MS: *m*/*z* calcd for C_44_H_79_O_24_N [M + Na]^+^, 1028.4884; found, 1028.4884.

### Inhibition ELISA

1B1 mAb previously identified as specific for the methyl rhamnan tip at a constant concentration of 10 μg/mL in PBS–Tween was incubated at a ratio of 1:1 with dilutions of *Pa* PAO1 BAA-47 (wt) lipopolysaccharide (LPS) (positive control for inhibition) and *Neisseria meningitidis* (*Nm*) *galE/lpt3* LPS (negative control for inhibition) and the synthetic oligosaccharides with handle (**1** tri, **1** tetra, **1** penta). The final concentration of mAb 1B1 was 5 μg/mL. *Pa* and *Nm* LPS and the synthetic oligosaccharides were titrated starting at a concentration of 3 mg/mL (final 1.5 mg/mL) and diluted 2-fold, 12 times in PBS–Tween. This mixture was incubated together for 1 h at RT before adding to *Pa* wt LPS-coated ELISA plates for 1 h at RT. ELISA was then performed as described previously^[^^5^^b]^.

#### Conjugation of **1** tri, **1** tetra, and **1** penta to Activated CRM and Activated BSA

BSA and CRM_197_ were activated as described prior^18^ and within the Supporting Information. Then, **1** tri, **1** tetra, and 1 penta (3 mg/mL) were dissolved in 50% acetic acid. After 7 h at 37 °C, the reaction mixture was cooled and then lyophilized, which led to the deprotection of the acetal to the aldehyde for further conjugation with the aminooxy-proteins. Deprotected **1** tri, **1** tetra, or **1** penta (3 mg) were then separately dissolved in sodium phosphate buffer (50 μL, 200 mM, pH 6) and separately added to the aminooxy-proteins (CRM_197_-oxy, respectively 2.1, 1.7 and 1.5 mg). The amount of aminooxy-protein used was adjusted in each case to keep an approximate 8× molar excess of oligosaccharide per aminooxy on the protein. The product was isolated after 18 h at RT using an Amicon Ultra-10 30K MWCO filter against PBS (3×) and stored at 4 °C. The carbohydrate/protein ratio was determined by MALDI-MS (Table S1).

#### Immunization of Mice and Rabbits with Conjugates

Female BALB/c mice, 6 to 8 weeks old, were immunized intraperitoneally (3×). Each mouse received the same amount of conjugate, as well as SIGMA adjuvant, and PBS buffer at each time point. The mice were primed on day 0 and received boosters on days 21 and 42, and blood samples were taken on day 0, day 35, and day 56. Each mouse in group MRha3V received 3 μg of the trisaccharide conjugate and 28 μg of CRM, along with 50% v/v SIGMA adjuvant, and PBS buffer totaling 100 μL, administered intraperitoneally. Each mouse in group MRha4V received 3 μg of the tetrasaccharide conjugate and 25.5 μg of CRM, along with 50% v/v SIGMA adjuvant, and PBS buffer totaling 100 μL, administered intraperitoneally. Finally, each mouse in group MRha5V received 3 μg of the pentasaccharide and 23 μg of CRM, along with 50% v/v SIGMA adjuvant, and PBS buffer totaling 100 μL, administered intraperitoneally. Blood samples were obtained by the submandibular vein collection method to yield approximately 100 μL of serum after blood separation.

Female New England white rabbits, 1.5–2.0 kg, were immunized three times subcutaneously at two separate sites for each immunization. Each rabbit received the same amount of oligosaccharide conjugate, as well as SIGMA adjuvant, and PBS buffer at each time point. The rabbits were primed on day 0 and received boosters on days 21 and 49, and blood samples were taken on day 0, day 35, and day 70. Each rabbit in group RRha3V received 20 μg of the trisaccharide conjugate and 188 μg of CRM_197_, along with 50% v/v SIGMA adjuvant, and PBS buffer totaling 500 μL, administered subcutaneously with 250 μL in each of 2 sites. Each rabbit in group RRha4V received 20 μg of the tetrasaccharide conjugate and 170 μg of CRM_197_, along with 50% v/v SIGMA adjuvant, and PBS buffer totaling 500 μL, administered subcutaneously with 250 μL in each of 2 sites. Each rabbit in group RRha5V received 20 μg of the pentasaccharide and 151.5 μg of CRM_197_, along with 50% v/v SIGMA adjuvant, and PBS buffer totaling 500 μL, administered subcutaneously with 250 μL in each of 2 sites. Blood samples were obtained by marginal ear vein collection method to yield approximately 500 μL of serum after blood separation.

### ELISA

ELISA experiments with mice and rabbit sera against BSA conjugates, LPS, and whole cells were performed as described previously.^11^

## Supporting Information Available

Additional experimental procedures, experimental data, NMR spectra, Maldi spectra, inhibition ELISA results, mice ELISA results, rabbit ELISA results from BSA conjugates, screening of bacterial clinical cell isolates with mice, and rabbit pre and post immune sera. (PDF)
