# Supplementary material for: Optimization of the Synthesis and Conjugation of the Methyl Rhamnan Tip of Pseudomonas aeruginosa A-Band Polysaccharide and Immunogenicity Evaluation for the Continued Development of a Potential Glycoconjugate Vaccine
Source: ACS Infect Dis. 2024 Mar 6;10(4):1361–9. doi: 10.1021/acsinfecdis.4c00049 (PMC11019553; doi:10.1021/acsinfecdis.4c00049)
Supplement: Supplementary file 1 — id4c00049_si_001.pdf [file id4c00049_si_001.pdf]

Optimization of the synthesis and conjugation of the methyl rhamnan tip of *Pseudomonas aeruginosa* A-band polysaccharide and immunogenicity evaluation for the continued development of a potential glycoconjugate vaccine

Mohammad P. Jamshidi,[a] Chantelle Cairns,[a] Nam Huan Khieu,[a] Kenneth Chan,[a] Frank St. Michael,[a] Andrew Cox,[a] Janelle Sauvageau[a,*]

[a]Vaccine and Emerging Infections Research, Human Health Therapeutics Research Centre, National Research Council, Ottawa, ON, K1A 0R6, Canada.

**corresponding author. Human Health Therapeutics, National Research Council of Canada, 100 Sussex Dr. Ottawa, K1N 5A2, Canada, Email address: Janelle.Sauvageau@nrc-cnrc.gc.ca*

Table of Contents

Table of Contents

Experimental Methods 2

Synthesis of oligosaccharides 2

Inhibition ELISA assay 8

Conjugation of oligosaccharide to activated CRM_{197} and activated BSA to prepare glycoconjugates 9

Screening of derived mice sera vs. BSA-conjugates and LPS: 9

Screening of derived mice and rabbit sera vs. killed whole cells 14

NMR Spectra 17

MALDI Spectra 36

References: 38

Experimental Methods

Synthesis of oligosaccharides

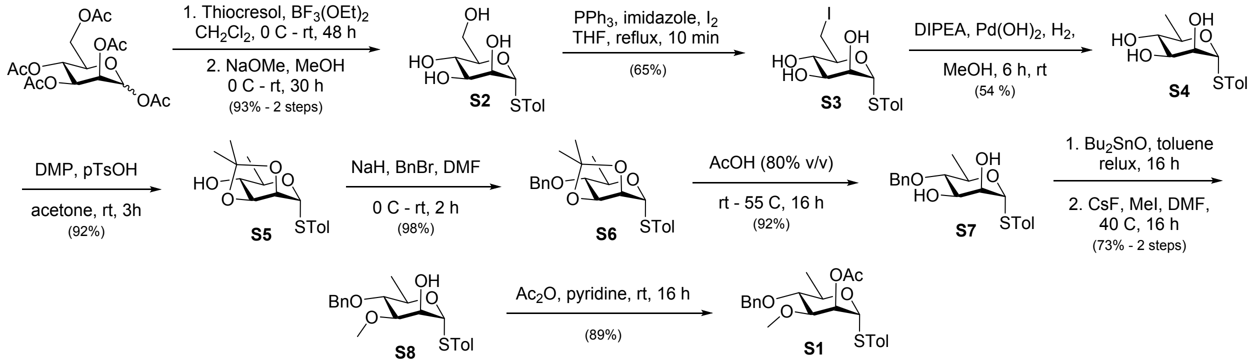

Scheme S1. Synthesis of advanced intermediate *p*-tolyl 4-*O*-benzyl-3-*O*-methyl-1-thio-α-ᴅ-rhamnopyranoside (S1)

p-Tolyl 2-*O*-Acetyl-4-*O*-benzyl-3-*O*-methyl-1-thio-α-ᴅ-rhamnopyranoside (**S1**)

Compound **S8** (10.85 g, 29.0 mmol) was dissolved in acetic anhydride (20 mL) and pyridine (20 mL) and the solution was stirred at RT for 16 h. The solution was then evaporated under reduced pressure and coevaporated with toluene (5 x 30 mL). The crude oil was then purified by flash chromatography (eluent: EtOAc/Hexane) to yield compound **S1** (10.77 g, 25.9 mmol, 89 %) as a clear oil. Spectral data agreed with literature values.[1]

p-Toly 1-thio-α-ᴅ-mannopyranoside (**S2**)

In a 2000 mL round bottom flask, acetylated mannose (142.5 g, 365.1 mmol) was dissolved in 800 mL of anhydrous CH_2Cl_2 and the solution was cooled to 0 °C under an atmosphere of nitrogen. Next, thiocresol (63.5 g, 511.0 mmol) was added, followed by boron trifluoride diethethyl etherate (63.1 mL, 511.0 mmol) and the solution was stirred at RT for 48 h at which point the TLC had shown completion. The reaction was then washed with water (2 x 200 mL) and $NaHCO_3$ (2 x 200 mL). The organic layer was isolated and dried with Na_2SO_4, filtered, and evaporated under reduced pressure. The crude product was then dissolved in 1000 mL of anhydrous MeOH, and cooled to 0 °C. Sodium metal (0.873 g, 36.4 mmol) was then added and the reaction was stirred at RT for 30 h before reaching completion. The solution was neutralized with Dowex H^+, filtered, and evaporated. The crude product was then dissolved in water (500 mL) and extracted with CH_2Cl_2 (3 x 150 mL). The water layer was then evaporated to afford **S2** as a light green powder (96.86 g, 338.2 mmol, 93 % over 2 steps). Spectral data agreed with literature values.[2]

p-Tolyl 6-iodo-1-thio-α-ᴅ-mannopyranoside (**S3**)

S2 (96.86 g, 338.2 mmol) was dissolved in anhydrous THF (600 mL) and the solution was refluxed under N_2. Next, triphenylphosphine (133.05 g, 507.3 mmol), imidazole (46.04 g, 676.4 mmol), and I_2 (128.76 g,

507.3 mmol) were slowly added sequentially. The reaction was complete after 10 minutes when the purple color of iodine persisted. The solution was then cooled to RT and concentrated under reduced pressure. The crude mixture was dissolved in ethyl acetate (600 mL) and washed with 10 % $Na_2S_2O_3$ (2 x 150 mL) and water (2 x 150 mL). The organic layer was then dried with Na_2SO_4, filtered, evaporated, and purified by flash chromatography (eluent: MeOH/CH_2Cl_2, 1/5) to yield **S3** as a white solid (87.37 g, 220.5 mmol, 65 %). Rf = 0.65 (MeOH/CH_2Cl_2, 1/9) $[\alpha]_D^{25}$ -2.1 (*c* 0.041, MeOH) ^1H NMR (400 MHz, CD_3OD): δ 7.47 (d, 2H, *J* = 8.1 Hz, Stol), 7.14 (d, 2H, *J* = 8.0 Hz, Stol), 5.33 (s, 1H, H1), 4.1 (s, 1H, H2), 3.93 (dd, 1H, $J_{4,3}$ = $J_{4,5}$ = 8.2 Hz, H4), 3.67-3.52 (m, 3H, H3, H5, H6), 3.30 (m, 4H, OH, H6), 2.32 (s, 3H, CH_3, Stol) ^{13}C NMR (100 MHz, CD_3OD): δ 138.9, 133.4 (2C), 132.2, 130.7 (2C, Stol), 91.0 (C1), 74.9 (C4), 73.8 (C2), 72.7 (C5), 72.6 (C3), 21.1 (CH_3, Stol), 6.0 (C6). $J_{C1,H1}$ = 169 Hz, ESI-MS: *m/z* Calcd for $C_{13}H_{17}O_4INaS$ $[M+Na]^+$, 418.9784; found, 418.9786.

p-Tolyl 1-thio-ᴅ-rhamnopyranoside (**S4**)

S3 (1.76 g, 4.44 mmol) was dissolved in MeOH (30 mL),Pd$(OH)_2$ (0.39 g, 2.76 mmol) was added and the solution kept under anH_2 atmosphere. Next, *N,N*-diisopropyl ethyl amine (1.8 mL, 10.3 mmol) was added and the solution was stirred under an H_2 atmosphere for 6 h. The solution was then filtered through a celite, evaporated, and purified by flash chromatography (eluent: MeOH/CH_2Cl_2, 1/5) to afford **S4** as a clear oil (0.65 g, 2.40 mmol, 54 %). Rf = 0.50 (MeOH/CH_2Cl_2, 1/9) $[\alpha]_D^{25}$ 0.91 (*c* 0.043, MeOH) ^1H NMR (400 MHz, CD_3OD): δ 7.35 (d, 2H, *J* = 7.7 Hz, Stol), 7.14 (d, 2H, *J* = 7.6 Hz, Stol), 5.29 (s, 1H, H1), 4.10-4.00 (m, 2H, H2, H5), 3.64 (dd, 1H, $J_{3,2}$ = 3.2 Hz, $J_{3,4}$ = 9.4 Hz, H3), 3.45 (dd, 1H, $J_{4,3}$ = $J_{4,5}$ = 9.5 Hz, H4), 3.31 (m, 3H, OH), 2.31 (s, 3H, CH_3, Stol), 1.26 (d, 3H, $J_{6,5}$ = 6.2 Hz, H6) ^{13}C NMR (100 MHz, CD_3OD): δ 138.8, 133.3 (2C), 132.2, 130.8 (2C, Stol), 90.6 (C1), 74.2 (C4), 73.8 (C2), 72.9 (C3), 70.9 (C5), 21.1 (CH_3, Stol), 17.8 (C6). $J_{C1,H1}$ = 170 Hz, ESI-MS: *m/z* Calcd for $C_{13}H_{18}O_4NaS$ $[M+Na]^+$, 293.0818; found, 293.0818.

p-Tolyl 2,3-*O*-Isopropylidene-1-thio-α-ᴅ-rhamnopyranoside (**S5**)

Compound **S4** (10.16 g, 37.6 mmol) was dissolved in 250 mL of anhydrous acetone. To this solution, 2,2-dimethoxypropane (8.29 mL, 67.6 mmol) and p-toluenesulfonic acid monohydrate (0.71 g, 0.376 mmol) were added and the reaction was stirred at RT for 3 h before reaching completion. The solution was evaporated under reduced pressure, dissolved in ethyl acetate (300 mL), and washed with saturated $NaHCO_3$ (2 x 100 mL). The combined organic layers were dried with Na_2SO_4, filtered, and purified by flash chromatography (eluent: EtOAc/Hexane) to afford compound **S5** (10.68 g, 34.4 mmol, 92%) as a white solid. Spectral data agreed with literature values.[3]

p-Tolyl 4-*O*-Benzyl-2,3-*O*-isopropylidene-1-thio-α-ᴅ-rhamnopyranoside (**S6**)

Compound **S5** (10.68 g. 34.4 mmol) was dissolved in anhydrous DMF (150 mL) and the solution was cooled to 0 ^oC. Next, NaH (2.06 g, 51.6 mmol) was added portion-wise and the solution was stirred for 30 mins. Benzyl bromide (4.91 mL. 41.3 mmol) was added dropwise and the solution was stirred at RT for 2 hours before reaching completion. The reaction was quenched by the addition of Et_3N (10 mL) and was then poured into a saturated, cold solution of NH_4Cl (400 mL). The solution was stirred at 0 ^oC for 1 hour, then filtered. The solids were collected and dried under high vacuum to yield **S6** (13.43 g, 33.5 mmol, 98%) as a beige solid. Spectral data agreed with literature values.[4]

p-Tolyl 4-*O*-Benzyl-1-thio-α-ᴅ-rhamnopyranoside (**S7**)

Compound **S6** (1.09 g, 2.72 mmol) was dissolved in an 80 % (v/v) solution of AcOH (10 mL) and stirred overnight at RT. The solution was then heated to 55 ^oC in an oil bath for 3 h to drive the reaction to completion. The solution was then evaporated under reduced pressure and coevaporated with toluene (4 x 30 mL) to afford **S7** (909 mg, 2.52 mmol, 92%) as a clear oil. Spectral data agreed with literature values.[4]

p-Tolyl 4-*O*-benzyl-3-*O*-methyl-1-thio-α-ᴅ-rhamnopyranoside (**S8**)

S7 (14.3 g, 39.7 mmol) was coevaporated with toluene (3 x 100 mL) and dried on high vacuum overnight. Anhydrous toluene (400 mL) was added and the solution was purged with N_2. Dibultyltin (IV) oxide (11.85 g, 47.6 mmol) was added and the reaction mixture was stirred under reflux for 16 h, cooled to RT, and evaporated under reduced pressure. The crude tin acetal was left on high vacuum for 5 h, dissolved in anhydrous DMF (793 mL) and purged with N_2. Cesium fluoride (9.00 g, 59.6 mmol) and iodomethane (28.15 mL, 396.6 mmol) were added and the reaction was stirred at 40 ^oC for 16 h under N_2. The mixture was cooled to RT and diluted with EtOAc (350 mL), washed with water (5 x 150 mL), saturated $NaHCO_3$ (150 mL), and brine (150 mL). The combined organic layers were then dried with Na_2SO_4, evaporated and purified by flash chromatography (eluent: EtOAc/hexane) to afford **S8** as a clear oil (10.87 g, 29.0 mmol, 73 %). Rf = 0.40 (EtOAc/Hexane, 3/7) $[\alpha]_D^{25}$ 8.2 (*c* 0.38, $CHCl_3$) ^1H NMR (400 MHz, CD_3Cl): δ 7.40-7.27 (m, 7H, Stol, Bn), 7.11 (d, 2H, J = 7.9 Hz, Stol), 5.47 (s, 1H, H1), 4.86 (d, 1H, $J_{A,B}$ = 10.9 Hz, CHA, OBn), 4.64 (d, 1H, $J_{B,A}$ = 11.0 Hz, CHB, OBn), 4.32-4.27 (m, 1H, H2), 4.25-4.15 (m, 1H, H5), 3.58 (dd, 1H, $J_{3,2}$ = 3.1 Hz, $J_{3,4}$ = 9.1 Hz, H3), 3.52 (s, 3H, OCH_3), 3.44 (dd, 1H, $J_{4,3} = J_{4,5}$ = 9.2 Hz, H4), 2.32 (s, 3H, CH_3, Stol), 1.30 (d, 3H, $J_{6,5}$ = 6.3 Hz, H6) ^{13}C NMR (100 MHz, CD_3Cl): δ 138.6, 137.8, 132.2 (2C), 130.4, 130.0 (2C), 128.6 (2C), 128.1 (2C), 127.9 (Stol, Bn), 87.6 (C1), 82.1 (C3), 80.2 (C4), 75.4 (CH_2, Bn), 69.5 (C2), 68.6 (C5), 57.6 (OCH_3), 21.2 (CH_3, Stol), 17.9 (C6). $J_{C1,H1}$ = 167 Hz, ESI-MS: *m/z* Calcd for $C_{21}H_{26}O_4NaS$ $[M+Na]^+$, 397.1444; found, 397.1444.

2-*O*-Acetyl-4-*O*-benzyl-3-*O*-methyl-α-ᴅ-rhamnopyranoside-(1→4)-2-*O*-acetyl-1-*O*-[(*N*-ethyl)-*tert*-butoxycarbonyl]-3-*O*-methyl-α-ᴅ-rhamnopyranoside (**4 di**)

Same procedure as **2**. See supporting information for experimental procedures for these compounds. **5 di** (1.07 g, 2.93 mmol), **S1** (1.47 g, 3.53 mmol), anhydrous CH_2Cl_2 (50 mL), 0.500 g of activated powdered 3 Å molecular sieves, *N*-iodosuccinimide (1.13 g, 4.11 mmol) and triflic acid (0.50 mL, 5.66 mmol). **7 di** (1.56 g, 2.38 mmol, 81 %) was isolated a clear oil. Rf = 0.65 (EtOAc/Hexane, 1/1) $[\alpha]_D^{25}$ 66.8 (*c* 1.0, $CHCl_3$) ^1H NMR (500 MHz, $CDCl_3$): δ 7.38-7.27 (m, 5H, Bn), 5.35 (dd, 1H, $J_{2',1'}$ = 1.2 Hz, $J_{2',3'}$ = 2.8 Hz, H2′), 5.28 (s, 1H, H2), 5.12 (d, 1H, $J_{1',2'}$ = 1.2 Hz, H1′), 4.89 (d, 1H, $J_{A,B}$ = 10.8 Hz, CHA, OBn), 4.82 (br. s, 1H, NH), 4.69 (d, 1H, $J_{1,2}$ = 1.2 Hz, H1), 4.61 (d, 1H $J_{B,A}$ = 10.8 Hz, CHB, OBn), 3.86-3.77 (m, 1H, H5′), 3.75-3.66 (m, 1H, CHA, $O\underline{CH_2}CH_2NH$), 3.66-3.55 (m, 2H, H5, H3′), 3.55-3.45 (m, 3H, H3, H4, CHB, $O\underline{CH_2}CH_2NH$), 3.44 (s, 3H, OCH_3), 3.39 (s, 3H, OCH_3), 3.39-3.33 (m, 2H, H4′, CHA, $OCH_2\underline{CH_2}NH$), 3.33-3.23 (m, 1H, CHB, $OCH_2\underline{CH_2}NH$), 2.13 (s, 3H, Ac), 2.10 (s, 3H, Ac), 1.46 (s, 9H, CH_3, Boc), 1.34-1.28 (m, 6H, H6, H6′) ^{13}C NMR (125 MHz, $CDCl_3$): δ 170.4, 170.2 (C=O), 155.9 (C=O, Boc), 138.6, 128.4 (2C), 128.0 (2C), 127.8 (Bn), 99.4 (C1′), 97.8 (C1), 80.1 (2C, C3, C4′), 79.9 (C3′), 79.6 ($\underline{C}(CH_3)_3$, Boc), 78.3 (C4), 75.4 (CH_2, Bn), 68.7 (C2′), 68.5 (C5′), 67.9 (C2), 67.3 ($O\underline{C}H_2CH_2NH$), 67.1 (C5), 57.6 (OCH_3), 57.4 (OCH_3), 40.3 ($OCH_2\underline{C}H_2NH$), 28.5 (CH_3, Boc), 21.2, 21.0 (2x Ac), 18.2 (C6′), 17.9 (C6). $J_{C1,H1}$ = 168 Hz, ESI-MS: *m/z* Calcd for $C_{32}H_{49}NNaO_{13}$ $[M+Na]^+$, 678.3096; found, 678.3092.

2-*O*-Acetyl-4-*O*-benzyl-3-*O*-methyl-α-D-rhamnopyranoside-(1→4)-2-*O*-acetyl-3-*O*-methyl-α-D-rhamnopyranoside-(1→4)-2-*O*-acetyl-1-*O*-[(*N*-ethyl)-*tert*-butoxycarbonyl]-3-*O*-methyl-α-D-rhamnopyranoside (**4 tri**)

Same procedure as **2**. **5 di** (1.46 g, 2.57 mmol), **S1** (1.29 g, 3.10 mmol), anhydrous CH_2Cl_2 (50 mL), 0.400 g of activated powdered 3 Å molecular sieves, *N*-iodosuccinimide (990.0 mg, 3.60 mmol) and triflic acid (0.40 mL, 4.52 mmol). **4 tri** (1.65 g, 1.92 mmol, 75 %) was isolated a clear oil. Rf = 0.45 (EtOAc/Hexane, 1/1) $[\alpha]_D^{25}$ 2.0 (*c* 0.46, $CHCl_3$) ^1H NMR (600 MHz, $CDCl_3$): δ 7.37-7.26 (m, 5H, Bn), 5.35 (s, 1H, H2''), 5.33 (s, 1H, H2'), 5.27 (s, 1H, H2), 5.10 (s, 1H, H1''), 5.09 (s, 1H, H1'), 4.88 (d, 1H, $J_{A,B}$ = 10.9 Hz, CHA, OBn), 4.83 (br. s, 1H, NH), 4.70 (s, 1H, H1), 4.61 (d, 1H, $J_{B,A}$ = 10.9 Hz, CHB, OBn), 3.85-3.71 (m, 1H, H5''), 3.77-3.65 (m, 3H, H5, H5', CHA, O<u>CH</u>$_2$CH$_2$NH), 3.59 (dd, 1H, $J_{3'',2''}$ = 3.0 Hz, $J_{3'',4''}$ = 9.3 Hz, H3''), 3.56-3.45 (m, 5H, H3, H3', H4, H4', CHB, O<u>CH$_2$</u>CH$_2$NH), 3.44 (s, 3H, OCH_3), 3.40 (s, 3H, OCH_3), 3.39-3.37 (m, 4H, OCH_3, CHA, OCH$_2$<u>CH$_2$</u>NH), 3.38-3.33 (m, 1H, H4''), 3.32-3.26 (m, 1H, CHB, OCH$_2$<u>CH$_2$</u>NH), 2.13 (s, 3H, Ac), 2.10 (s, 3H, Ac), 2.08 (s, 3H, Ac), 1.46 (s, 9H, CH_3, Boc), 1.31 (d, 3H, $J_{6'',5''}$ = 5.8 Hz, H6''), 1.30 (d, 3H, $J_{6,5}$ = 5.1 Hz, H6), 1.28 (d, 3H, $J_{6',5'}$ = 6.2 Hz, H6') ^{13}C NMR (150 MHz, $CDCl_3$): δ 170.4, 170.2, 170.1 (C=O), 155.9 (C=O, Boc), 138.6, 128.5 (2C), 128.1 (2C), 127.8 (Bn), 99.5 (2C, C1', C1''), 97.9 (C1), 80.1 (2C, C3, C4''), 80.0 (C3'), 79.9 (C3''), 79.7 (<u>C</u>(CH_3)$_3$, Boc), 78.7 (C4), 78.4 (C4'), 75.5 (CH_2, Bn), 68.7 (C2''), 68.5 (C5''), 68.0 (C2'), 67.9 (C2), 67.8 (C5'), 67.3 (O<u>C</u>H$_2$CH$_2$NH), 67.1 (C5), 57.6 (OCH_3), 57.5 (OCH_3), 57.3 (OCH_3), 40.4 (OCH$_2$<u>C</u>H$_2$NH), 28.5 (CH_3, Boc), 21.2, 21.1 (3x Ac), 18.3 (C6'), 18.1 (C6''), 17.9 (C6''). $J_{C1,H1}$ = 172 Hz, ESI-MS: *m/z* Calcd for $C_{41}H_{63}O_{18}NNa$ $[M+Na]^+$, 880.3937; found, 880.3943.

2-*O*-Acetyl-4-*O*-benzyl-3-*O*-methyl-α-D-rhamnopyranoside-(1→4)-2-*O*-acetyl-3-*O*-methyl-α-D-rhamnopyranoside-(1→4)-2-*O*-acetyl-3-*O*-methyl-α-D-rhamnopyranoside-(1→4)-2-*O*-acetyl-1-*O*-[(*N*-ethyl)-*tert*-butoxycarbonyl]-3-*O*-methyl-α-D-rhamnopyranoside (**4 tetra**)

Same procedure as **2**. **5 tri** (1.45 g, 1.89 mmol), **S1** (943.9 mg, 2.27 mmol), anhydrous CH_2Cl_2 (50 mL), 0.400 g of activated powdered 3 Å molecular sieves, *N*-iodosuccinimide (727.0 mg, 2.64 mmol) and triflic acid (0.35 mL, 3.96 mmol). **4 tetra** (1.49 g, 1.41 mmol, 74 %) was isolated a clear oil. Rf = 0.40 (EtOAc/Hexane, 1/1) $[\alpha]_D^{25}$ 1.7 (*c* 0.31, $CHCl_3$) ^1H NMR (600 MHz, $CDCl_3$): δ 7.37-7.26 (m, 5H, Bn), 5.35 (dd, 1H, $J_{2''',1'''}$ = 2.0 Hz, $J_{2''',3'''}$ = 3.1 Hz, H2'''), 5.35-5.31 (m, 2H, H2', H2''), 5.28 (s, 1H, H2), 5.12-5.10 (m, 2H, H1'', H1'''), 5.08 (s, 1H, H1'), 4.89 (d, 1H, $J_{A,B}$ = 10.9 Hz, CHA, OBn), 4.83 (br. s, 1H, NH), 4.70 (d, 1H, $J_{1,2}$ = 1.0 Hz, H1), 4.61 (d, 1H, $J_{B,A}$ = 10.9 Hz, CHB, OBn), 3.87-3.73 (m, 3H, H5', H5'', H5'''), 3.73-3.63 (m, 2H, H5, CHA, O<u>CH$_2$</u>CH$_2$NH), 3.60 (dd, 1H, $J_{3''',2'''}$ = 3.2 Hz, $J_{3''',4'''}$ = 9.3 Hz, H3'''), 3.57-3.46 (m, 7H, H3, H3', H3'', H4, H4', H4'', CHB, O<u>CH$_2$</u>CH$_2$NH), 3.44 (s, 3H, OCH_3), 3.42-3.32 (m, 11H, 3x OCH_3, C4''', CHA, OCH$_2$<u>CH$_2$</u>NH), 3.32-3.26 (m, 1H, CHB, OCH$_2$<u>CH$_2$</u>NH), 2.13 (s, 3H, Ac), 2.10 (s, 3H, Ac), 2.08 (s, 6H, 2x Ac), 1.46 (s, 9H, CH_3, Boc), 1.35-1.26 (m, 12H, H6, H6', H6'', H6''') ^{13}C NMR (150 MHz, $CDCl_3$): δ 170.1, 170.3, 170.2, 170.1 (C=O), 155.9 (C=O, Boc), 138.6, 128.5 (2C), 128.1 (2C), 127.9 (Bn), 99.6 (C1'), 99.5 (2C, C1'', C1'''), 97.9 (C1), 80.1 (3C, C3', C3'', C4'''), 80.0 (2C, C3, C3'''), 79.7 (<u>C</u>(CH_3)$_3$, Boc), 78.9 (C4), 78.4 (2C, C4', C4''), 75.5 (CH_2, Bn), 68.7 (C2'''), 68.5 (C5'''), 68.0 (2C, C2', C2''), 67.9 (C2), 67.8 (2C, C5', C5''), 67.3 (O<u>C</u>H$_2$CH$_2$NH), 67.1 (C5), 40.4 (OCH$_2$<u>C</u>H$_2$NH), 28.6 (CH_3, Boc), 21.3, 21.2 (3C, 4x Ac), 18.4 (C6), 18.2 (C6'), 18.1 (C6''), 18.0 (C6'''). $J_{C1,H1}$ = 172 Hz, ESI-MS: *m/z* Calcd for $C_{50}H_{77}NO_{23}H$ $[M+H]^+$, 1060.4959; found, 1060.4977.

2-*O*-Acetyl-4-*O*-benzyl-3-*O*-methyl-α-D-rhamnopyranoside-(1→4)-2-*O*-acetyl-3-*O*-methyl-α-D-rhamnopyranoside-(1→4)-2-*O*-acetyl-3-*O*-methyl-α-D-rhamnopyranoside-(1→4)-2-*O*-acetyl-3-*O*-methyl-α-D-rhamnopyranoside-(1→4)-2-*O*-acetyl-1-*O*-[(*N*-ethyl)-*tert*-butoxycarbonyl]-3-*O*-methyl-α-D-rhamnopyranoside (**4 penta**)

Same procedure as **2**. **5 tetra** (878.9 mg, 0.91 mmol), **S1** (452.9 mg, 1.09 mmol), anhydrous CH_2Cl_2 (50 mL), 0.200 g of activated powdered 3 Å molecular sieves, *N*-iodosuccinimide (348.8 mg, 1.27 mmol) and triflic acid (0.15 mL, 1.70 mmol). **4 penta** (693.0 mg, 0.55 mmol, 64 %) 693.0 mg, 0.55 mmol, 64 %) was isolated a clear oil. Rf = 0.50 (EtOAc/Hexane, 3/2) $[\alpha]_D^{25}$ 1.5 (*c* 0.27, $CHCl_3$) ^{1}H NMR (600 MHz, $CDCl_3$): δ 7.39-7.27 (m, 5H, Bn), 5.36-5.31 (m, 4H, H2′, H2′′, H2′′′, H2′′′′), 5.28 (s, 1H, H2), 5.12-5.08 (m, 4H, H1′, H1′′, H1′′′, H1′′′′), 4.88 (d, 1H, $J_{A,B}$ = 10.9 Hz, CHA, OBn), 4.83 (br. s, 1H, NH), 4.70 (d, 1H, $J_{1,2}$ = 1.1 Hz, H1), 4.61 (d, 1H, $J_{B,A}$ = 10.9 Hz, CHB, OBn), 3.86-3.71 (m, 4H, H5′, H5′′, H5′′′, H5′′′′), 3.71-3.64 (m, 2H, H5, CHA, O<u>CH</u>$_2$CH$_2$NH), 3.59 (dd, 1H, $J_{3'''',2''''}$ = 3.2 Hz, $J_{3'''',4''''}$ = 9.3 Hz, H3′′′′), 3.57-3.46 (m, 9H, H3, H3′, H3′′, H3′′′, H4, H4′, H4′′, H4′′′, CHB, O<u>CH$_2$</u>CH$_2$NH), 3.44 (s, 3H, OCH_3), 3.41-3.34 (m, 14H, 4x OCH_3, H4′′′′, CHA, OCH$_2$<u>CH$_2$</u>NH), 3.34-3.23 (m, 1H, CHB, OCH$_2$<u>CH$_2$</u>NH), 2.13 (s, 3H, Ac), 2.10 (s, 3H, Ac), 2.08 (s, 9H, 3x Ac), 1.46 (s, 9H, CH_3, Boc), 1.36-1.22 (m, 15H, H6, H6′, H6′′, H6′′′, H6′′′′) ^{13}C NMR (150 MHz, $CDCl_3$): δ 170.4, 170.3, 170.2, 170.1 (2C, C=O), 155.9 (C=O, Boc), 138.6, 128.5 (2C), 128.1 (2C), 127.9 (Bn), 99.7 (C1′), 99.6 (C1′′), 99.5 (C1′′′), 99.4 (C1′′′′), 97.9 (C1), 80.1 (4C, C3′, C3′′, C3′′′, C4′′′′), 80.0 (C3), 79.9 (C3′′′′), 79.7 (<u>C</u>(CH_3)$_3$, Boc), 78.8 (C4), 78.5 (C4′), 78.4 (C4′′), 78.3 (C4′′′), 75.5 (CH_2, Bn), 68.7 (C2′′′′), 68.5 (C5′′′′), 68.0 (3C, C2′, C2′′, C2′′′), 67.9 (C2), 67.7 (3C, C5′, C5′′, C5′′′), 67.3 (O<u>C</u>H$_2$CH$_2$NH), 67.1 (C5), 57.6 (OCH_3), 57.5 (OCH_3), 57.4 (3C, OCH_3), 40.4 (OCH$_2$<u>C</u>H$_2$NH), 28.5 (CH_3, Boc), 21.2, 21.1 (4C, 5x Ac), 18.4 (C6′), 18.3 (C6′′), 18.2 (C6′′′), 18.1 (C6), 17.9 (C6′′′′). $J_{C1,H1}$ = 173 Hz, ESI-MS: *m/z* Calcd for $C_{59}H_{91}O_{28}NH$ [M+H]$^+$, 1262.5800; found, 1262.5806.

2-*O*-Acetyl-3-*O*-methyl-α-ᴅ-rhamnopyranoside-(1→4)-2-*O*-acetyl-1-*O*-[(*N*-ethyl)-*tert*-butoxycarbonyl]-3-*O*-methyl-α-ᴅ-rhamnopyranoside (**5 di**)

Same procedure as **3**. $Pd(OH)_2$ (1.20 g, 8.55 mmol), MeOH (50 mL) and **4 di** (1.71 g, 2.61 mmol). **5 di** (1.47 g, 2.59 mmol, 99 %) was isolated as a clear oil. Rf = 0.20 (EtOAc/Hexane, 1/1) $[\alpha]_D^{25}$ 1.07 (*c* 0.33, $CHCl_3$) ^{1}H NMR (600 MHz, $CDCl_3$): δ 5.36 (dd, 1H, $J_{2',1'}$ = 1.6 Hz, $J_{2',3'}$ = 2.9 Hz, H2′), 5.31-5.26 (m, 1H, H2), 5.14 (d, 1H, $J_{1',2'}$ = 1.3 Hz, H1′), 4.83 (br. s, 1H, NH), 4.70 (d, 1H, $J_{1,2}$ = 1.4 Hz, H1), 3.84-3.75 (m, 1H, H5′), 3.74-3.70 (m, 1H, CHA, O<u>CH$_2$</u>CH$_2$NH), 3.70-3.64 (m, 1H, H5), 3.58-3.44 (m, 4H, H3, H4, H4′, CHB, O<u>CH$_2$</u>CH$_2$NH), 3.43-3.40 (m, 4H, H3′, OCH_3), 3.40-3.33 (m, 4H, OCH_3, CHA, OCH$_2$<u>CH$_2$</u>NH), 3.33-3.25 (m, 1H, CHB, OCH$_2$<u>CH$_2$</u>NH), 2.37 (br. s, 1H, OH), 2.12-2.08 (m, 6H, Ac), 1.46 (s, 9H, CH_3, Boc), 1.32 (d, 3H, $J_{6',5'}$ = Hz, 6.2 Hz, H6′), 1.31 (d, 3H, $J_{6,5}$ = 6.2 Hz, H6) ^{13}C NMR (150 MHz, $CDCl_3$): δ 170.5, 170.2 (C=O), 155.9 (C=O, Boc), 99.8 (C1′), 97.9 (C1), 80.1 (C3), 79.7 (<u>C</u>(CH_3)$_3$, Boc), 79.5 (C3′), 78.4 (C4), 71.7 (C4′), 69.0 (C5′), 67.9 (C2), 67.5 (C2′), 67.4 (O<u>C</u>H$_2$CH$_2$NH), 67.2 (C5), 57.5 (OCH_3), 57.3 (OCH_3), 40.4 (OCH$_2$<u>C</u>H$_2$NH), 28.6 (CH_3, Boc), 21.2, 21.1 (2x Ac), 18.3 (C6′), 17.7 (C6). $J_{C1,H1}$ = 173 Hz, ESI-MS: *m/z* Calcd for $C_{32}H_{43}O_{13}NH$ [M+H]$^+$, 566.2807; found, 566.2788.

2-*O*-Acetyl-3-*O*-methyl-α-ᴅ-rhamnopyranoside-(1→4)-2-*O*-acetyl-3-*O*-methyl-α-ᴅ-rhamnopyranoside-(1→4)-2-*O*-acetyl-1-*O*-[(*N*-ethyl)-*tert*-butoxycarbonyl]-3-*O*-methyl-α-ᴅ-rhamnopyranoside (**5 tri**)

Same procedure as **3**. $Pd(OH)_2$ (807.0 mg, 5.67 mmol), MeOH (50 mL) and **4 tri** (1.65 g, 1.92 mmol). **5 tri** (1.57 g, 1.89 mmol, 98 %) was isolated as a clear oil. Rf = 0.20 (EtOAc/Hexane, 1/1) $[\alpha]_D^{25}$ 2.3 (*c* 0.51, $CHCl_3$) ^{1}H NMR (600 MHz, CD_3OD): δ 5.40 (s, 1H, H2′), 5.36 (s, 1H, H2′′), 5.32 (dd, 1H, $J_{2,1}$ = 1.7 Hz, $J_{2,3}$ = 3.2 Hz, H2), 5.10 (d, 1H, $J_{1'',2''}$ = 1.6 Hz, H1′′), 5.06 (d, 1H, $J_{1',2'}$ = 1.3 Hz, H1′), 4.75 (s, 1H, H1), 3.88-3.82 (m, 1H, H5′), 3.81-3.75 (m, 2H, H5, H5′′), 3.75-3.71 (m, 1H, CHA, O<u>CH$_2$</u>CH$_2$NH), 3.69 (dd, 1H, $J_{3,2}$ = 3.3 Hz, $J_{3,4}$ = 9.4 Hz, H3), 3.57 (dd, 1H, $J_{3',2'}$ = 3.2 Hz, $J_{3',4'}$ = 9.4 Hz, H3′), 3.54-3.44 (m, 3H, H4, H4′, CHB, O<u>CH$_2$</u>CH$_2$NH), 3.43 (s, 3H, OCH_3), 3.42-3.37 (m, 8H, H3′′, H4′′, 2x OCH_3), 3.32-3.24 (m, 2H, CHA, CHB, OCH$_2$<u>CH$_2$</u>NH), 2.13-2.11 (m, 6H, 2x Ac), 2.10 (s, 3H, Ac), 1.50 (s, 9H, CH_3, Boc), 1.32 (d, 3H, $J_{6',5'}$ = 6.2 Hz, H6′), 1.31 (d, 3H, $J_{6'',5''}$ = 6.2 Hz, H6′′), 1.29 (d, 3H, $J_{6,5}$ = 6.2 Hz, H6) ^{13}C NMR (150 MHz, CD_3OD): δ 171.7 (2C), 171.6 (C=O), 158.5 (C=O,

Boc), 101.0 (C1′), 100.8 (C1″), 98.7 (C1), 81.5 (C3′), 81.3 (C3), 80.8 (C4), 80.4 (C3″), 80.0 ($\underline{C}(CH_3)_3$, Boc), 79.6 (C4′), 72.9 (C4″), 70.8 (C5″), 69.6 (C2″), 69.3 (C2), 69.2 (C2′), 69.0 (C5′), 68.0 (C5), 67.5 ($O\underline{C}H_2CH_2NH$), 58.0 (OCH_3), 57.7 (2C, OCH_3), 41.1 ($OCH_2\underline{C}H_2NH$), 28.9 (CH_3, Boc), 20.7 (3C, Ac), 18.6 (C6′), 18.5 (C6″), 17.9 (C6). $J_{C1,H1}$ = 172 Hz, ESI-MS: *m/z* Calcd for $C_{34}H_{57}O_{18}NNa$ $[M+Na]^+$, 790.3468; found, 790.3462.

2-*O*-Acetyl-3-*O*-methyl-α-ᴅ-rhamnopyranoside-(1→4)-2-*O*-acetyl-3-*O*-methyl-α-ᴅ-rhamnopyranoside-(1→4)-2-*O*-Acetyl-3-*O*-methyl-α-ᴅ-rhamnopyranoside-(1→4)-2-*O*-acetyl-1-*O*-[(*N*-ethyl)-*tert*-butoxycarbonyl]-3-*O*-methyl-α-ᴅ-rhamnopyranoside (**5 tetra**)

Same procedure as **3**. $Pd(OH)_2$ (720.0 mg, 5.13 mmol), MeOH (50 mL) and **4 tetra** (1.42 g, 1.34 mmol). **5 tetra** (1.30 g, 1.34 mmol, 100 %) was isolated as a clear oil. Rf = 0.15 (EtOAc/Hexane, 3/2) $[\alpha]_D^{25}$ 3.4 (*c* 0.67, $CHCl_3$) ^1H NMR (600 MHz, CD_3OD): δ 5.42 (dd, 1H, $J_{2',1'}$ = 1.5 Hz, $J_{2',3'}$ = 3.3 Hz, H2′), 5.40 (dd, 1H, $J_{2'',1''}$ = 2.0 Hz, $J_{2'',3''}$ = 2.8 Hz, H2″), 5.38 (dd, 1H, $J_{2''',1'''}$ = 1.8 Hz, $J_{2''',3'''}$ = 2.8 Hz, H2‴), 5.33 (dd, 1H, $J_{2,1}$ = 1.8 Hz, $J_{2,3}$ = 3.2 Hz, H2), 5.10 (d, 1H, $J_{1''',2'''}$ = 1.7 Hz, H1‴), 5.08 (d, 1H, $J_{1'',2''}$ = 1.7 Hz, H1″), 5.06 (d, 1H, $J_{1',2'}$ = 1.4 Hz, H1′), 4.75 (d, 1H, $J_{1,2}$ = 1.8 Hz, H1), 3.90-3.82 (m, 2H, H5′, H5″), 3.82-3.76 (m, 2H, H5, H5‴), 3.76-3.72 (m, 1H, CHA, $O\underline{CH_2}CH_2NH$), 3.72-3.66 (dd, 1H, $J_{3,2}$ = 3.3 Hz, $J_{3,4}$ = 9.4 Hz, H3), 3.59 (dd, 1H, $J_{3',2'}$ = 3.2 Hz, $J_{3',4'}$ = 9.3 Hz, H3′), 3.57 (dd, 1H, $J_{3'',2''}$ = 2.9 Hz, $J_{3'',4''}$ = 7.7 Hz, H3″), 3.55-3.46 (m, 4H, H4′, H4″, OH, CHB, $O\underline{CH_2}CH_2NH$), 3.44 (s, 3H, OCH_3), 3.43-3.36 (m, 11H, 3x OCH_3, H3‴, H4‴), 3.33-3.22 (m, 2H, CHA, CHB, $OCH_2\underline{CH_2}NH$), 2.14-2.11 (m, 9H, 3x Ac), 2.11 (s, 3H, Ac), 1.51 (s, 9H, CH_3, Boc), 1.36-1.31 (m, 9H, H6, H6′, H6″), 1.30 (d, 3H, H6‴) ^{13}C NMR (150 MHz, CD_3OD): δ 171.7 (2C), 171.6 (2C, C=O), 158.5 (C=O, Boc), 101.0 (C1′), 100.9 (C1″), 100.8 (C1‴), 98.7 (C1), 81.4 (C3′), 81.3 (C3″), 81.2 (C3), 80.9 (C4), 80.3 (C3‴), 80.2 (C4′), 80.0 ($\underline{C}(CH_3)_3$, Boc), 79.6 (C4″), 72.9 (C4‴), 70.7 (C5‴), 69.6 (C2‴), 69.3 (C2), 69.2 (C2″), 69.1 (C2′), 69.0 (C5′), 68.9 (C5″), 68.0 (C5), 67.6 ($O\underline{C}H_2CH_2NH$), 58.0 (OCH_3), 57.6 (3C, OCH_3), 41.1 ($OCH_2\underline{C}H_2NH$), 29.0 (CH_3, Boc), 20.7 (4C, Ac), 18.6 (C6), 18.5 (C6″), 18.4 (C6′), 17.9 (C6‴). $J_{C1,H1}$ = 173 Hz, ESI-MS: *m/z* Calcd for $C_{43}H_{71}O_{23}NNa$ $[M+Na]^+$, 992.4309; found, 992.4313.

2-*O*-Acetyl-3-*O*-methyl-α-ᴅ-rhamnopyranoside-(1→4)-2-*O*-acetyl-3-*O*-methyl-α-ᴅ-rhamnopyranoside-(1→4)-2-*O*-acetyl-3-*O*-methyl-α-ᴅ-rhamnopyranoside-(1→4)-2-*O*-acetyl-3-*O*-methyl-α-ᴅ-rhamnopyranoside-(1→4)-2-*O*-acetyl-1-*O*-[(*N*-ethyl)-*tert*-butoxycarbonyl]-3-*O*-methyl-α-ᴅ-rhamnopyranoside (**5 penta**)

Same procedure as **3**. $Pd(OH)_2$ (300.0 mg, 2.14 mmol), MeOH (30 mL) and **4 penta** (623.3 mg, 0.49 mmol). **5 penta** (537.0 mg, 0.46 mmol, 93 %) was isolated as a clear oil. Rf = 0.25 (EtOAc/Hexane, 3/2) $[\alpha]_D^{25}$ 0.12 (*c* 0.033, $CHCl_3$) ^1H NMR (600 MHz, CD_3OD): δ 5.44-5.41 (m, 3H, H2′, H2″, H2‴), 5.39 (m, 1H, H2⁗), 5.34 (dd, 1H, $J_{2,1}$ = 1.7 Hz, $J_{2,3}$ = 3.1 Hz, H2), 5.11 (d, 1H, $J_{1'''',2''''}$ = 1.3 Hz, H1⁗), 5.09 (s, 2H, H1″, H1‴), 5.07 (s, 1H, H1′), 4.76 (s, 1H, H1), 3.92-3.83 (m, 3H, H5′, H5″, H5‴), 3.83-3.77 (m, 2H, H5, H5⁗), 3.77-3.73 (m, 1H, CHA, $O\underline{CH_2}CH_2NH$), 3.70 (dd, 1H, $J_{3,2}$ = 3.1 Hz, $J_{3,4}$ = 9.3 Hz, H3), 3.62-3.56 (m, 3H, H3′, H3″, H3‴), 3.56-3.46 (m, 5H, H4, H4′, H4″, H4‴, CHB, $OCH_2\underline{CH_2}NH$), 3.45 (3H, OCH_3), 3.45-3.38 (m, 14H, 4x OCH_3, H3⁗, H4⁗), 3.34-3.24 (m, 2H, CHA, CHB, $OCH_2\underline{CH_2}NH$), 2.14-2.12 (m, 12H, 4x Ac), 2.11 (s, 3H, Ac), 1.52 (s, 9H, CH_3, Boc), 1.36-1.32 (m, 12H, H6, H6′, H6″, H6‴), 1.31 (d, 3H, H6⁗) ^{13}C NMR (150 MHz, CD_3OD): δ 171.7 (2C), 171.6 (3C, C=O), 158.4 (C=O, Boc), 101.0 (C1′), 100.8 (3C, C1″,C1‴, C1⁗), 98.6 (C1), 81.4 (C3′), 81.3 (2C, C3″, C3‴), 81.2 (C3), 80.9 (C4), 80.3 (C3⁗), 80.2 (C4′), 80.1 (C4″), 80.0 ($\underline{C}(CH_3)_3$, Boc), 79.6 (C4‴), 72.9 (C4⁗), 70.7 (C5⁗), 69.6 (C2⁗), 69.3 (2C, C2, C2″), 69.2 (C2′), 69.1 (2C, C2‴, C5′), 69.0 (C5″), 68.9 (C5‴), 68.0 (C5), 67.5 ($O\underline{C}H_2CH_2NH$), 58.0 (OCH_3), 57.6 (4C, OCH_3), 41.1 ($OCH_2\underline{C}H_2NH$), 29.0 (CH_3, Boc), 20.7 (5C, Ac), 18.6 (C6), 18.5 (3C, C6′, C6″, C6‴), 17.9 (C6⁗). $J_{C1,H1}$ = 173 Hz, ESI-MS: *m/z* Calcd for $C_{52}H_{85}O_{28}NNa$ $[M+Na]^+$, 1194.5150; found, 1194.5153.

Inhibition ELISA assay

In order to ensure that the necessary conformation to mimic the natural antigen had been retained in the synthetic oligosaccharides with handle and spacer, **1 tri**, **1 tetra**, and **1 penta** were screened in inhibition ELISA vs mAb 1B1 that was raised to the natural antigen[5b]

If enough LPS / oligosaccharide is present in the inhibition step to bind all of the available 1B1, then there will be no free 1B1 to bind the LPS on the ELISA plate and thus there will be no color reaction in the ELISA. However, if the LPS / oligosaccharide either does not bind the antibody, or there is not enough LPS / oligosaccharide to block the 1B1 binding then the intensity of the color generated in the ELISA will be similar to incubation with PBS alone.

As shown in **Figure S1** Pa wt LPS binds and blocks 1B1, as no color is generated in ELISA until the LPS is diluted to ~ 100μg/ml. The irrelevant Nm LPS does not block 1B1 from binding, nor does the PBS.

The oligosaccharides block 1B1 binding with the tetra- and pentaaccharide behaving similarly and only being titered out at ~ 5μg/ml, whereas the tri-saccharide also blocks binding but titers out earlier at ~100μg/ml.

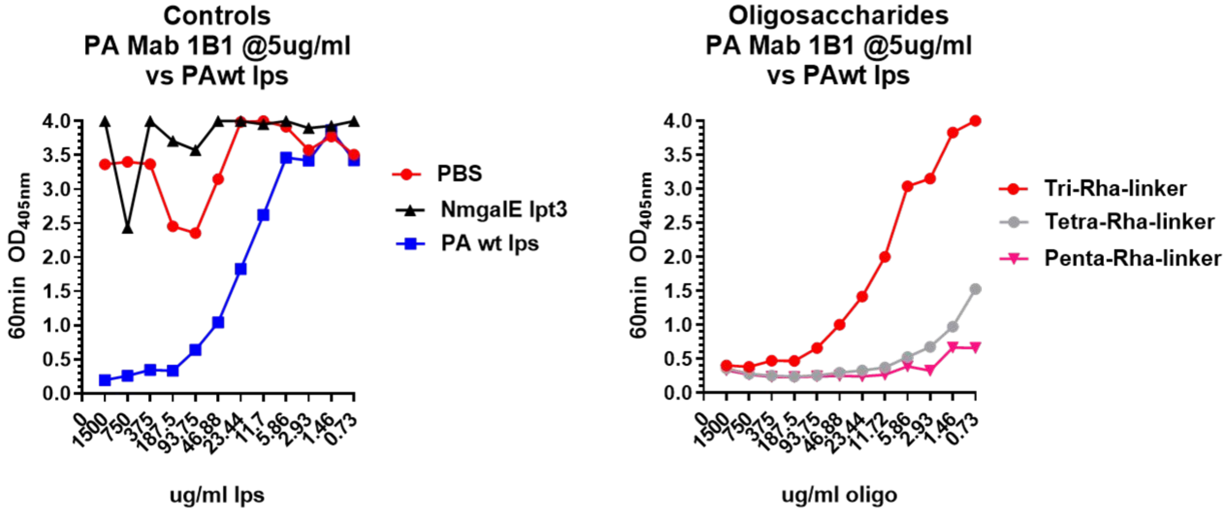

Figure S1. Inhibition ELISA of mAb 1B1 with LPS (left-hand graph; blue, *P. aeruginosa* PAO1 BAA-47 (wt); black, *N. meningitidis galE/lpt3*; red, PBS control) or synthetic oligosaccharides with handle representing the terminal methylated rhamnan (right-hand graph; pink, pentasaccharide; grey, tetrasaccharide; red, trisaccharide; against *P. aeruginosa* PAO1 BAA-47 (wt) LPS. Serial dilution, as shown on the x axis.

These results therefore corroborate with the data from earlier prepared oligosaccharides (without handle). The addition of the handle in these oligosaccharides does not alter the conformation of the oligosaccharide nor affect the oligosaccharide from binding and blocking mAb 1B1. Thus, the synthesised oligosaccharides with handle effectively mimic the epitope recognised by mAb 1B1 on Pa.

Conjugation of oligosaccharide to activated CRM_{197} and activated BSA to prepare glycoconjugates

Table S1. Summary of activation and oligosaccharide loading of CRM & BSA conjugates

Conjugate	Amino-oxy activation (# of amino-oxy / protein)	Conjugation (# of oligos / protein)
CRM-tri	15	10
CRM-tetra-	15	9
CRM-penta	15	8
BSA-tri	20	6
BSA-tetra-	20	5
BSA-penta-	20	6

CRM_{197} or BSA (10 mg/ml) were dissolved in sodium phosphate buffer (200mM, pH7.4) and kept at 4°C. Then bromoacetic acid *N*-hydroxysuccinimide ester (85X molar excess, 3mg/mL) was dissolved in DMSO and added to CRM_{197} or BSA. After 18h at 4°C, the bromine-activated protein was desalted using an Amicon ultra-10 30K MWCO filter against water (3x) to an approximate volume of 500µl. To the concentrated protein, 500µl of 200mM sodium phosphate buffer (pH7.4). was added and cooled to 4°C. 3-(aminooxy)-1-propanethiol hydrochloride (75X) dissolved in sodium phosphate buffer (100µl, 200 mM, pH 7.4) was added to the brominated protein solution. After 2h at RT, the resulting aminooxy-activated protein was then desalted using an Amicon ultra-10 30K MWCO filter against water (3X). Aminooxy/protein ratio were determined by MALDI MS (**Table S1**).

Screening of derived mice sera vs. BSA-conjugates and LPS:

Individual sera from mice that had received a prime and two boost immunisation schedules were screened for their ability to recognise the BSA-oligosaccharide conjugates and Pa wt LPS in ELISA.

All mice produced a good IgM response to the conjugates as illustrated by their recognition of the BSA-oligosaccharide conjugates (**Figure S4, S5 and S6**) in ELISA relative to the pre-immune sera (**Figure S2**) and a moderate response to the LPS (**Figure S7**) relative to the pre-immune sera (**Figure S3**). All mice produced a moderate IgG response to the conjugates as illustrated by their recognition of the BSA-oligosaccharide conjugates (**Figure S10**) in ELISA relative to the pre-immune sera (**Figure S8**). Similarly, all mice that received the tetra- and penta-saccharide conjugates produced a moderate IgG response to the conjugates as illustrated by their recognition of the LPS (**Figure S11**) in ELISA relative to the pre-immune sera (**Figure S9**). Since mice that received immunisations with the CRM-tetra- and pentasaccharide conjugates showed an improved IgG response to Pa wt LPS in ELISA relative to mice that received the tri-saccharide conjugate (**Figure S11**), it may be suggested from the mice data that the minimum length of oligosaccharide required to effectively mimic the natural antigen is a tetra-saccharide. Killed whole cells as detailed in **Table S2** were screened in ELISA for recognition by the generated sera.

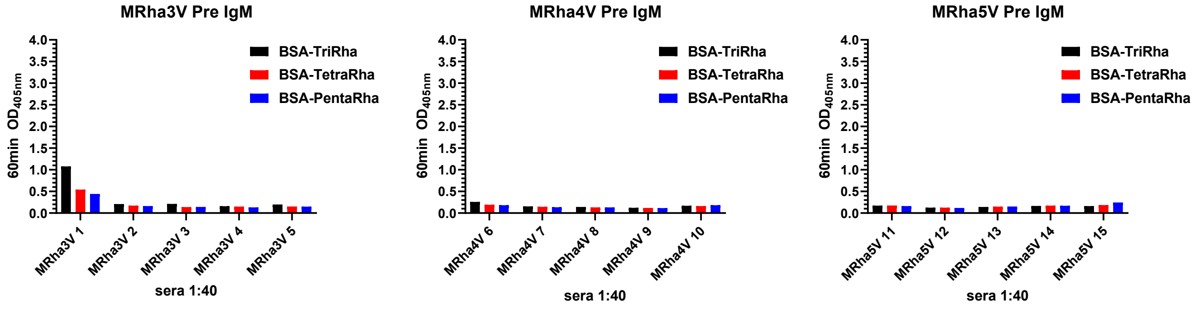

Figure S2. ELISA determined recognition with pre-immune mice sera IgM (1:40 dilution) prior to CRM-oligosaccharide conjugate immunisation vs BSA-oligosaccharide conjugates. Mice MRha3V 1-5 will receive the trisaccharide CRM conjugate, mice MRha4V 6-10 will receive the tetrasaccharide CRM conjugate and mice MRha5V 11-15 will receive the pentasaccharide CRM conjugate

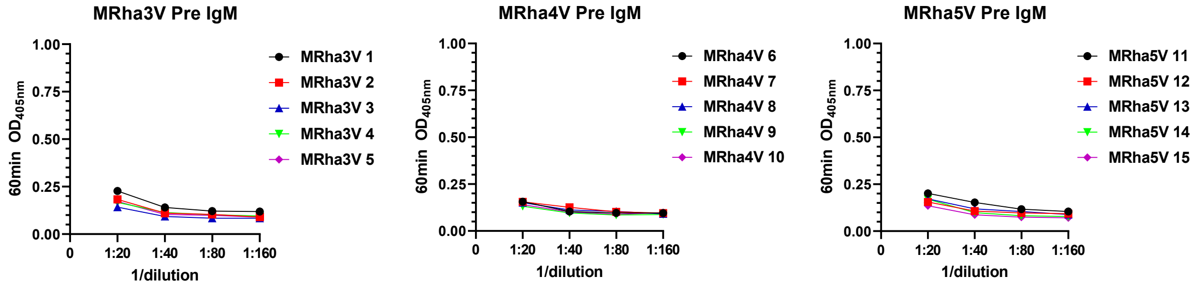

Figure S3. ELISA determined recognition with pre-immune mice sera IgM titration prior to CRM-oligosaccharide conjugate immunisation vs Pa wt LPS. Mice MRha3V 1-5 will receive the trisaccharide CRM conjugate, mice MRha4V 6-10 will receive the tetrasaccharide CRM conjugate and mice MRha5V 11-15 will receive the pentasaccharide CRM conjugate

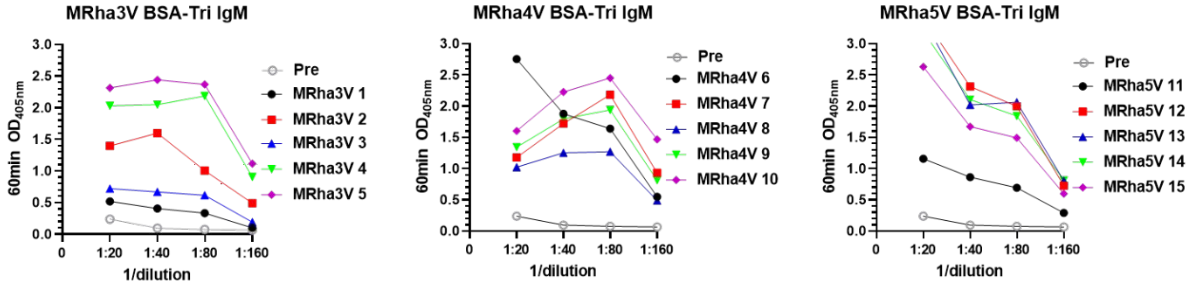

Figure S4 ELISA determined recognition with final bleed mice sera IgM titration following CRM-oligosaccharide conjugate immunisation vs BSA-trisaccharide conjugates. Mice MRha3V 1-5 received the trisaccharide CRM conjugate, mice MRha4V 6-10 received the tetrasaccharide CRM conjugate and mice MRha5V 11-15 received the pentasaccharide CRM conjugate

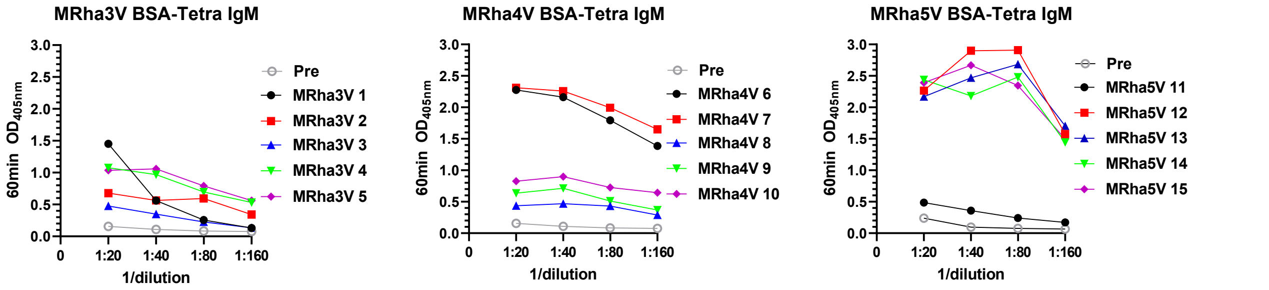

Figure S5 ELISA determined recognition with final bleed mice sera IgM titration following CRM-oligosaccharide conjugate immunisation vs BSA-tetrasaccharide conjugates. Mice MRha3V 1-5 received the trisaccharide CRM conjugate, mice MRha4V 6-10 received the tetrasaccharide CRM conjugate and mice MRha5V 11-15 received the pentasaccharide CRM conjugate

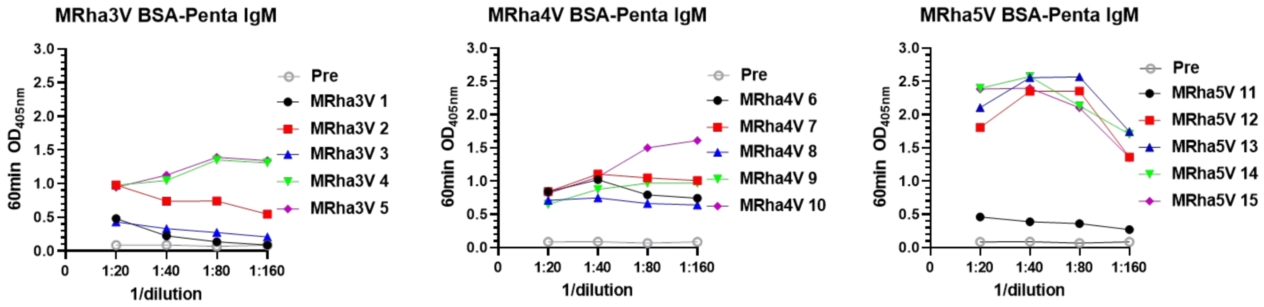

Figure S6 ELISA determined recognition with final bleed mice sera IgM titration following CRM-oligosaccharide conjugate immunisation vs BSA-pentasaccharide conjugates. Mice MRha3V 1-5 received the trisaccharide CRM conjugate, mice MRha4V 6-10 received the tetrasaccharide CRM conjugate and mice MRha5V 11-15 received the pentasaccharide CRM conjugate

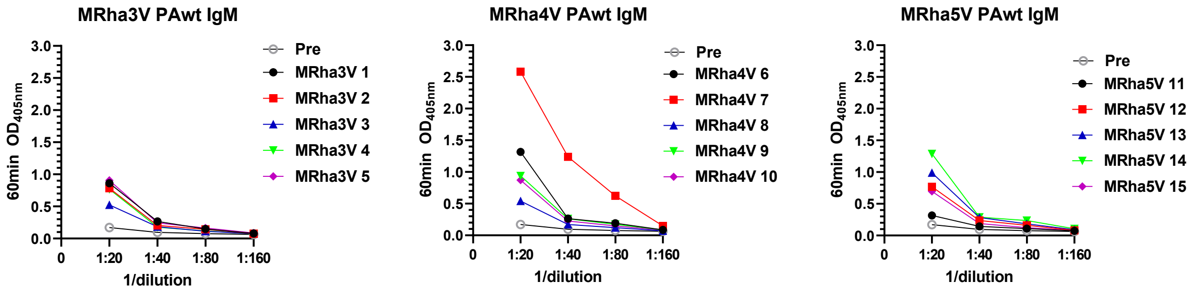

Figure S7 ELISA determined recognition with final bleed mice sera IgM titration following CRM-oligosaccharide conjugate immunisation vs LPS. Mice MRha3V 1-5 received the trisaccharide CRM conjugate, mice MRha4V 6-10 received the tetrasaccharide CRM conjugate and mice MRha5V 11-15 received the pentasaccharide CRM conjugate

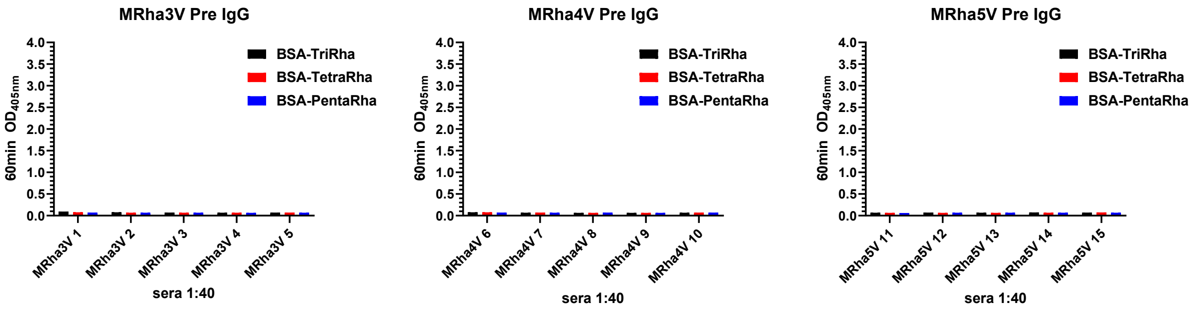

Figure S8 ELISA determined recognition with pre-immune mice sera IgG (1:40 dilution) prior to CRM-oligosaccharide conjugate immunisation vs BSA-oligosaccharide conjugates. Mice MRha3V 1-5 will receive the trisaccharide CRM conjugate, mice MRha4V 6-10 will receive the tetrasaccharide CRM conjugate and mice MRha5V 11-15 will receive the pentasaccharide CRM conjugate

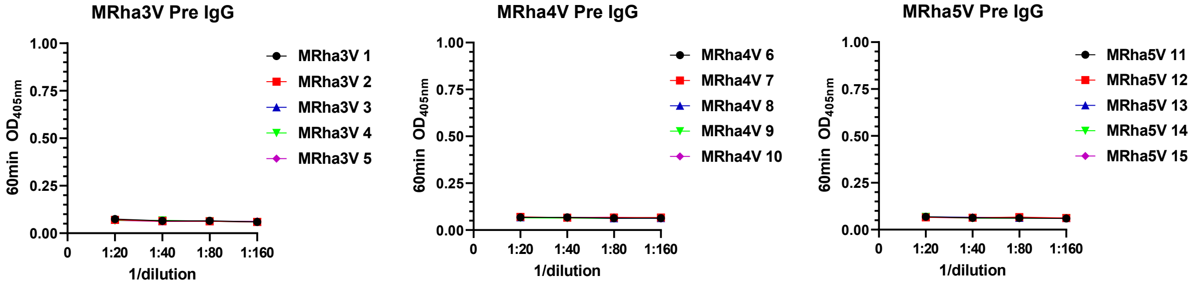

Figure S9 ELISA determined recognition with pre-immune mice sera IgG titration prior to CRM-oligosaccharide conjugate immunisation vs Pa wt LPS. Mice MRha3V 1-5 will receive the trisaccharide CRM conjugate, mice MRha4V 6-10 will receive the tetrasaccharide CRM conjugate and mice MRha5V 11-15 will receive the pentasaccharide CRM conjugate

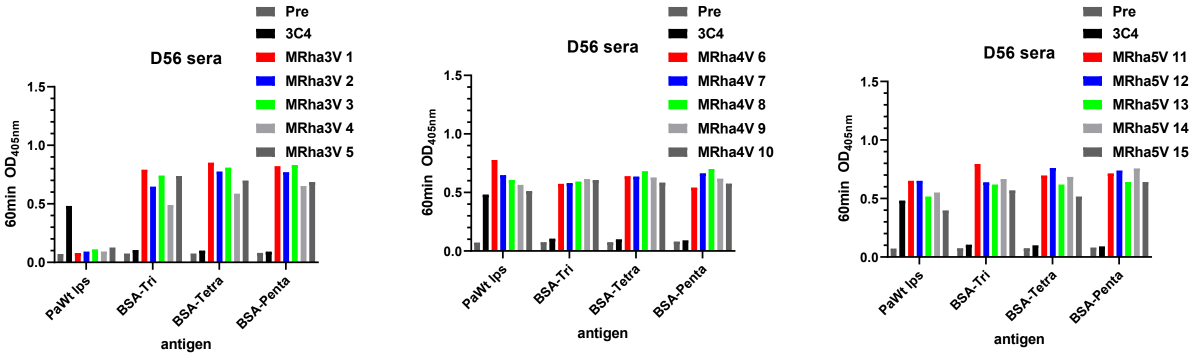

Figure S10 ELISA determined recognition with final bleed mice sera IgG (1:20 dilution) following CRM-oligosaccharide conjugate immunisation vs BSA-oligosaccharides and Pa wt LPS as indicated. Mice MRha3V 1-5 received the trisaccharide CRM conjugate, mice MRha4V 6-10 received the tetrasaccharide CRM conjugate and mice MRha5V 11-15 received the pentasaccharide CRM conjugate. Pre-immune sera

(Pre) was included as a negative control and mAb 3C4 (isotype IgG2b) was included as a positive control for Pa wt LPS

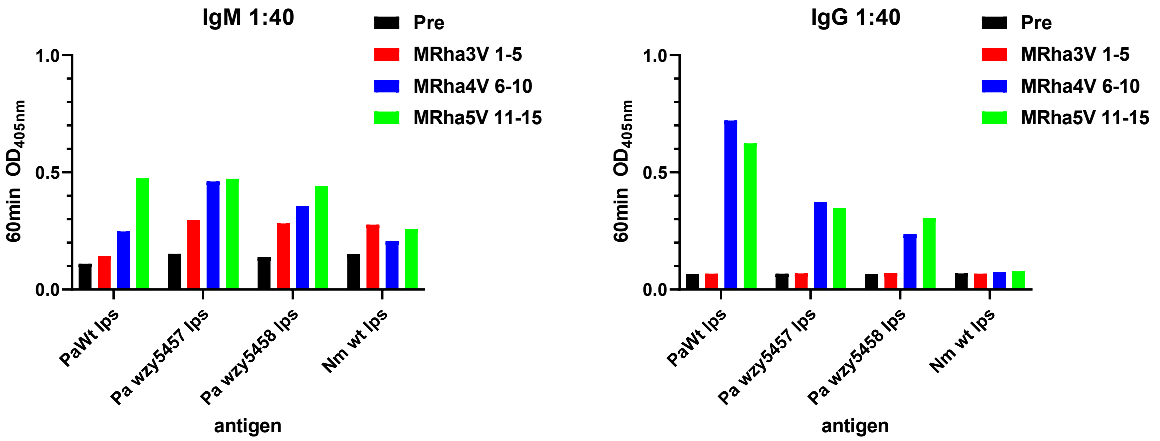

Figure S11. ELISA determined recognition with pooled pre- and final bleed mice sera (1:40 dilution) IgG following CRM-oligosaccharide conjugate immunisation vs Pa wt, Pa wzy5457, Pa wzy5458 and Nm wt (negative control) LPS as indicated. Mice MRha3V 1-5 received the trisaccharide CRM conjugate, mice MRha4V 6-10 received the tetrasaccharide CRM conjugate and mice MRha5V 11-15 received the pentasaccharide CRM conjugate. Pre-immune sera (Pre) was included as a negative control

Screening of derived rabbit sera vs. BSA-conjugates and LPS

All rabbits produced a good immune response to the conjugates as illustrated by their recognition of the BSA-oligosaccharide conjugates in ELISA relative to the pre-immune sera (**Figure S12**). All rabbits produced a strong response (end-point titers in the 1:10,000 range) that were capable of recognising several different LPS molecules.

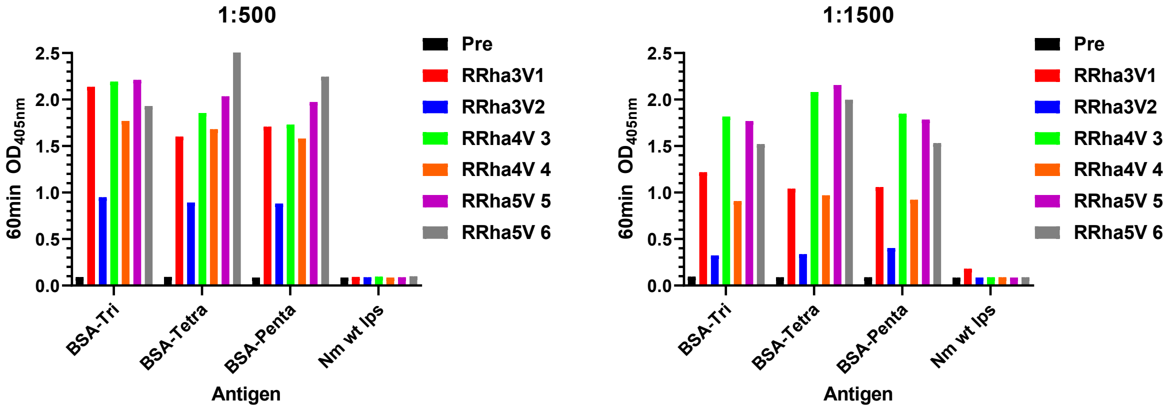

Figure S12 ELISA determined recognition with pre- and post-immune (D70) rabbit sera (1:500 and 1:1500 dilution) prior to and following CRM-oligosaccharide conjugate immunisation vs BSA-oligosaccharide

conjugates. Rabbits RRha3V 1-2 received the trisaccharide CRM conjugate, rabbits RRha4V 3-4 received the tetrasaccharide CRM conjugate and rabbits RRha5V 5-6 received the pentasaccharide CRM conjugate.

Table S2. List of cells screened in whole cell ELISA

Species	Strain	NRCC #	Serotype	Details	Source
P. aeruginosa	PAO1 BAA-47	6678	5	wild type	ATCC
	PAO1	6667	-	PAO1 (wzy ::Gm)	Lam lab
	PAO1	6668	-	PAO1 (wzy ::Gm)(Δpa5457)	Lam lab
	PAO1	6669	-	PAO1 (wzy ::Gm)(Δpa5458)	Lam lab
	PAO1	6670	-	PAO1 (wzy ::Gm)(Δpa5459)	Lam lab
	5933	6954	1	33348	ATCC
	5934	6955	2	33349	ATCC
	5939	6956	6	33354	ATCC
	5943	6957	10	33357	ATCC
	5944	6958	11	33358	ATCC
	170003	6959	16	33363	ATCC
	5937	6960	5	33352	ATCC
	001S4-1	6944	-	Clinical isolate	Sad lab
	003S-20	6945	-	Clinical isolate	Sad lab
	003E-9	6946	-	Clinical isolate	Sad lab
	004S-3	6947	-	Clinical isolate	Sad lab
	004E-8	6948	-	Clinical isolate	Sad lab
	006S3-1	6949	-	Clinical isolate	Sad lab
	006S4-1	6950	-	Clinical isolate	Sad lab
	009S-7	6951	-	Clinical isolate	Sad lab
	014S-1	6952	-	Clinical isolate	Sad lab
	014E-31	6953	-	Clinical isolate	Sad lab
M. catarrhalis	*lgt2/lgt4*	6541	-	Negative control	Cox lab
N. meningitidis	8047 *lpt3*	6263	-	Negative control	Cox lab

Screening of derived mice and rabbit sera vs. killed whole cells

Post-immune mice sera pooled by oligosaccharide vaccine that they received recognised the cells as shown in **Figure S13** Once again post-immune sera from the trisaccharide conjugates a weaker response when compared to the clear-cut response provoked by the tetra- and pentasaccharide conjugate illustrating that the methyl rhamnan tip epitope is visible in the context of whole cells and that the oligosaccharide conjugate was able to generate an immune response capable of recognising this epitope.

Post-immune individual rabbit sera recognised the cells as shown in **Figure S14** illustrating that the methyl rhamnan tip epitope is visible in the context of whole cells and that the oligosaccharide

conjugates (tri-, tetra- and penta-) were able to generate an immune response capable of recognizing this epitope.

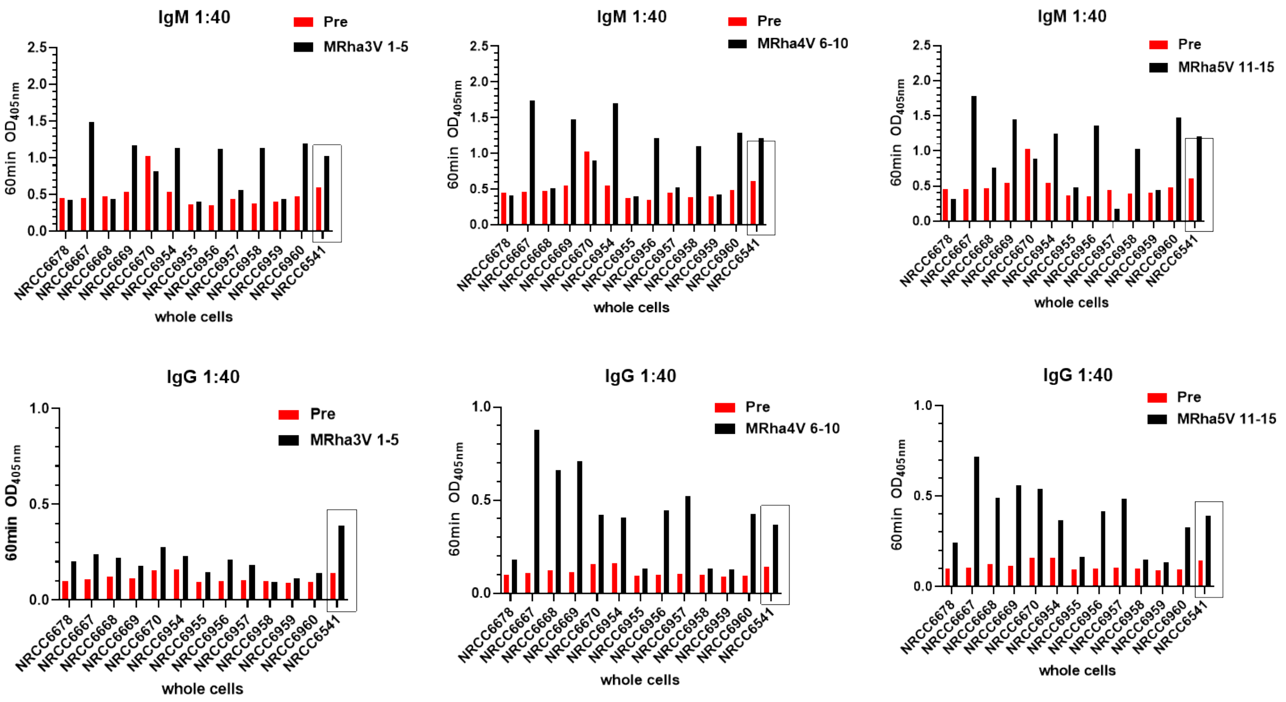

Figure S13 ELISA analysis of binding of pooled mice sera pre- and post-immune (IgM and IgG all at 1:40 dilution) to killed whole cells of *Pa* strains NRCC #'s 6678, 6667-70, 6954-60 and negative control strain *M. catarrhalis* 6541 (see **Table S2** for full details of strains).

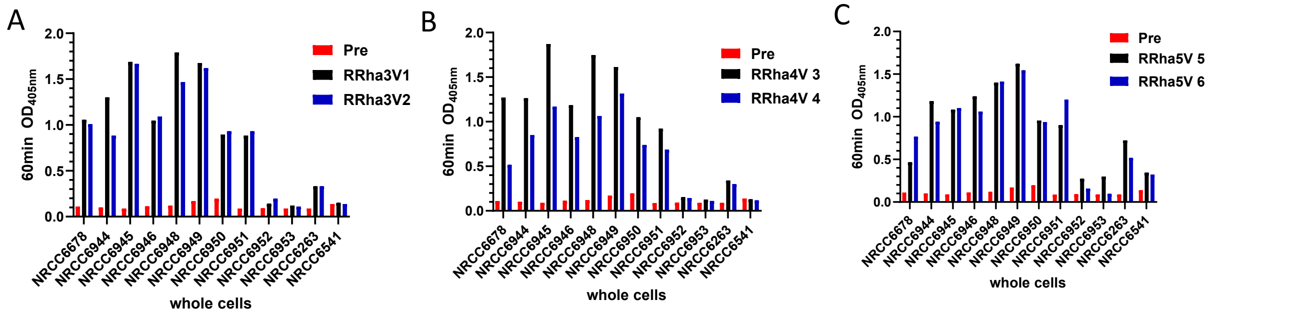

Figure S14 ELISA analysis of binding of individual rabbit sera (A) RRha3V 1-2 received the trisaccharide CRM conjugate (B) RRha3V 1-2 received the tetrasaccharide CRM conjugate (C) RRha3V 1-2 received the pentasaccharide CRM conjugate; pre- and post-immune (all at 1:1500 dilution) to killed whole cells of P. aeruginosa strains NRCC #'s 6678, 6944-53, 6263 and negative control strain M. catarrhalis 6541 (see Table S2 for full details of strains).

NMR Spectra

^{1}H NMR, **S3** (400 MHz, CD_3OD)

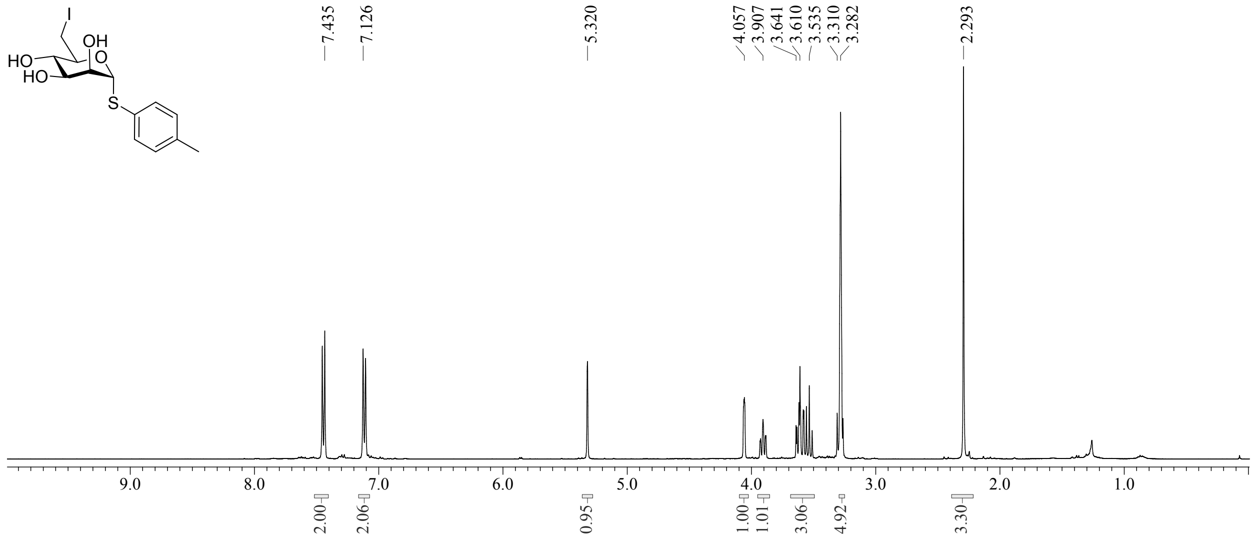

^{13}C NMR {H}, **S3** (100 MHz, CD_3OD)

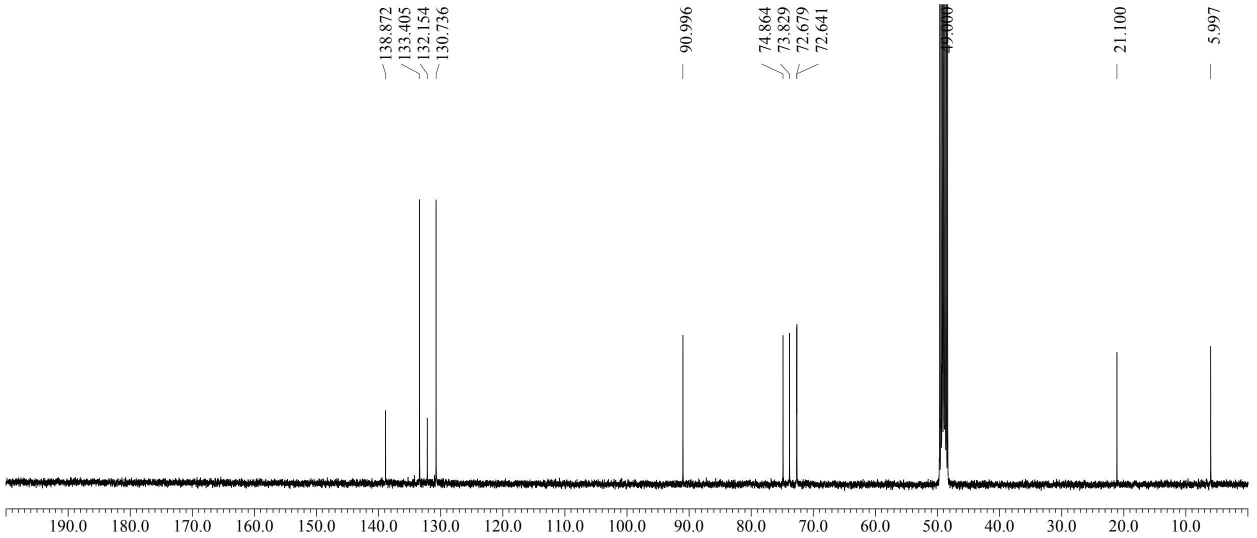

^{1}H NMR, **S4** (400 MHz, CD_3OD)

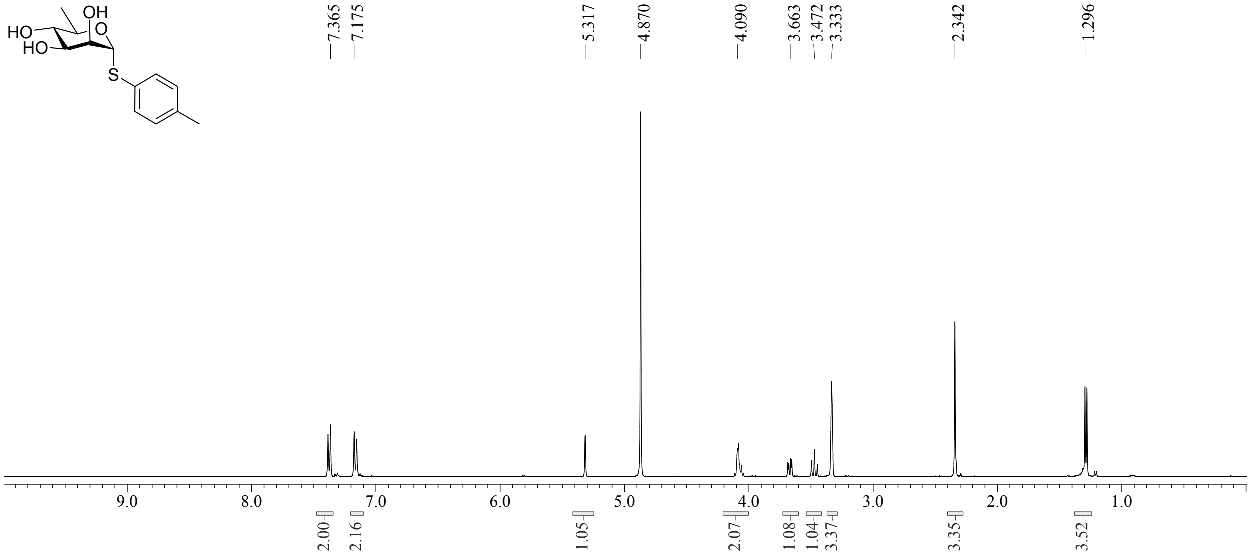

^{13}C {H} NMR {H}, **S4** (100 MHz, $CDCl_3$)

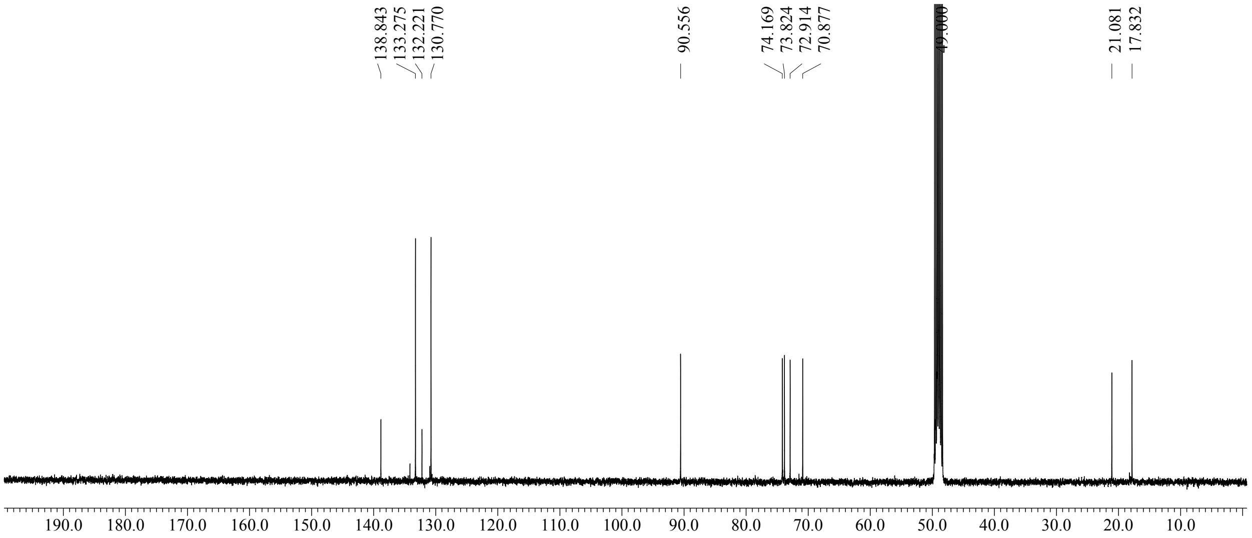

^{1}H NMR, **S8** (400 MHz, CDCl$_3$)

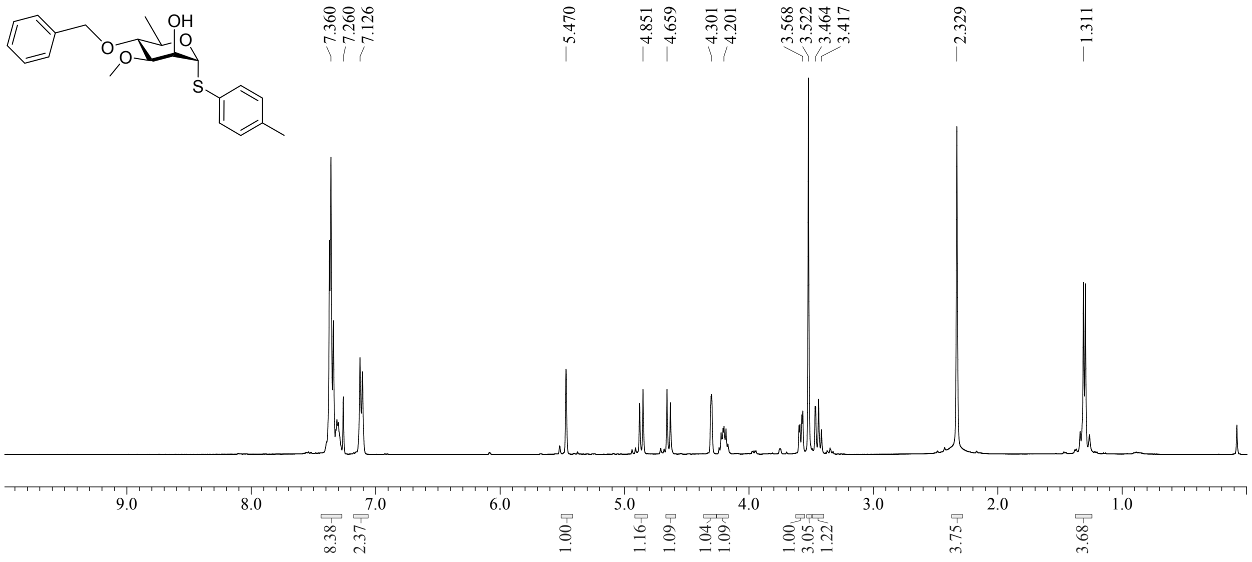

^{13}C NMR {H}, **S8**(100 MHz, CDCl$_3$)

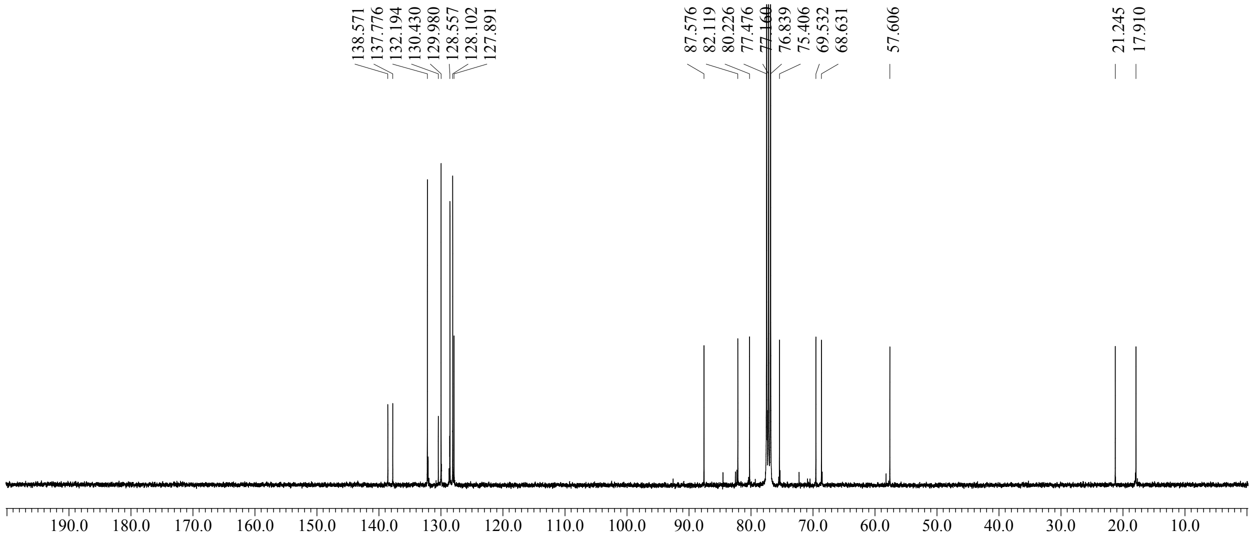

^{1}H NMR, **2** (500 MHz, $CDCl_3$)

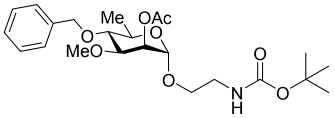

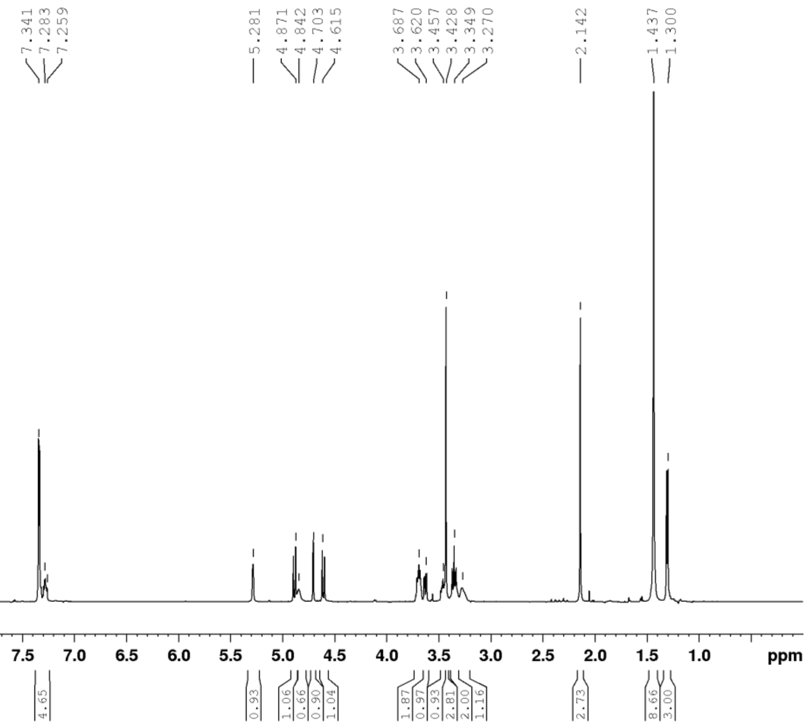

^{13}C {H} NMR, **2** (125 MHz, $CDCl_3$)

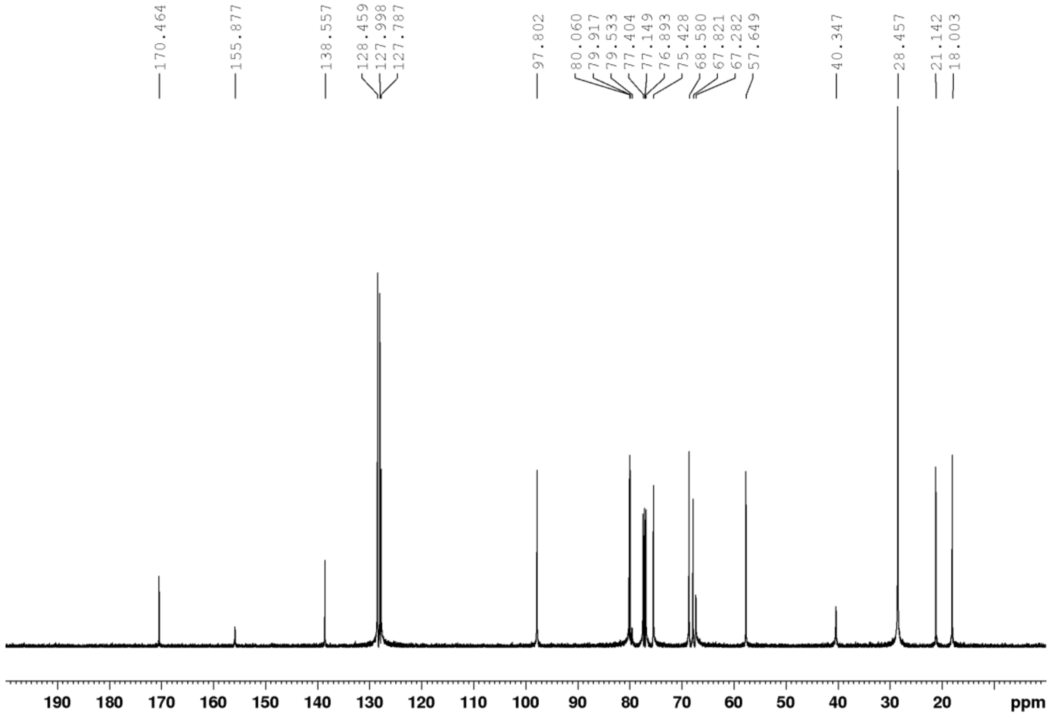

^{1}H NMR, **3** (500 MHz, $CDCl_3$)

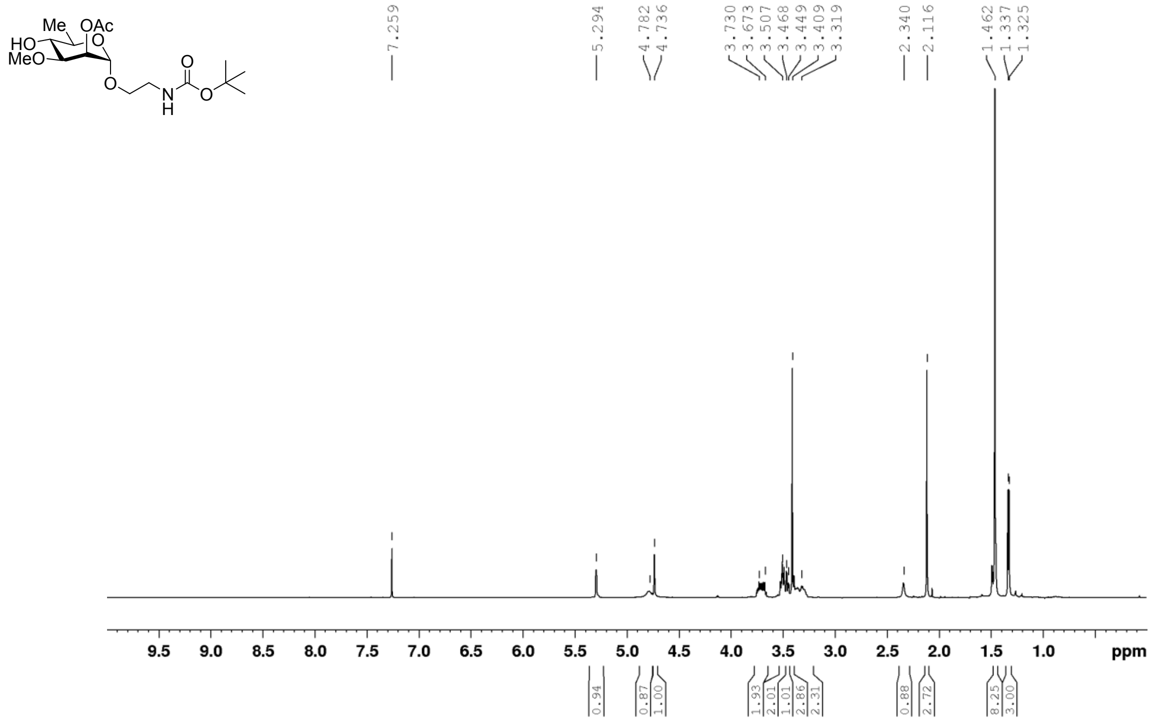

^{13}C {H} NMR, **3** (125 MHz, $CDCl_3$)

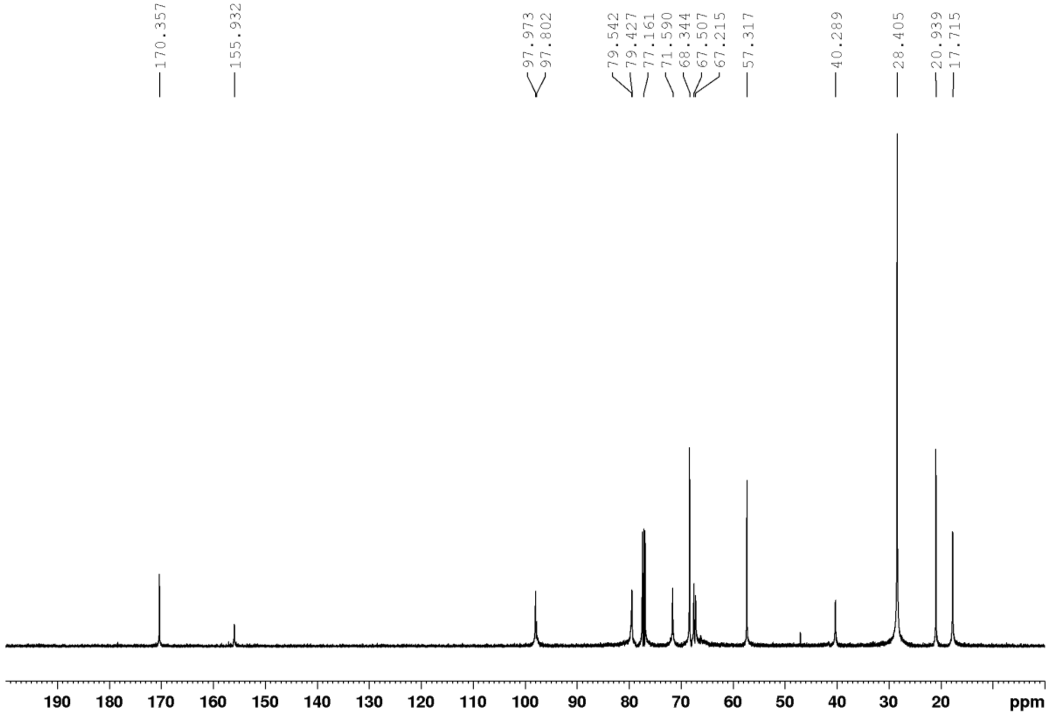

^{1}H NMR, **4 di** (400 MHz, $CDCl_3$)[13]

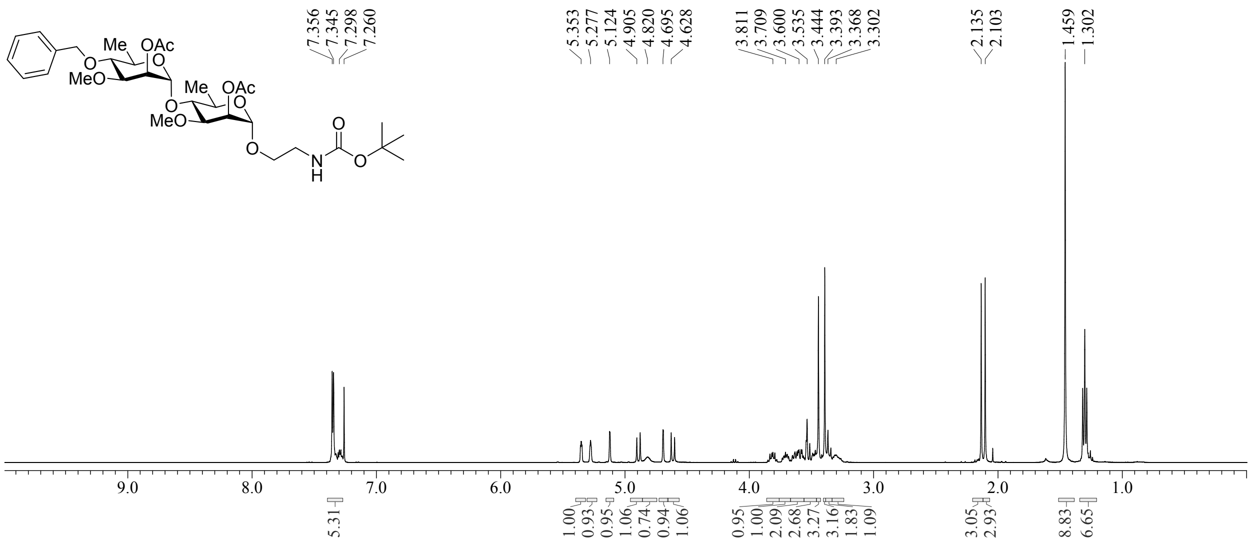

^{13}C {H} NMR, **4 di** (125 MHz, $CDCl_3$)

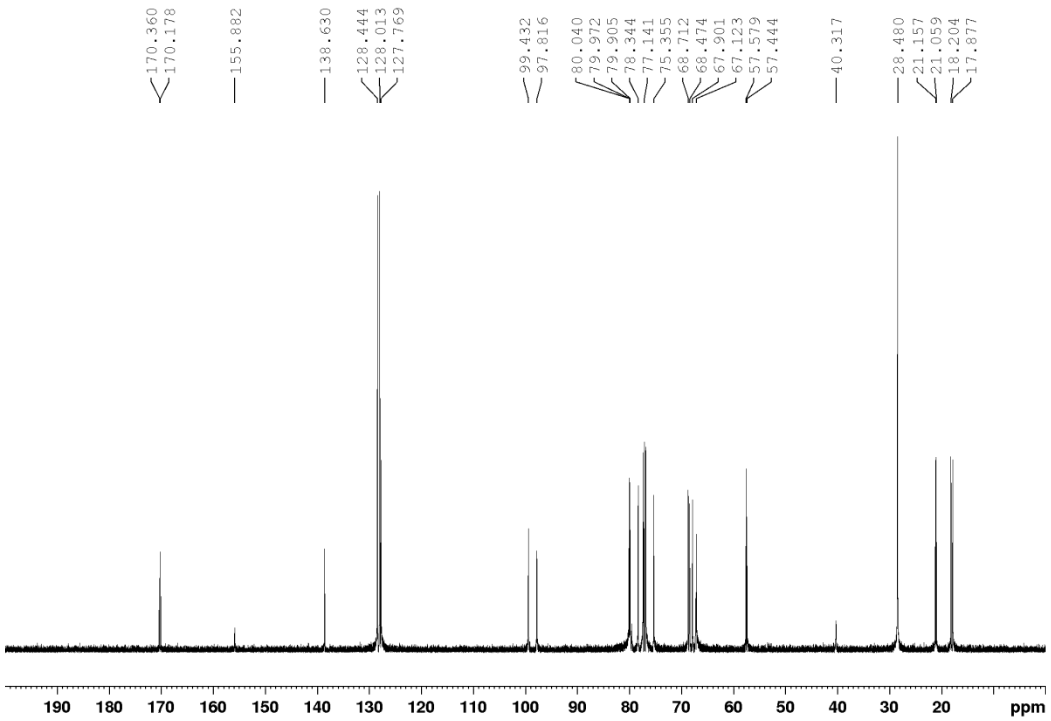

^{1}H NMR, **4 tri** (600 MHz, $CDCl_3$)

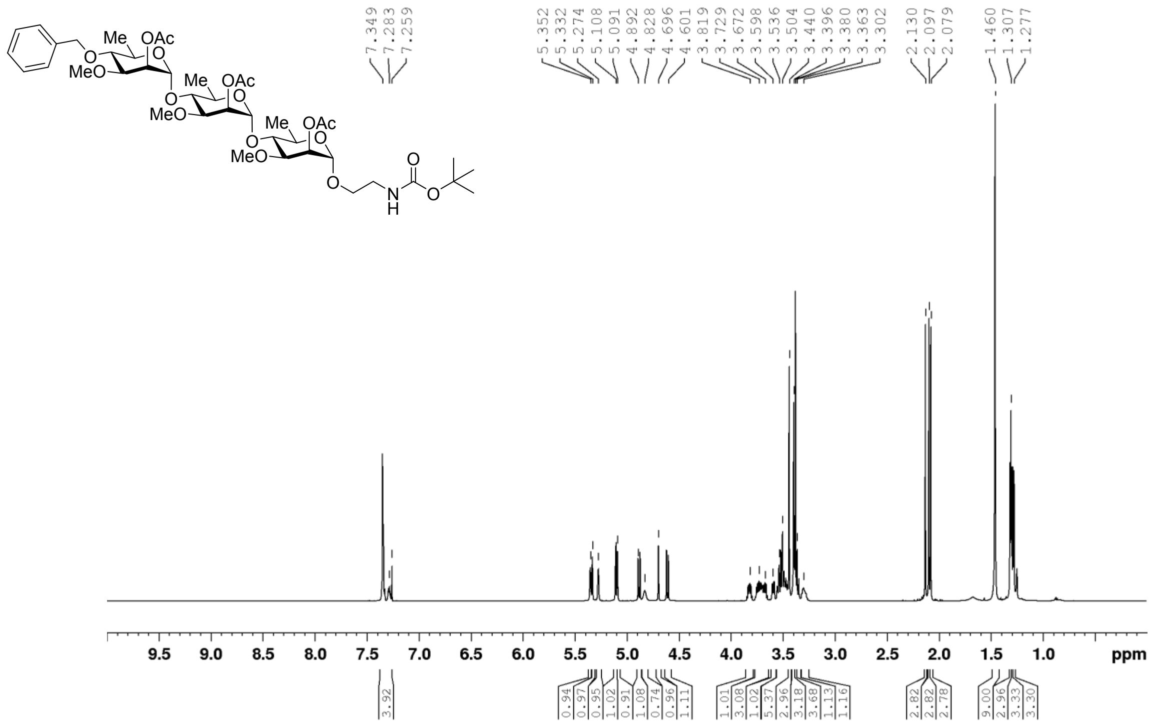

^{13}C {H} NMR, **4 tri** (150 MHz, $CDCl_3$)

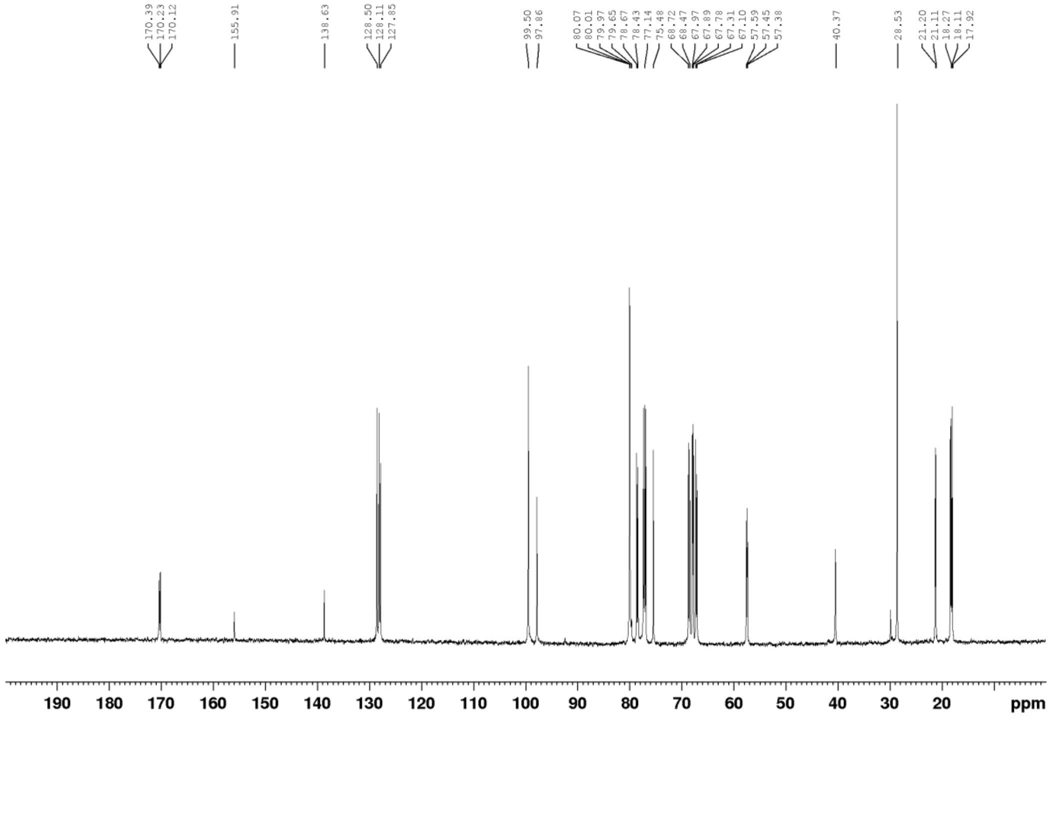

^{1}H NMR, **4 tetra** (600 MHz, $CDCl_3$)

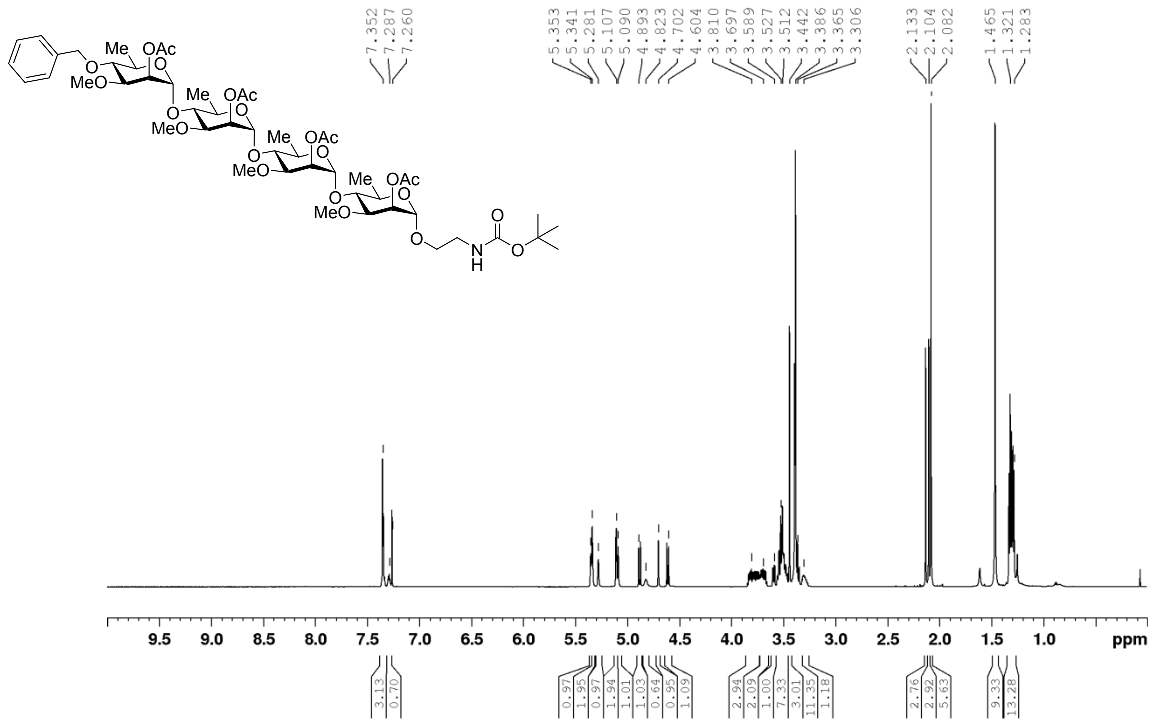

^{13}C {H} NMR, **4 tetra** (150 MHz, $CDCl_3$)

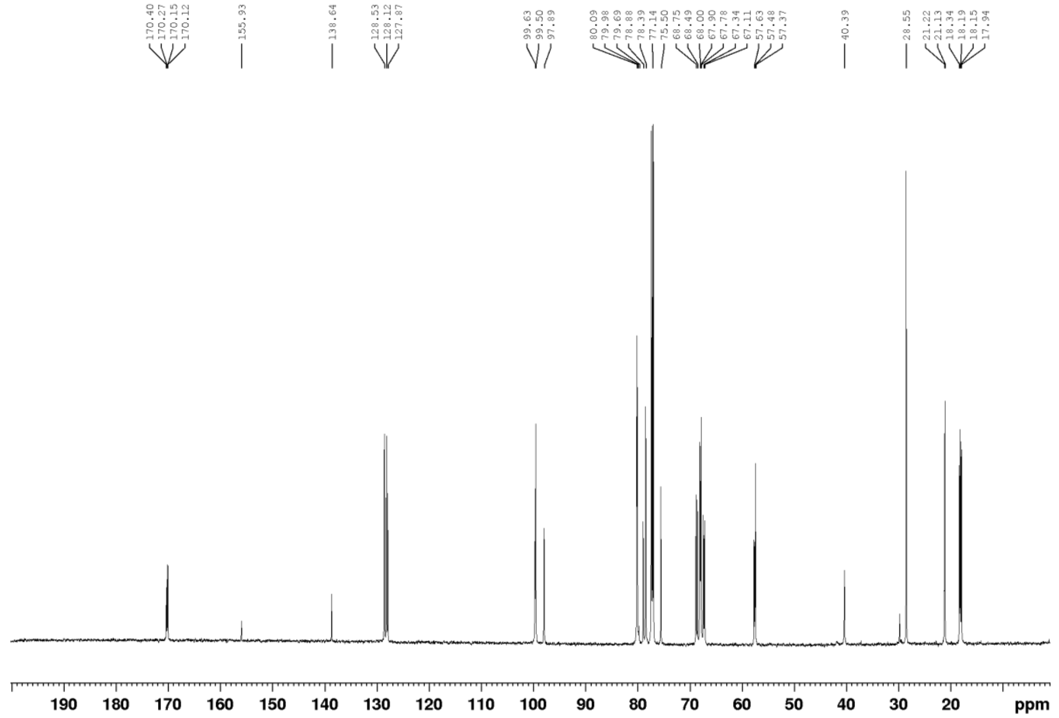

^{1}H NMR, **4 penta** (600 MHz, $CDCl_3$)

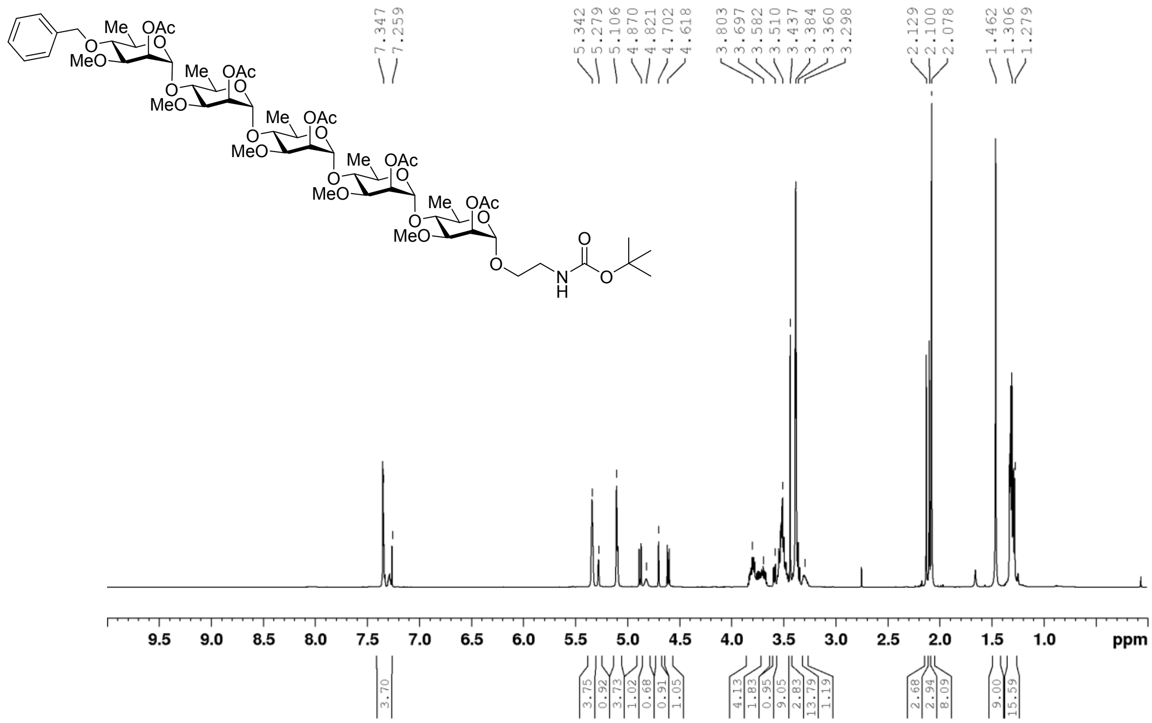

^{13}C {H} NMR, **4 penta** (150 MHz, $CDCl_3$)

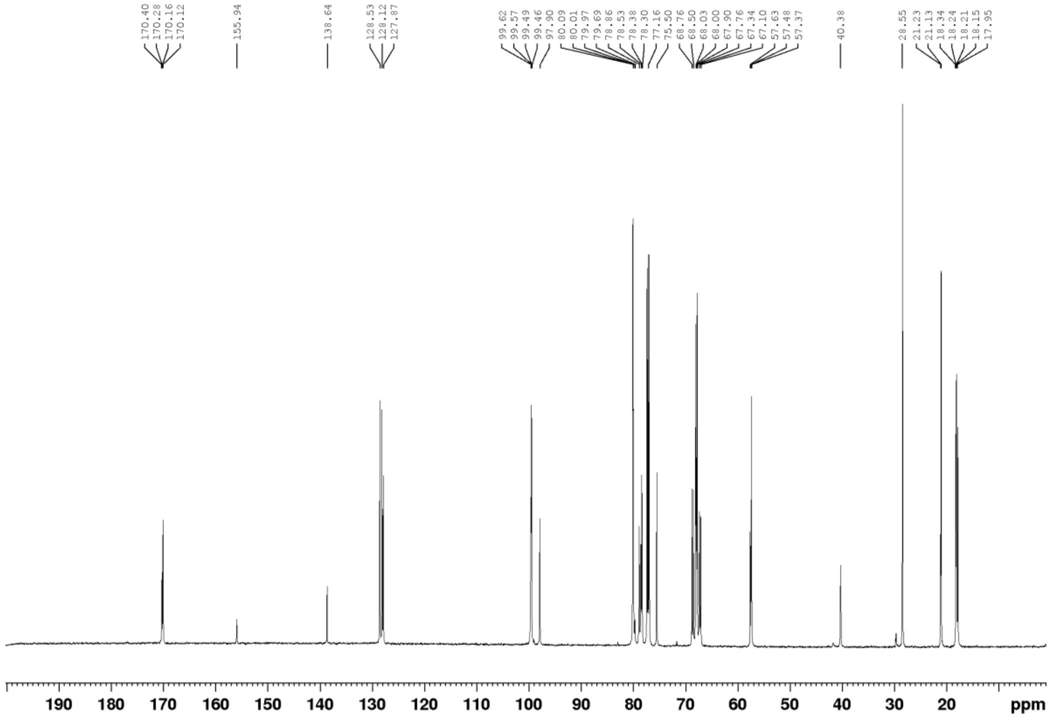

^{1}H NMR, **5 di** (600 MHz, $CDCl_3$)

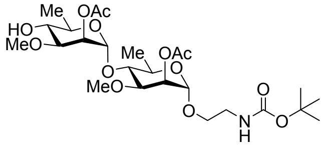

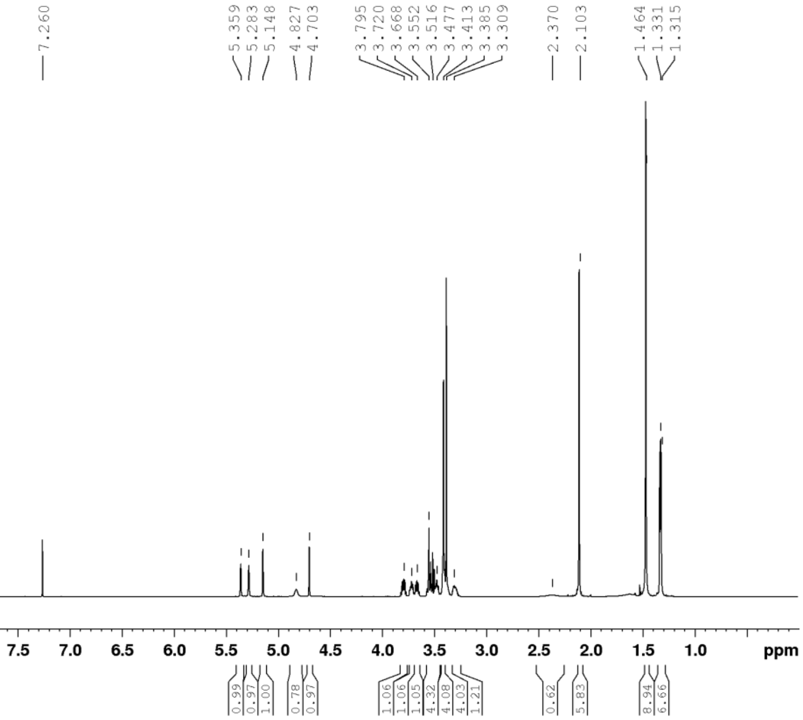

^{13}C {H} NMR, **5 di** (150 MHz, $CDCl_3$)

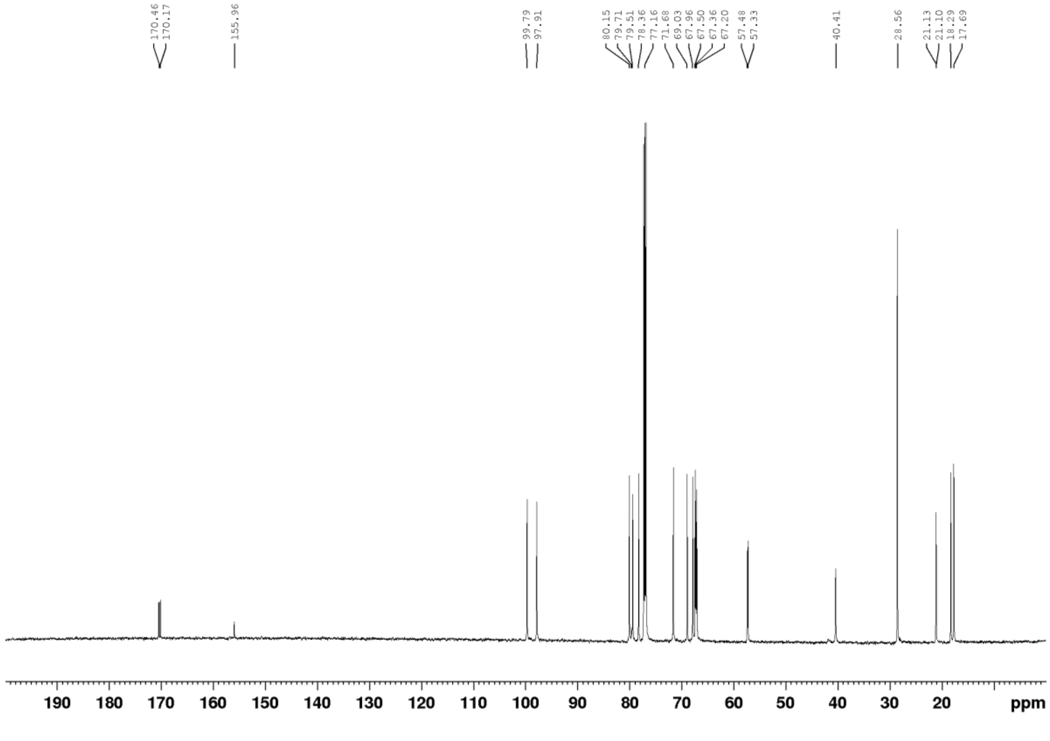

^{1}H NMR, **5 tri** (600 MHz, CD_3OD)

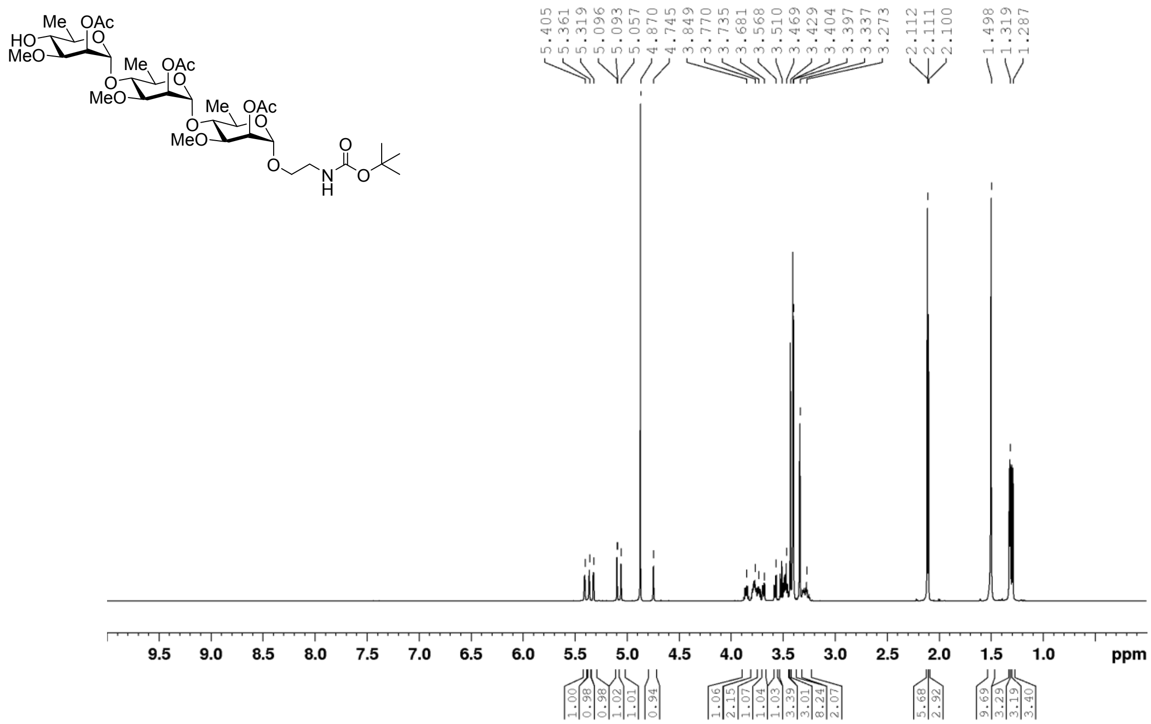

^{13}C {H}, **5 tri** (150 MHz, CD_3OD)

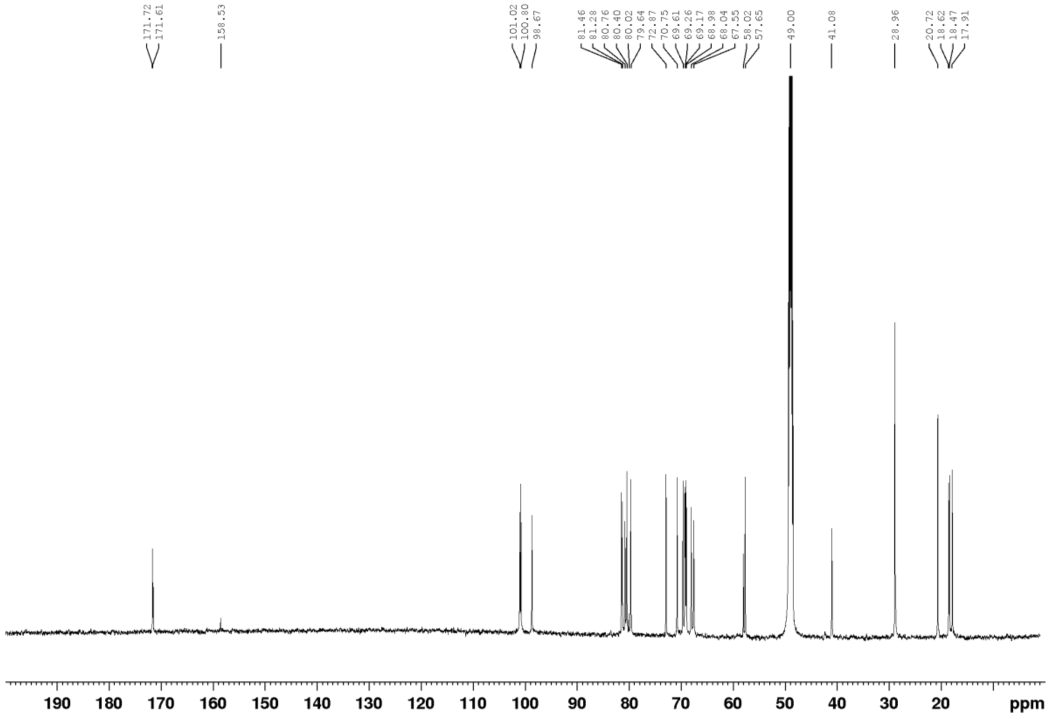

^{1}H NMR, **5 tetra** (600 MHz, CD_3OD)

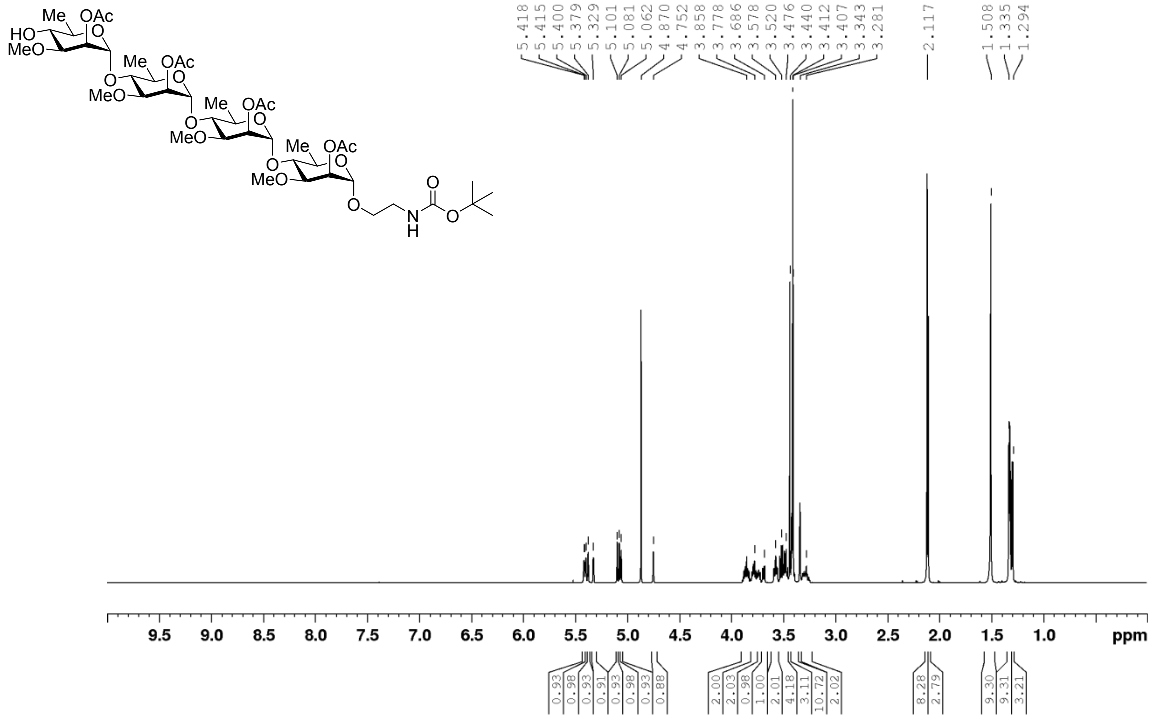

^{13}C {H} NMR, **5 tetra** (150 MHz, CD_3OD)

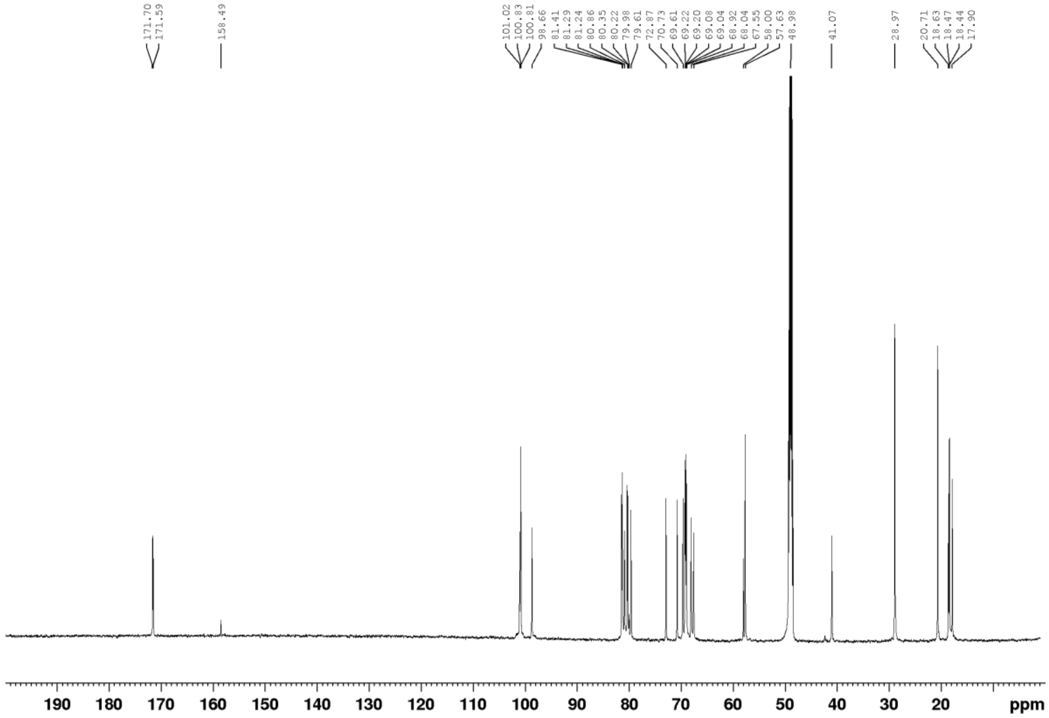

^{1}H NMR, **5 penta** (600 MHz, CD_3OD)

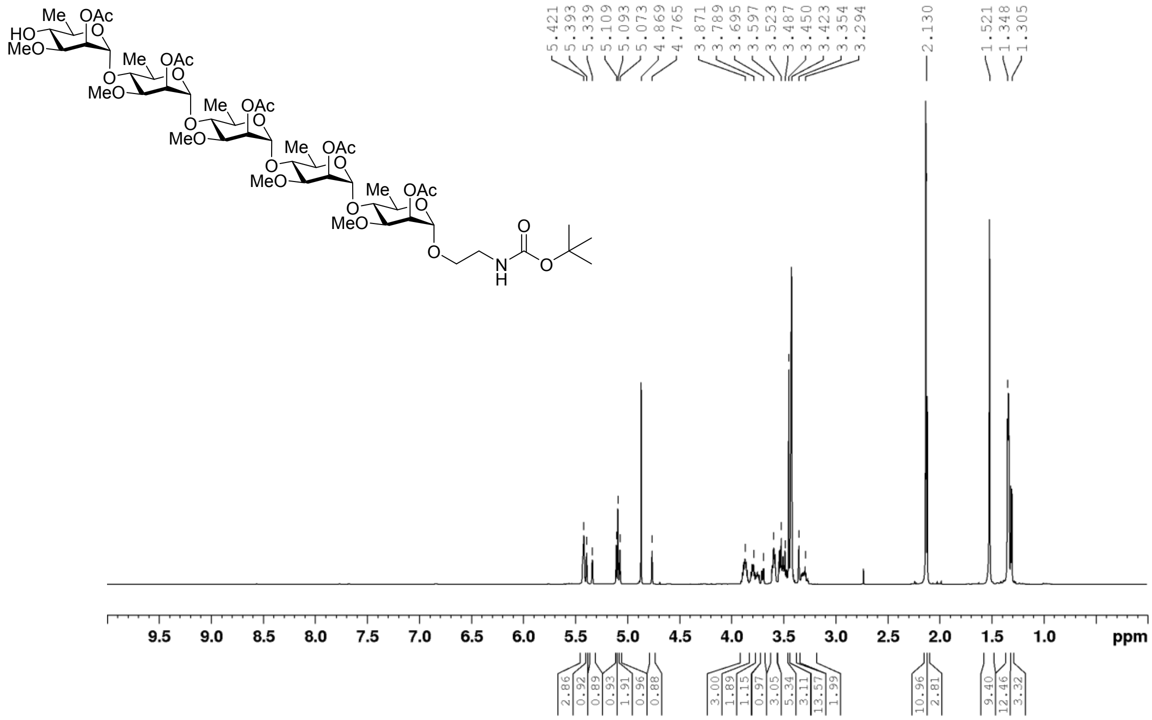

^{13}C {H} NMR, **5 penta** (150 MHz, CD_3OD)

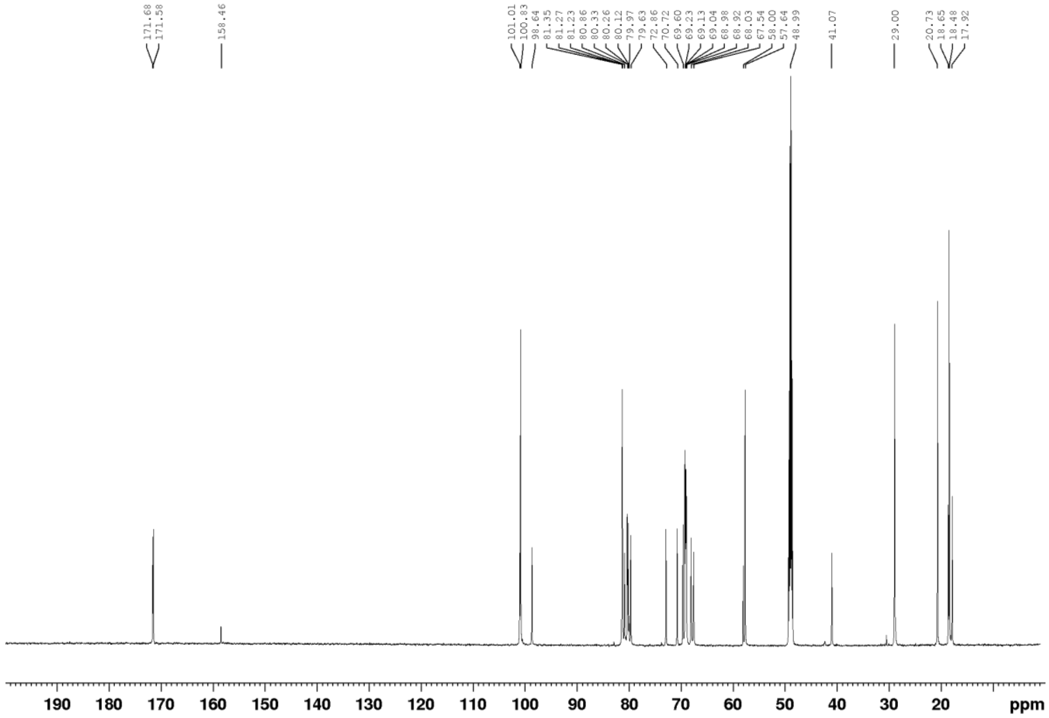

^{1}H NMR, **6 tri** (600 MHz, CD_3OD)

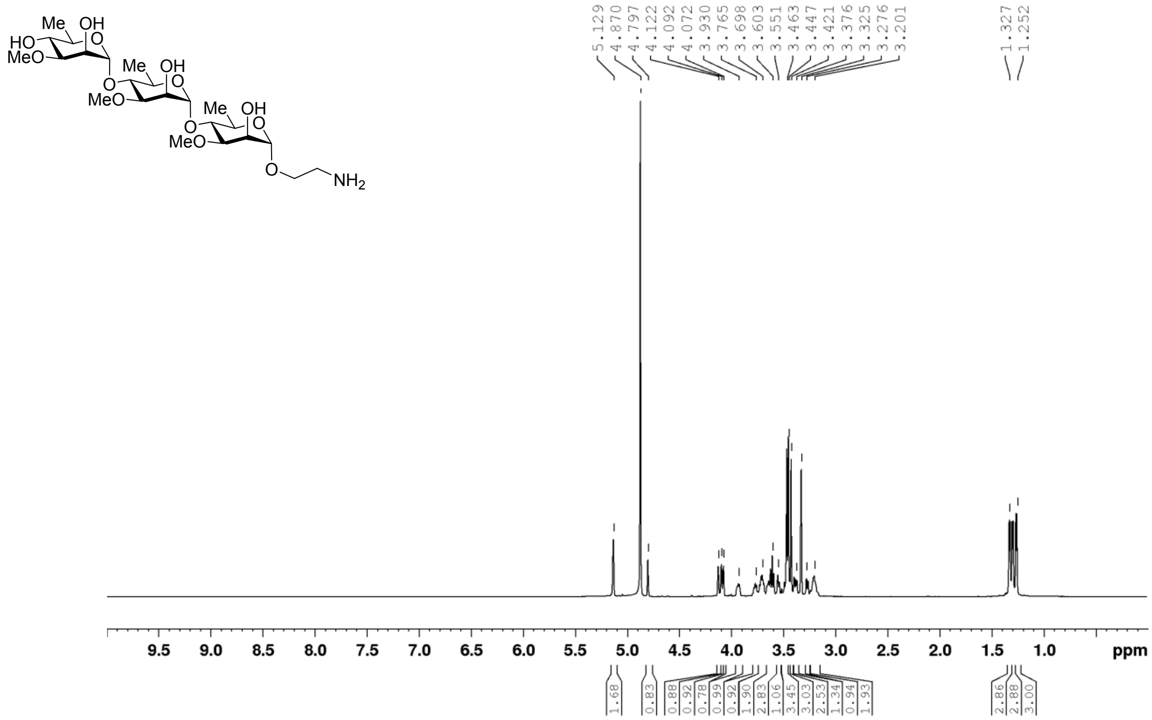

^{13}C {H} NMR, **6 tri** (150 MHz, CD_3OD)

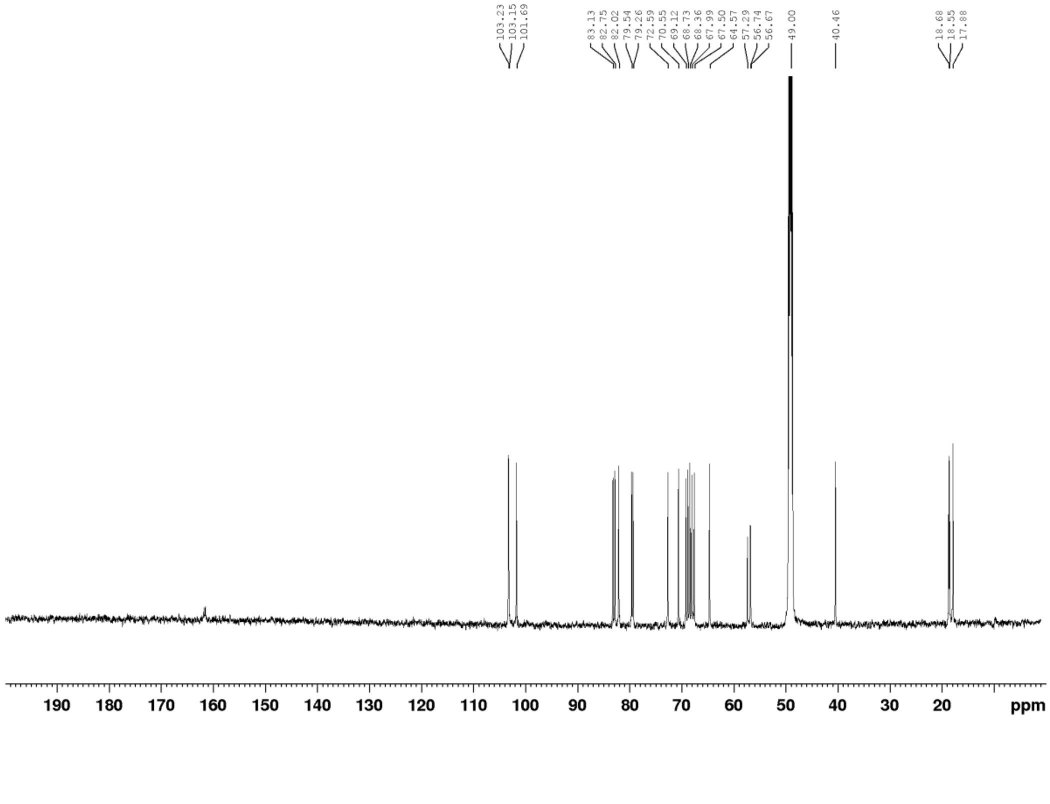

^{1}H NMR, **6 tetra** (600 MHz, CD_3OD)

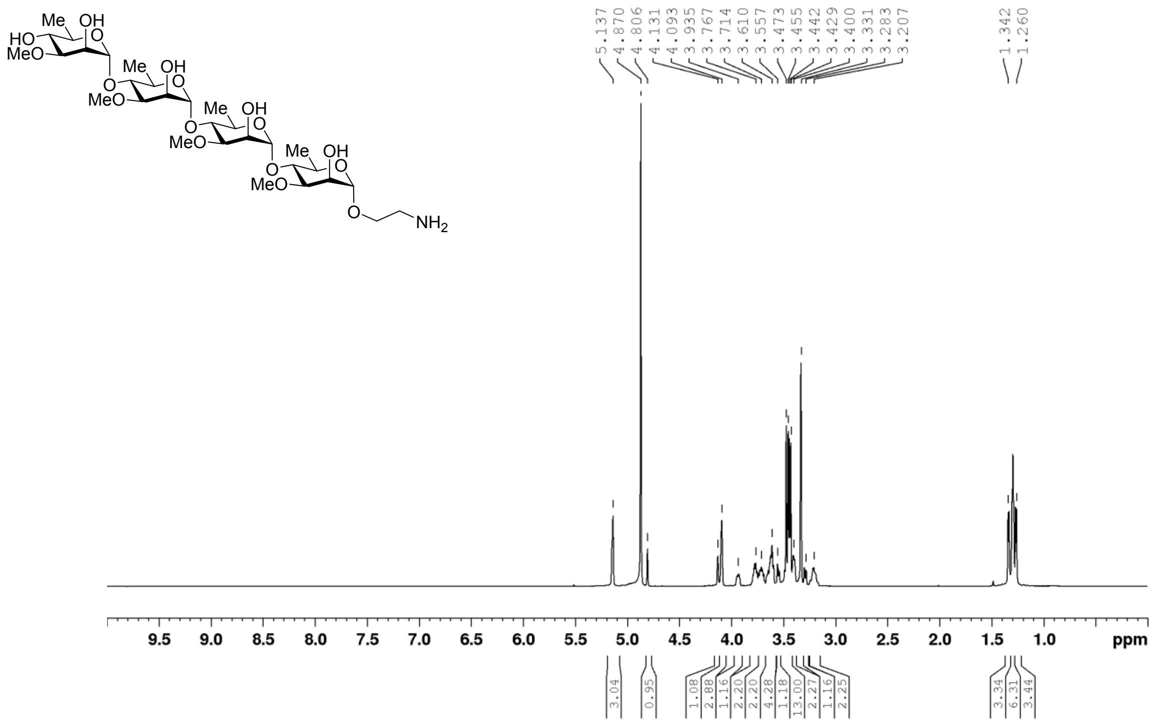

^{13}C {C} NMR, **6 tetra** (150 MHz, CD_3OD)

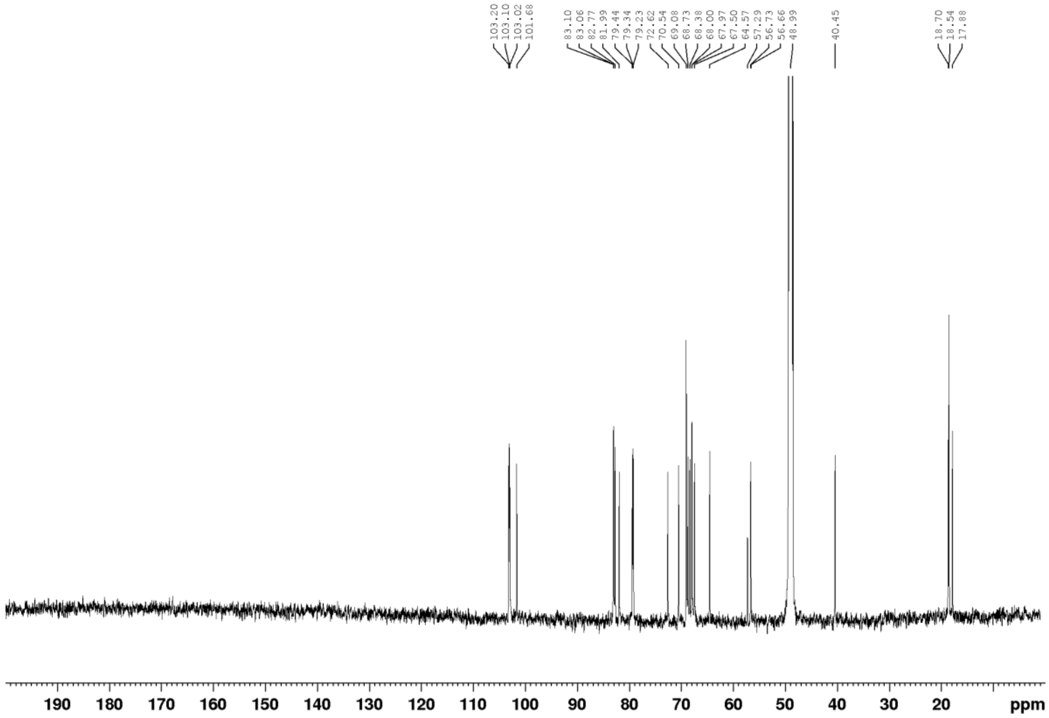

^{1}H NMR, **6 penta** (600 MHz, CD_3OD)

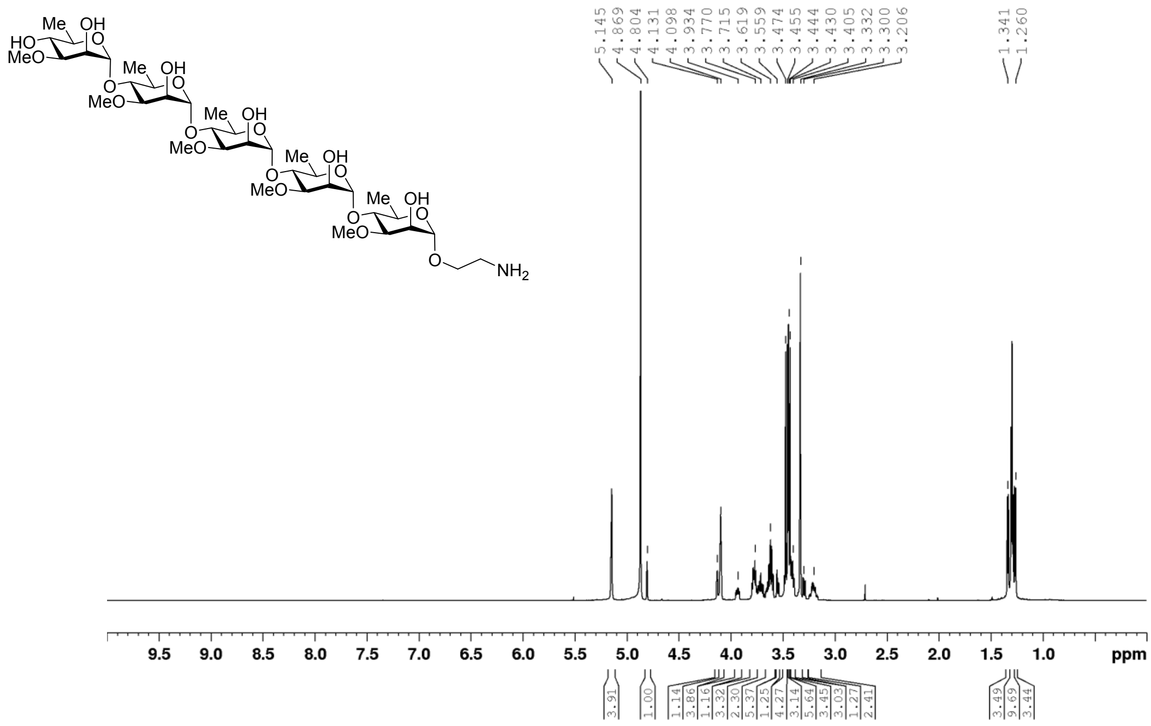

^{13}C NMR {H} NMR, **6 penta** (150 MHz, CD_3OD)

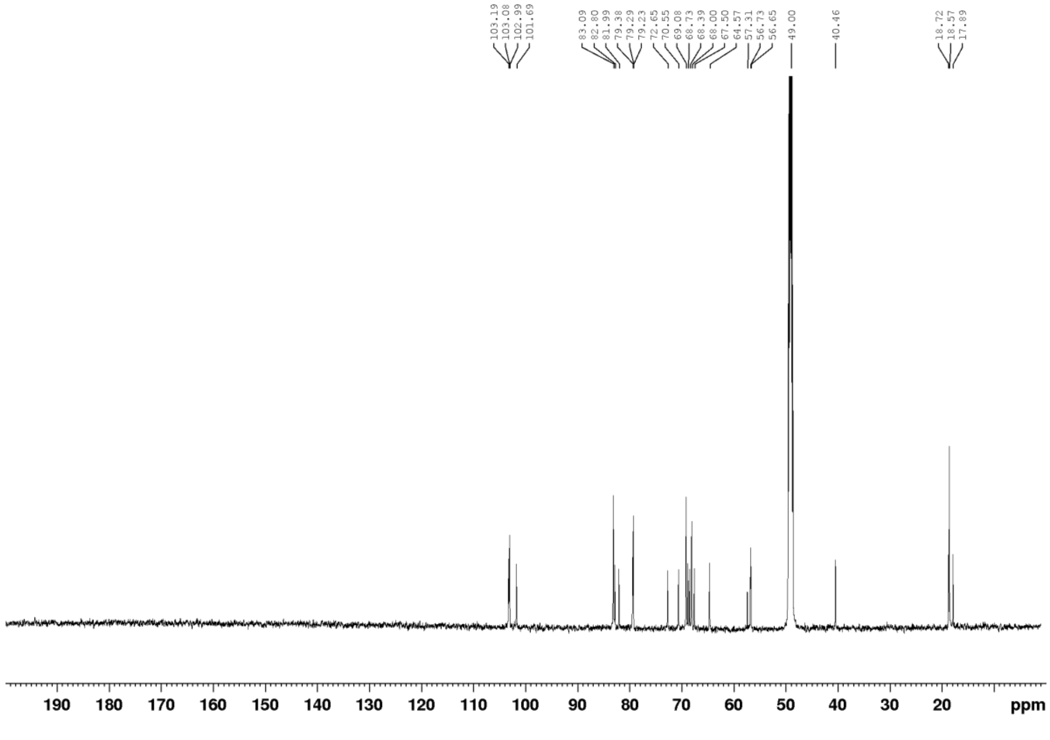

^{1}H NMR, **1 tri** (600 MHz, CD_3OD)

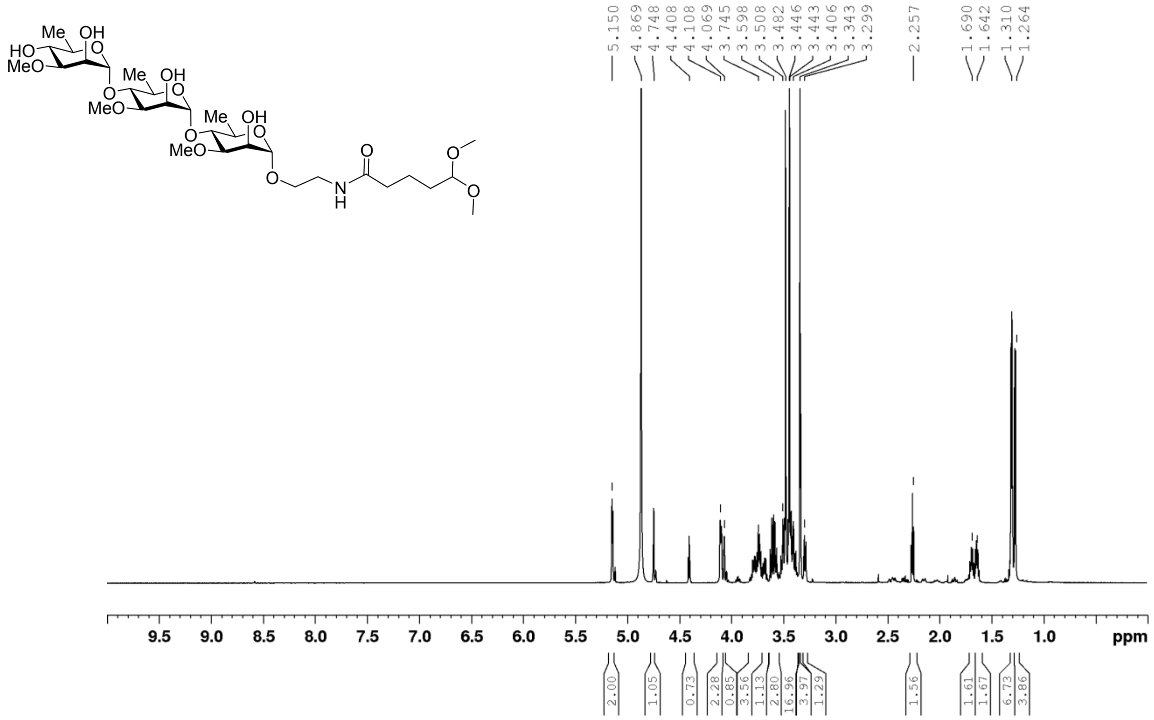

^{13}C {H} NMR, **1** tri (150 MHz, CD_3OD)

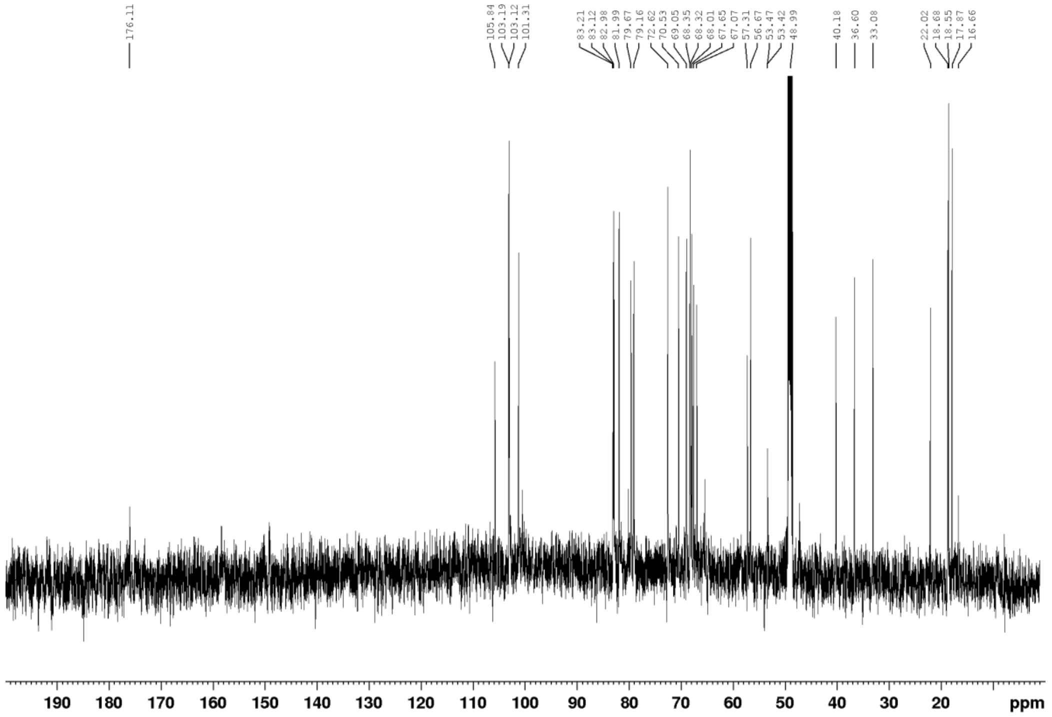

^{1}H NMR, **1 tetra** (600 MHz, CD_3OD)

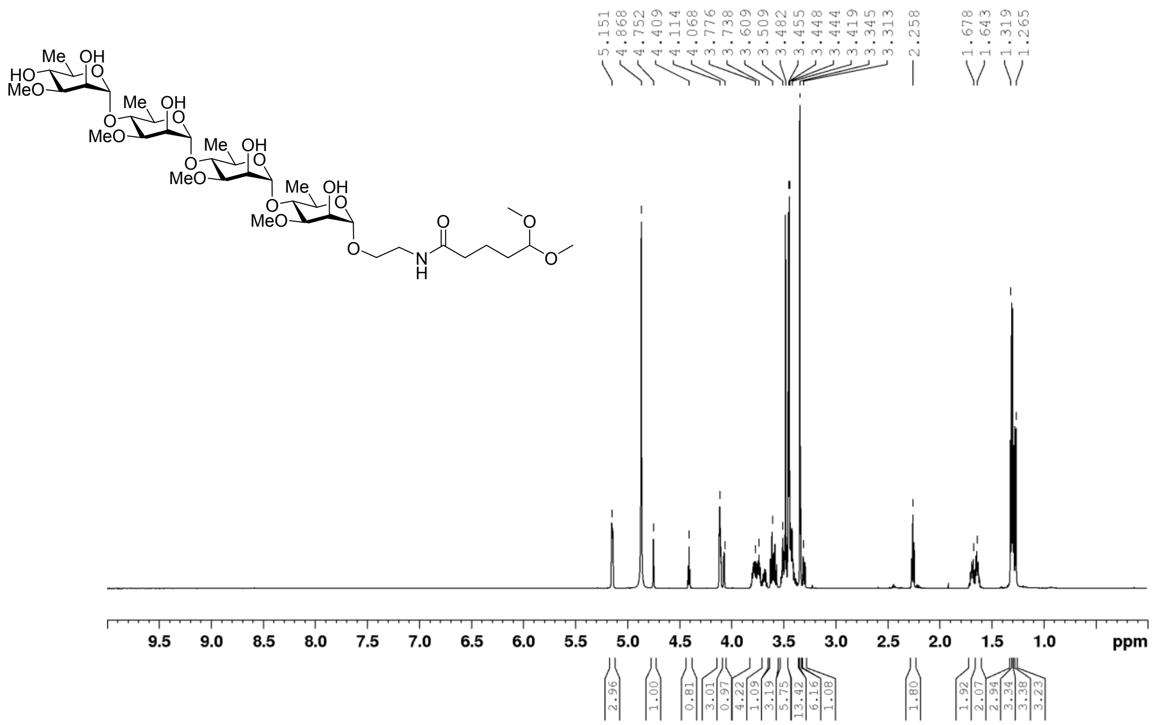

^{13}C {H} NMR, **1 tetra** (150 MHz, CD_3OD)

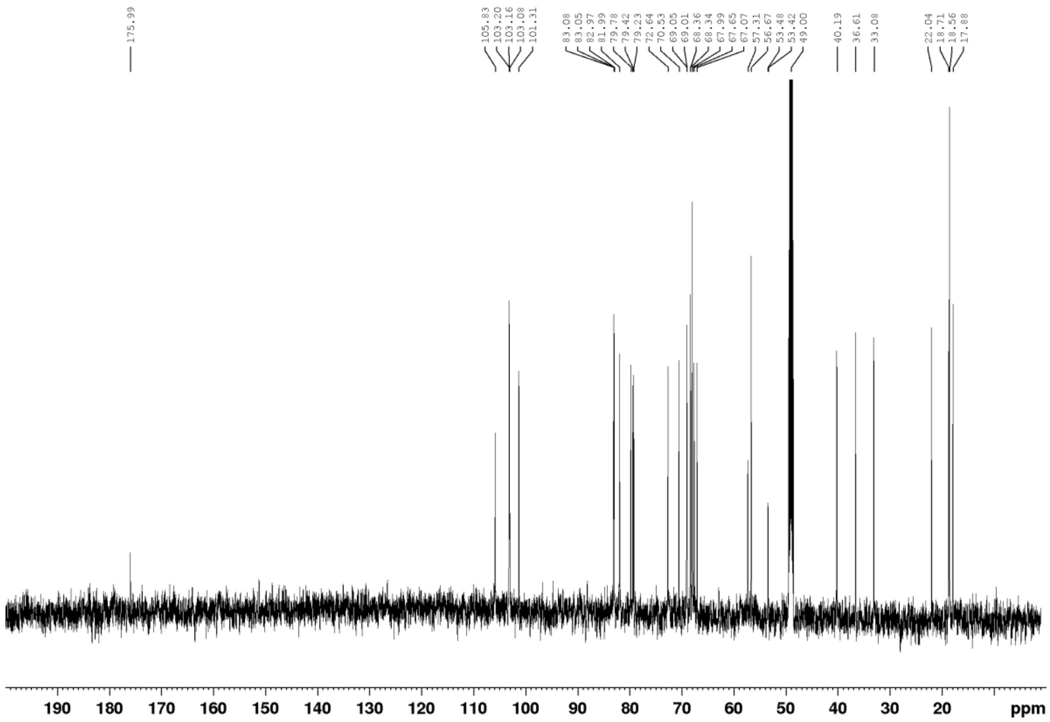

^{1}H NMR, **1 penta** (600 MHz, CD_3OD)

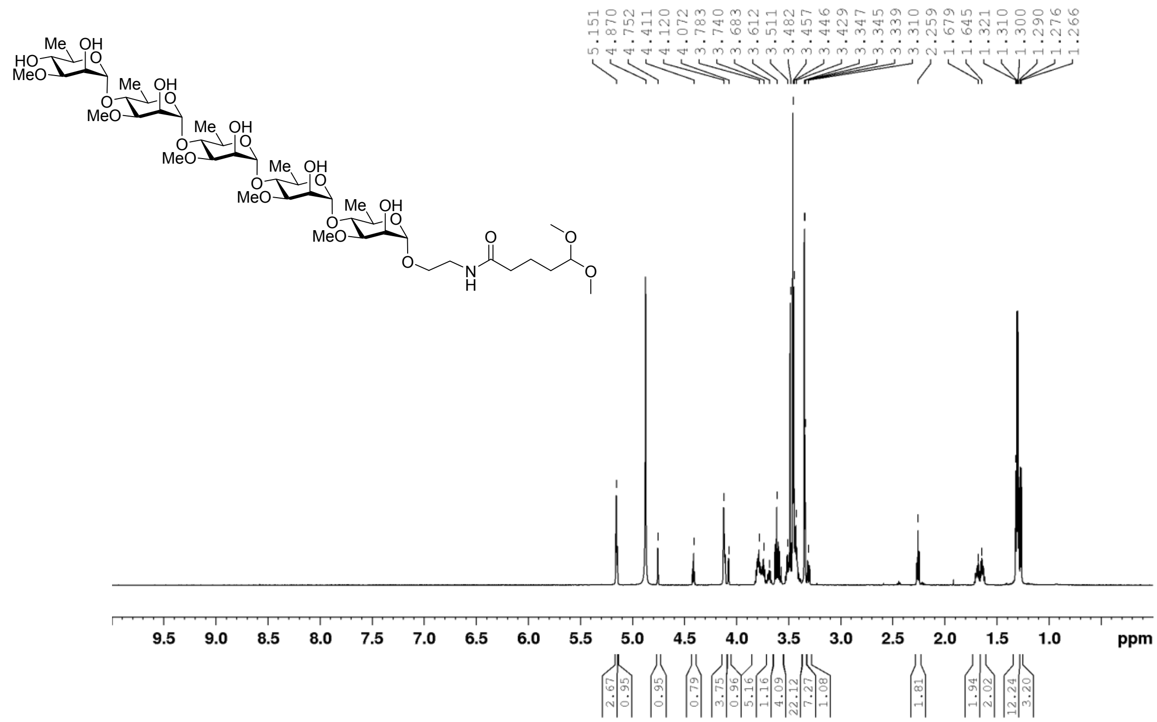

^{13}C {H} NMR, **1 penta** (150 MHz, CD_3OD)

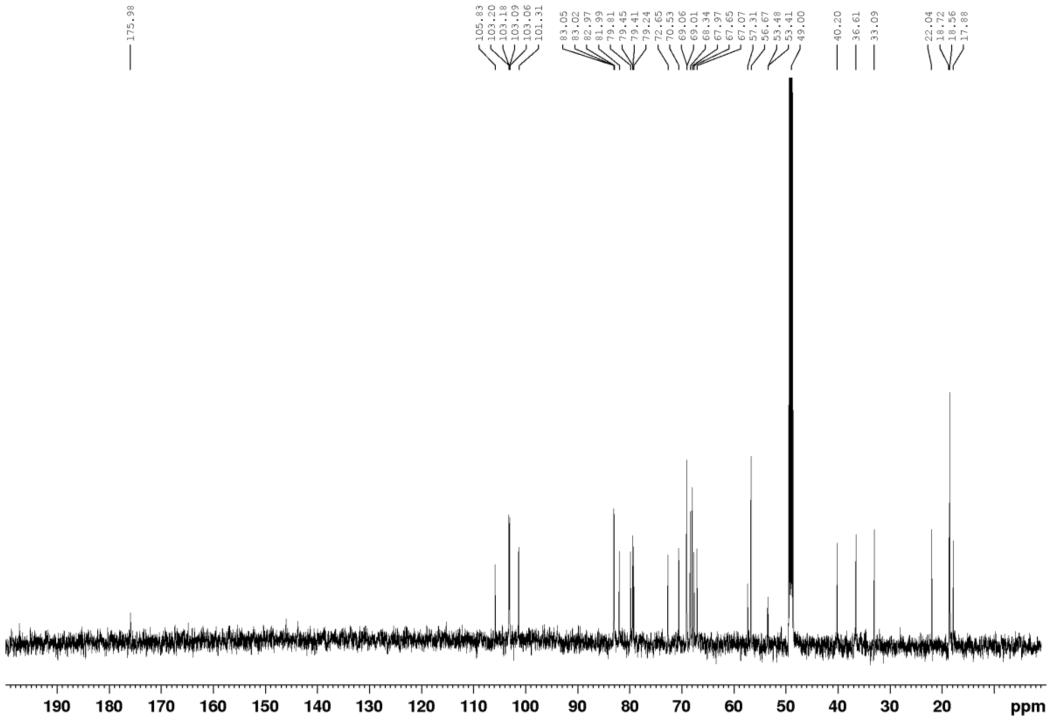

MALDI Spectra

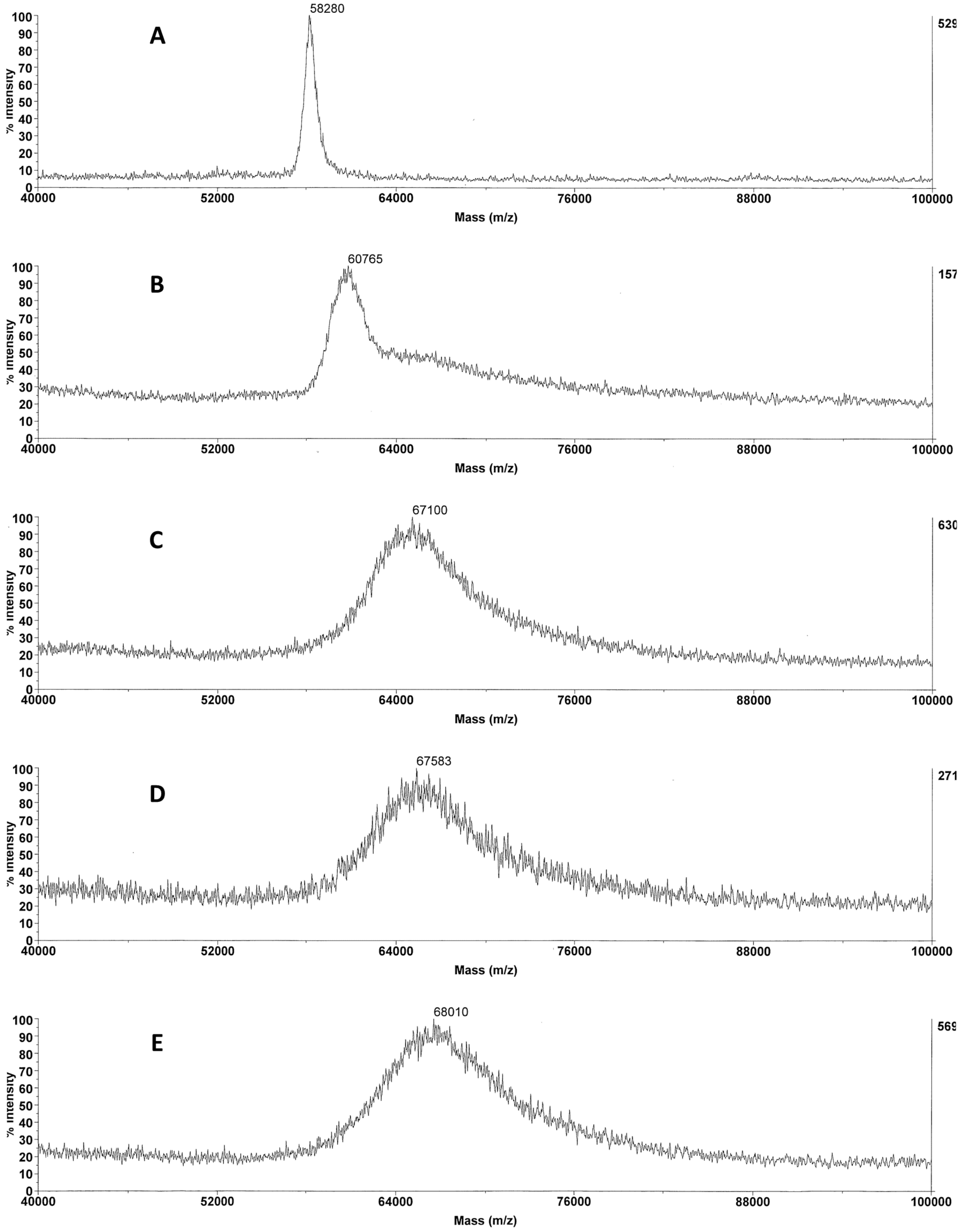

Figure S15 MALDI data of the various steps of CRM conjugation steps. (A) CRM, (B) CRM-oxy, (C) CRM-tri, (D) CRM-tetra, and (E) CRM-penta

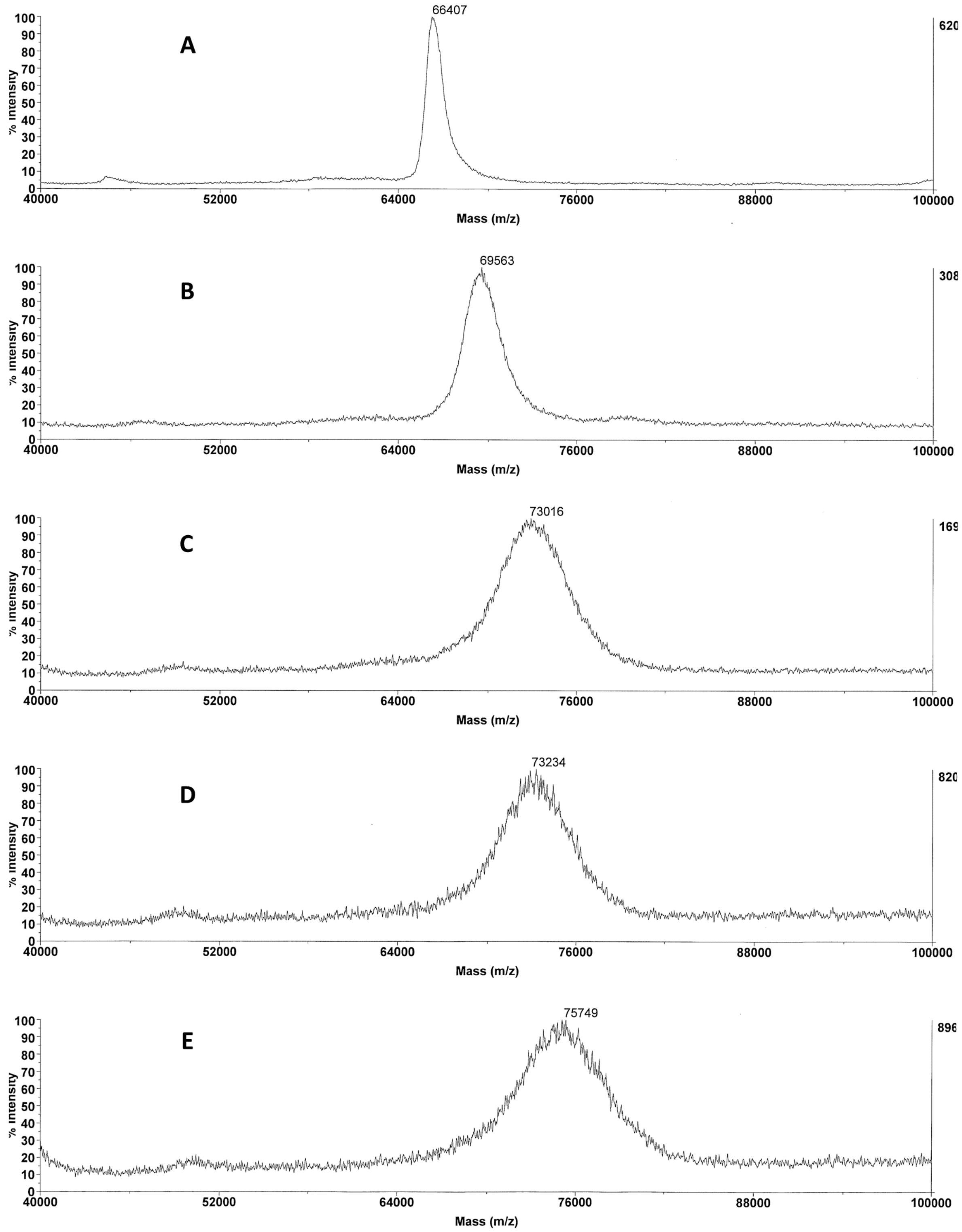

Figure S16 MALDI data of the various steps of BSA conjugation steps. (A) BSA, (B) BSA-oxy, (C) BSA-tri, (D) BSA-tetra, and (E) BSA-penta

References:

1. Jamshidi, M. P., Cairns, C., Chong, S., Michael, F. St., Vinogradov, E. V., Cox, A. D., Sauvageau, J. Synthesis and Immunogenicity of a Methyl Rhamnan Pentasaccharide Conjugate from *Pseudomonas aeruginosa* A-Band Polysaccharide, ACS Infect. Dis. 8 (2022) 1347-1355.
2. Meng, S., Tian, T., Han, D., Wang, L., Tang, S., Meng, X., Li, Z., Efficient assembly of oligomannosides using the hydrophobically assisted switching phase method, Org. Biomol. Chem. 13 (2015), 6711-6722.
3. Zhang, X., Wang, D., Jin, G., Wang, L., Guo, Z., Gu, G. Synthesis of a tetrasaccharide repeating unit of the exopolysaccharide from *Burkholderia multivorans*, J. Carb. Chem. 36 (2017) 189-204.
4. Wang, D., Zhuge, W., Guo, Z., Gu, G. Synthesis of a disaccharide repeating unit of the O-antigen from *Burkholderia ambifaria* and its oligomers, Carb. Res. 442 (2017) 41-51.
